# Plastid 16S rRNA Gene Diversity among Eukaryotic Picophytoplankton Sorted by Flow Cytometry from the South Pacific Ocean

**DOI:** 10.1371/journal.pone.0018979

**Published:** 2011-04-28

**Authors:** Xiao Li Shi, Cécile Lepère, David J. Scanlan, Daniel Vaulot

**Affiliations:** 1 UPMC (Paris-06) and CNRS, UMR 7144, Station Biologique de Roscoff, Roscoff, France; 2 School of Life Sciences, University of Warwick, Coventry, United Kingdom; 3 State Key Laboratory of Lake Science and Environment, Nanjing Institute of Geography and Limnology, Chinese Academy of Sciences, Nanjing, People's Republic of China; Mt. Alison University, Canada

## Abstract

The genetic diversity of photosynthetic picoeukaryotes was investigated in the South East Pacific Ocean. Genetic libraries of the plastid 16S rRNA gene were constructed on picoeukaryote populations sorted by flow cytometry, using two different primer sets, OXY107F/OXY1313R commonly used to amplify oxygenic organisms, and PLA491F/OXY1313R, biased towards plastids of marine algae. Surprisingly, the two sets revealed quite different photosynthetic picoeukaryote diversity patterns, which were moreover different from what we previously reported using the 18S rRNA nuclear gene as a marker. The first 16S primer set revealed many sequences related to Pelagophyceae and Dictyochophyceae, the second 16S primer set was heavily biased toward Prymnesiophyceae, while 18S sequences were dominated by Prasinophyceae, Chrysophyceae and Haptophyta. Primer mismatches with major algal lineages is probably one reason behind this discrepancy. However, other reasons, such as DNA accessibility or gene copy numbers, may be also critical. Based on plastid 16S rRNA gene sequences, the structure of photosynthetic picoeukaryotes varied along the BIOSOPE transect vertically and horizontally. In oligotrophic regions, Pelagophyceae, Chrysophyceae, and Prymnesiophyceae dominated. Pelagophyceae were prevalent at the DCM depth and Chrysophyceae at the surface. In mesotrophic regions Pelagophyceae were still important but Chlorophyta contribution increased. Phylogenetic analysis revealed a new clade of Prasinophyceae (clade 16S-IX), which seems to be restricted to hyper-oligotrophic stations. Our data suggest that a single gene marker, even as widely used as 18S rRNA, provides a biased view of eukaryotic communities and that the use of several markers is necessary to obtain a complete image.

## Introduction

Picoeukaryotic phytoplankton with cell size less than 2–3 µm is widely distributed in the world ocean. In the last decade, photosynthetic picoeukaryotes have received more attention since they are very important contributors to primary production due to high cell-specific carbon fixation rates [1]–[3]. The use of molecular approaches, especially sequencing of the 18S rRNA gene, has greatly improved our understanding of the diversity and distribution of marine picoeukaryotic phytoplankton [4], [5]. However several studies have shown that clone libraries constructed with the 18S rRNA gene are heavily biased towards heterotrophic cells [6], [7]. In order to focus on phototrophs, several strategies have been developed, including targeting plastid genes such as 16S rRNA [8], [9] or *psbA* [10], using taxonomic biased 18S rRNA gene primers [11], or sorting photosynthetic populations by flow cytometry before constructing 18S rRNA gene clone libraries [12], [13]. These three different approaches have led to the common conclusion that small photosynthetic eukaryotes are more diverse than previously thought and have highlighted the importance in open ocean waters of three major taxonomic groups: Prasinophyceae (in particular novel order-level clades), Chrysophyceae, and Prymnesiophyceae. Still, the plastid and the nuclear gene approaches do not provide exactly the same image. For example, Prasinophyceae are better represented in nuclear than in plastid rRNA clone libraries [9], [12]. However, to date, these two approaches have not been compared on the same sample set.

In the present paper we constructed plastid 16S rRNA gene clone libraries from flow cytometry sorted samples from the South East Pacific Ocean for which we had already constructed 18S rRNA clone libraries [12]. Such samples are particularly appropriate for such a comparison because firstly, they are highly enriched in photosynthetic sequences and secondly, their diversity is reduced compared to bulk filtered samples [13]. Moreover, in this specific case, plastid 16S rRNA clone libraries have also been analyzed on filtered samples [9]. Another interest of this sample set is that the South East Pacific Ocean presents very large trophic gradients [14] that translate into wide differences in the composition of the small photosynthetic eukaryote community among the different sampled stations. Our results demonstrate marked differences between community structures as assessed from the plastid rRNA gene compared to the nuclear one, as well as differences when two different plastid rRNA primer sets are used, suggesting that caution should be exercised when interpreting diversity data based on a single gene and/or single primer set.

## Materials and Methods

### Sampling

**Figure 1:**
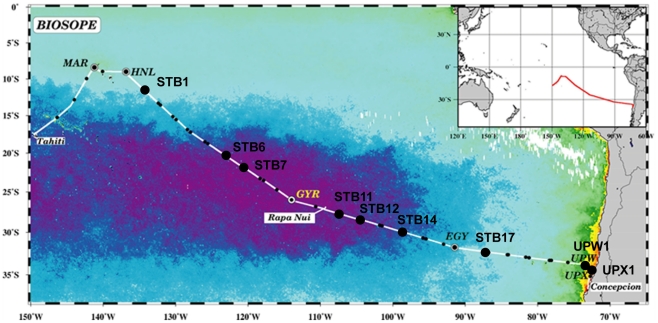
Map of stations sampled in the South East Pacific Ocean during the BIOSOPE cruise (2004). BIOSOPE cruise track map superimposed on a SeaWiFS ocean colour composite, the dark purple indicating extremely low values (0.018 mg m^−3^) of total chlorophyll *a*. Black dots indicate the stations analysed by 16S rRNA gene clone libraries.

Sampling was performed in the surface layer and in the vicinity of the Deep Chlorophyll Maximum (DCM) at selected stations between 26 October and 11 December 2004 along a transect through the South East Pacific Ocean (Figure 1 and Table 1) during the BIOSOPE cruise on board the research vessel L'Atalante. Seawater samples were collected using Niskin bottles mounted on a CTD frame. Samples were concentrated between 5 and 100-fold by tangential flow filtration using a 100 000 MWCO (Regenerated Cellulose- RC ref VF20C4) Vivaflow 200 cassette. A previous study [13] showed that the average recovery for picoeukaryotes was 57% and that delicate cells such as cryptophytes were recovered.

**Table 1:** BIOSOPE sample locations, Photosynthetic picoeukaryote (PPE) abundance [37], and clone library information.

StationName	Longitude°W	Latitude°S	Trophicstatus	SampleCode	Depth(m)	PPE concentration(cell mL^−1^)	Number of PPE cells sorted	NestedPCR	Clone LibraryOXY107FPlastidSequence #	Clone LibraryPLA491FPlastidSequence #
STB1	134.10	11.74	Meso	T17	90	4 049	91 407	No	11	ND
				T19	25	7 017	103 074	No	4	ND
STB6	122.89	20.45	Oligo	T33	180	2 453	225 500	No	22	29
				T35	55	615	151 900	No	24	27
STB7	120.38	22.05	Oligo	T39	175	919	106 000	No	23	28
				T41	40	791	125 000	No	19	19
STB11	107.29	27.77	Oligo	T58	200	1 519	168 000	No	45	24
				T60	0	1 305	171 440	No	21	28
STB12	104.31	28.54	Oligo	T65	40	861	123 000	Yes		62
STA14	98.39	30.04	Oligo	T84	5	1 007	118 000	No	25	28
				T88	150	3 974	236 500	No	35	30
STB17	86.78	32.40	Meso	T120	70	6 720	204 000	No	18	36
				T123	20	6 802	124 000	No	23	32
UPW1	73.37	34.00	Eutro	T148	35	4 820	81 000	No	3	20

Oligo = oligotrophic, Meso = mesotrophic, Eutro = eutrophic (ND = not determined).

### Flow cytometry analysis and sorting

Concentrated samples were analyzed on board using a FACSAria flow cytometer (Becton Dickinson, San Jose, CA, USA) equipped with a laser emitting at 488 nm and the normal filter setup. The signal was triggered on the red fluorescence from chlorophyll. Photosynthetic picoeukaryotes were discriminated based on their small side scatter, the presence of red chlorophyll fluorescence, and the absence of orange phycoerythrin fluorescence, and sorted in “purity” mode. Cells were collected into Eppendorf tubes and, after a quick centrifugation, the volume of sorted samples was adjusted to 250 µL by adding filtered seawater. Samples were deep frozen in liquid nitrogen.

### DNA extraction, PCR reaction and cloning

DNA from sorted picoeukaryote populations was extracted using the DNeasy blood and tissue kit (Qiagen), as recommended by the manufacturer. The plastid 16S rRNA gene was amplified by the polymerase chain reaction (PCR) using the primer sets PLA491F/OXY1313R and OXY107F/OXY1313R [8], [15], [16]. The PCR mixture (50 µl final volume) contained 5 µl of extracted DNA with 0.5 µM final concentration of each primer, 1.25 mM MgCl_2_, 1.25 mM dNTP, 1.25 U GoTaq® DNA polymerase and 1×GoTaq® Flexi green Buffer (Promega). The amplification conditions comprised steps at 95°C for 5 min, and 40 cycles of 95°C for 45 s, 60°C for 45 s, and 72°C for 1 min and 15 s, as well as 7 min at 72°C for polymerase extension. For sample T65, the plastid 16S rRNA gene was amplified using universal 16S rRNA gene primer set 27F/1519R [17], and then the PCR product was re-amplified by nested PCR using the primer set PLA491F/OXY1313R. Purified PCR products were cloned into vector pCR®2.1-TOPO® and transformed into *E. coli* competent cells following the manufacturer's instructions (Invitrogen, Carlsbad, California). Clone inserts were amplified with the same primers as described above and purified. Partial sequences (about 750 bp) were determined from purified PCR products by using Big Dye Terminator V3.1 (Applied Biosystems, Foster city, CA, USA) run on an ABI prism 3100 sequencer (Applied Biosystems).

### Sequence analysis

Partial sequences were compared to those available in public databases with the NCBI BLAST web application (July 2009, Table S1). Partial sequences were clustered into distinct OTUs with Fastgroup II (http://biome.sdsu.edu/fastgroup/) with the sequence match parameter set at 80% corresponding roughly to a 98% sequence identity level and one representative sequence per OTUs was chosen for phylogenetic analysis (Tables S1 and S2). Representative sequences were aligned with related sequences from public databases using the slow and iterative refinement method FFT-NS-I with MAFFT [18] 5.8 software (http://align.bmr.kyushu-u.ac.jp/mafft/online/server/). Poorly aligned and very variable regions of the alignments were automatically removed with Gblocks [19] using the following parameters: allowing gap position equal to “with half”, minimum length of block equal to 5 for the general analysis. Different nested models of DNA substitution and associated parameters were estimated using Modeltest [20]. Each alignment was analyzed by Maximum Parsimony (MP), Neighbor Joining (NJ) and Maximum Likelihood (ML) using PAUP 4.0b10 [21]. A heuristic swapping algorithm was performed to find the optical ML tree topology (with 70,000 rearrangements). Bootstrap values for NJ and MP were estimated from 1,000 replicates.

Sequences used in the analysis (i.e. only those corresponding to eukaryotes) have been deposited in the GenBank database under accession numbers HM132934–HM133569.

## Results

### Phylogenetic diversity of photosynthetic picoeukaryotes in the South East Pacific Ocean

The genetic diversity of photosynthetic picoeukaryotes was assessed on a transect through the South East Pacific (Figure 1 and Table 1) based on 16 samples collected at 9 stations at the surface and/or at the deep chlorophyll maximum (DCM) during the BIOSOPE cruise in 2004 [14]. Photosynthetic picoeukaryotes were sorted by flow cytometry and the plastid 16S rRNA gene amplified, cloned, and partially sequenced. Among the 812 16S rRNA gene sequences, 705 sequences were affiliated to eukaryotes, the rest corresponding to bacteria. For most samples, two primer sets were used, OXY107F/OXY1313R targeting oxygenic micro-organisms including cyanobacteria and PLA491F/OXY1313R designed to preferentially amplify photosynthetic eukaryotes, yielding 273 and 432 partial-length plastid 16S rRNA gene fragments, respectively (Table 1). A small fraction of sequences belonged to Streptophyta and Rhodophyta, corresponding to plants and macroalgae, respectively, and were removed from further phylogenetic analysis. The remaining 636 plastid sequences (Table S1) could be grouped into 62 operational taxonomic units (OTUs) using a 80% FastGroup II sequence match level, corresponding to a 98% sequence similarity level (Table S2). The best-represented eukaryotic groups were Stramenopiles (53%), Haptophyta (26%), and Chlorophyta (11%).

**Figure 2:**
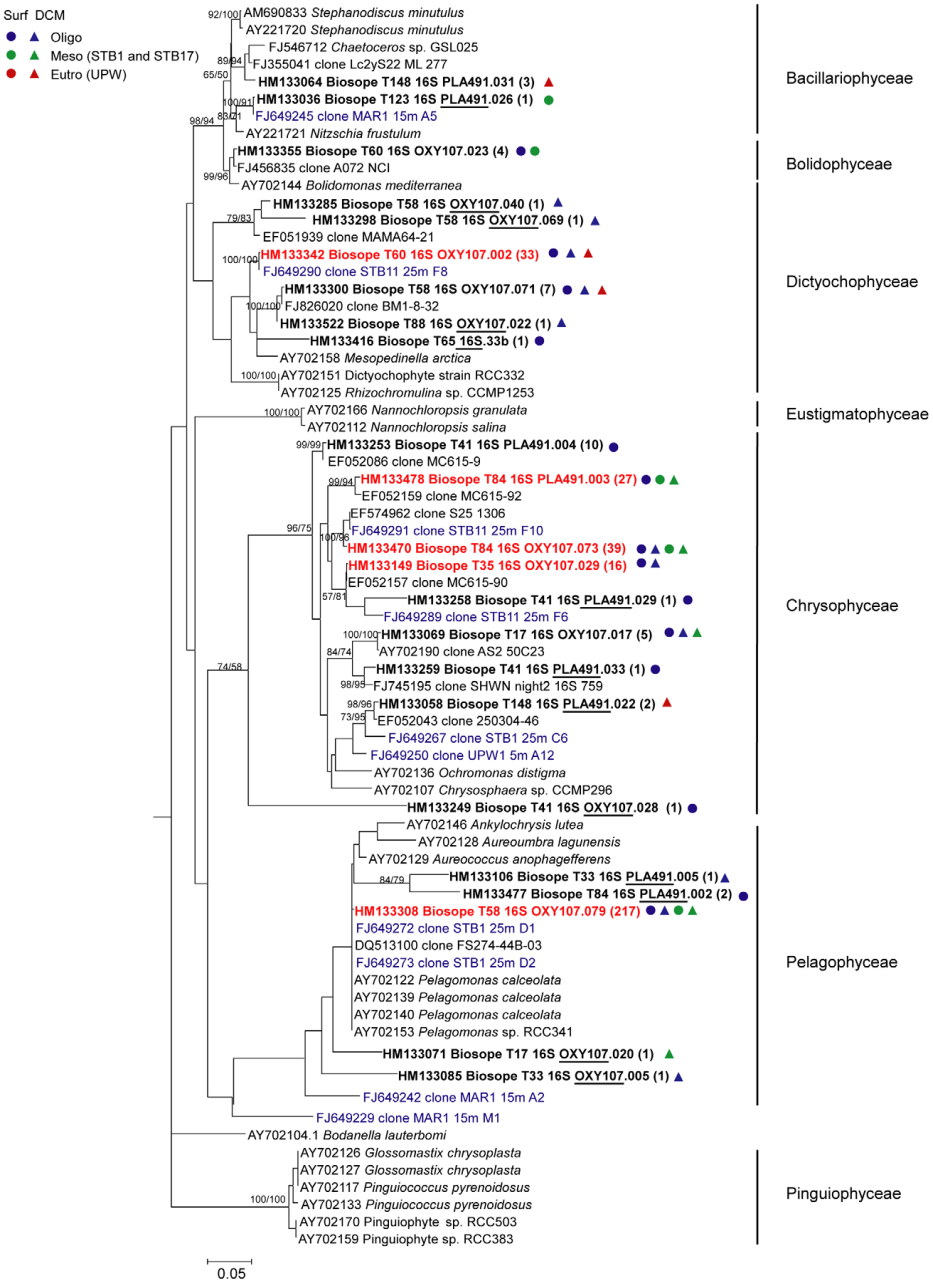
Phylogenetic tree of plastid 16S rRNA gene sequences for Stramenopiles. Sequences were retrieved from photosynthetic picoeukaryotes sorted by flow cytometry in the South East Pacific. The tree is inferred from 736 positions of an alignment of 72 partial sequences. The phylogenetic tree was constructed by the maximum likelihood (ML) method based on a TrN+I+G model of DNA substitution with a gamma distribution shape parameter of 0.6731 and substitution rates of R(b)[A–G] = 4.7980, R(e)[C–T] = 6.2129 and 1.0 for all other substitution rates. Total number of rearrangements tried was 70,499. Bootstrap values over 50% are indicated on the internal branches obtained from both NJ and MP methods. Sequences in bold are representative of OTUs amplified with primers OXY107F or PLA491F. OTUs amplified by only one primer set are marked by underlining the corresponding primer. Numbers enclosed between parentheses correspond to the number of clones retrieved for each OTU. OTUs encompassing relatively abundant clones are labelled in red. Symbols correspond to areas where clones were recovered for each OTU. Sequences from filtered samples [9] appear in blue.

Among Stramenopiles, BIOSOPE sequences were mainly distributed within Pelagophyceae, Chrysophyceae, and Dictyochophyceae (Figure 2). Pelagophyceae showed the highest relative abundance in the clone libraries, with 6 OTUs regrouping 222 sequences. Interestingly, 97% of Pelagophyceae sequences fell into a single OTU clearly corresponding to the species *Pelagomonas calceolata*, and were broadly observed in both mesotrophic and oligotrophic stations, both in surface waters and at the DCM. Chrysophyceae showed a broad diversity with 9 distinct OTUs comprising 102 sequences, coming mainly from mesotrophic and oligotrophic waters (Figure 2). Chrysophyceae OTUs recovered in this study appeared to be closely related to previous environmental sequences obtained from the Gulf of Naples [22] and Arabian Sea [8], as well as to BIOSOPE sequences obtained from filtered samples [9] (Figure 2). The nature of the organisms to which these sequences belong remains mysterious, since very few marine Chrysophyceae are known [23] and the lack of 16S rRNA gene sequences from plastids of cultured strains belonging to this class makes the identification of most of the OTUs difficult [9]. 44 sequences were affiliated to Dictyochophyceae: one OTU was closely related to a BIOSOPE sequence from a filtered sample [9], and five other OTUs grouped with environmental sequences from the Arabian Sea [8], Gulf of Naples [22], and cultured representatives (Figure 2). A small fraction of the plastid 16S rRNA sequences belonged to Bacillariophyceae and Bolidophyceae (Figure 2).

**Figure 3:**
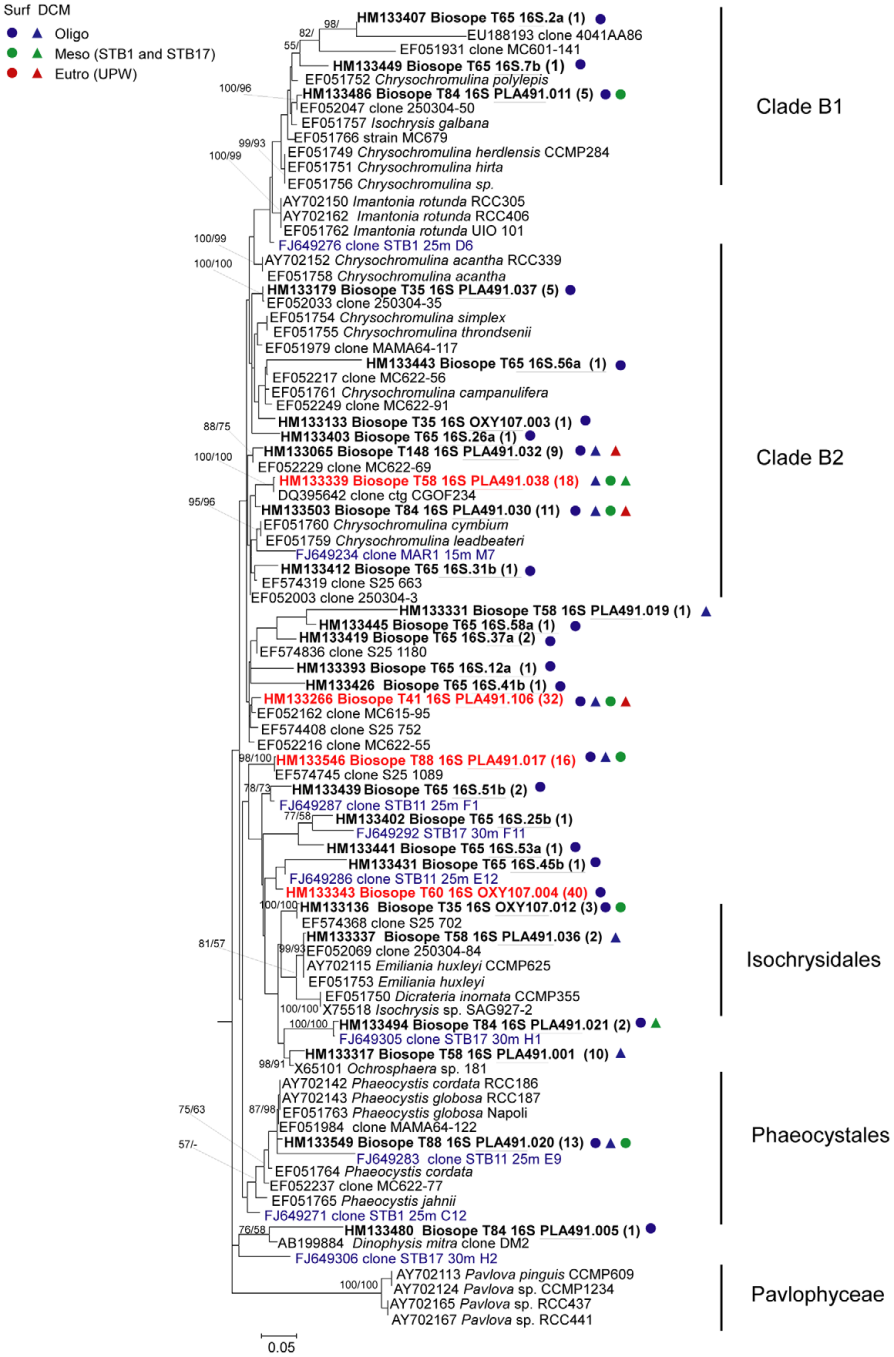
Phylogenetic tree of plastid 16S rRNA gene sequences for Haptophyta. The tree is inferred from 689 positions of an alignment of 89 partial sequences. See legend of Fig. 2 for details.

We obtained 29 distinct OTUs of Haptophyta, all of them affiliated with the Prymnesiophyceae, this class displaying the greatest diversity in the plastid 16S rRNA gene clone libraries (Figure 3). OTUs fell in all described orders/clades (Isochrysiales, Phaeocystales, Clades B1 and B2 [24]), a few being close to existing genera such as *Phaeocystis*, but most quite distant from any cultivated species that have been sequenced.

**Figure 4:**
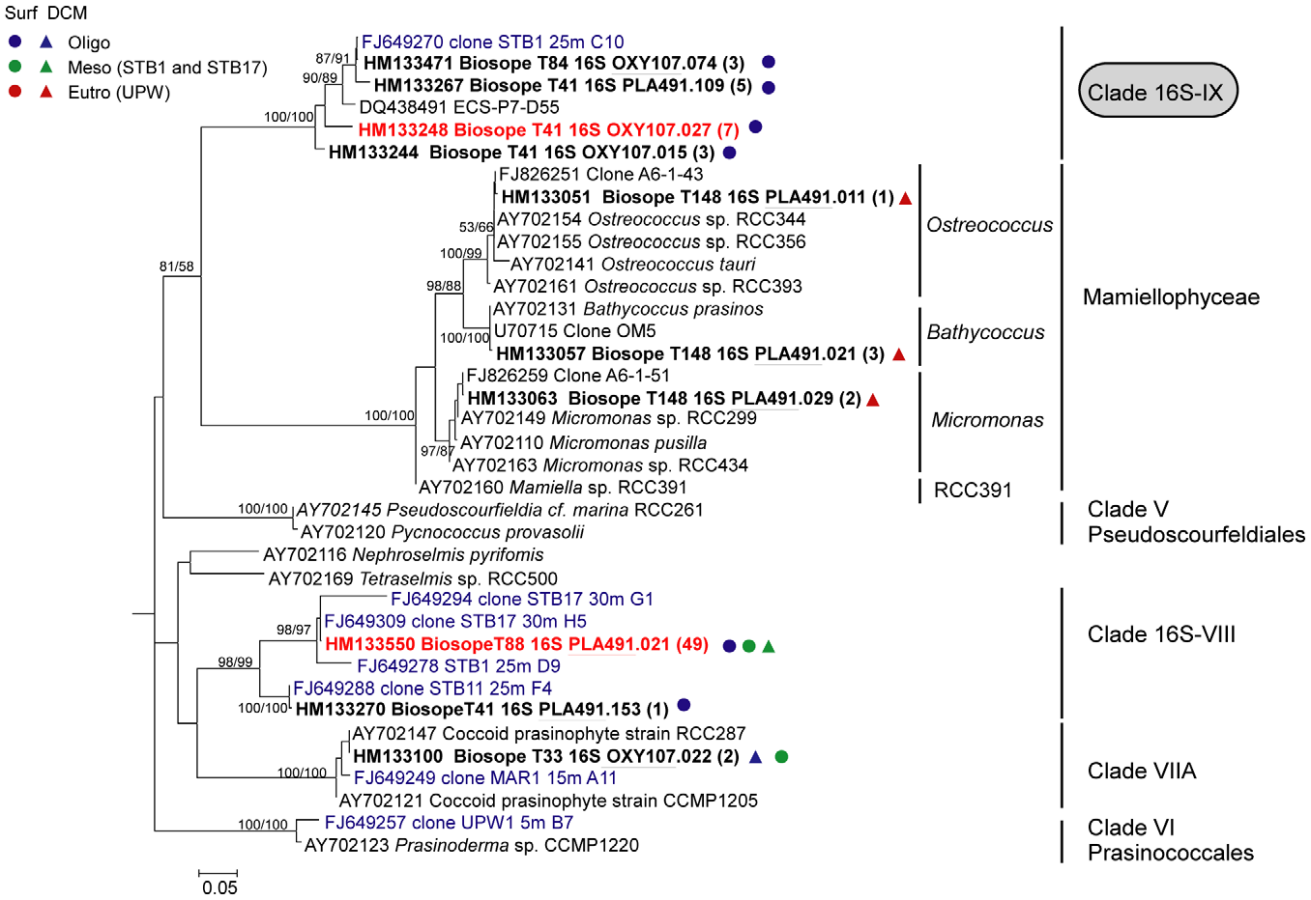
Phylogenetic tree of plastid 16S rRNA gene sequences for Chlorophyta. The tree is inferred from 710 positions of an alignment of 37 partial sequences. See legend of Fig. 2 for details. The novel prasinophyte clade 16S-IX is highlighted.

Chlorophyta sequences were divided into four lineages supported by high bootstrap values. Some OTUs were related to Prasinophyceae clade VIIA and to the Mamiellophyceae [25]. Six sequences were closely related to the three genera *Ostreoccocus*, *Bathycoccus*, and *Micromonas* (Figure 4). The rest of the OTUs had sequences with no close cultured counterparts and were assigned to two novel Prasinophyceae clades, 16S-VIII defined by Lepère *et al*. (2009) and 16S-IX defined here. Clade 16S-VIII was well represented in the clone libraries, but showed a low diversity, with a total of 50 sequences grouping into two OTUs (Figure 4). This clade also included four BIOSOPE sequences from 16S rRNA gene clone libraries from filtered samples. The novel clade 16S-IX (Figure 4) contained four OTUs from this study but also one sequence from the East China Sea and one from BIOSOPE filtered samples [9].

### Photosynthetic picoeukaryotes diversity as a function of primer sets

Clone libraries based on the 16S rRNA primer set OXY107F/OXY1313R, which is biased towards oxygenic phototrophs contained 21% bacterial sequences, against only 7% when using the primer set PLA491F/OXY1313R biased towards eukaryotic plastids. The use of OXY107F resulted in a high number of sequences related to proteobacteria and cyanobacteria, while PLA491F clone libraries contained a large number of Streptophyta sequences (data not shown). Excluding these bacteria and Streptophyta sequences, 10 OTUs were identified to be unique for OXY107F libraries, 38 OTUs unique for PLA491F libraries, and 14 OTUs were present in both libraries.

**Figure 5:**
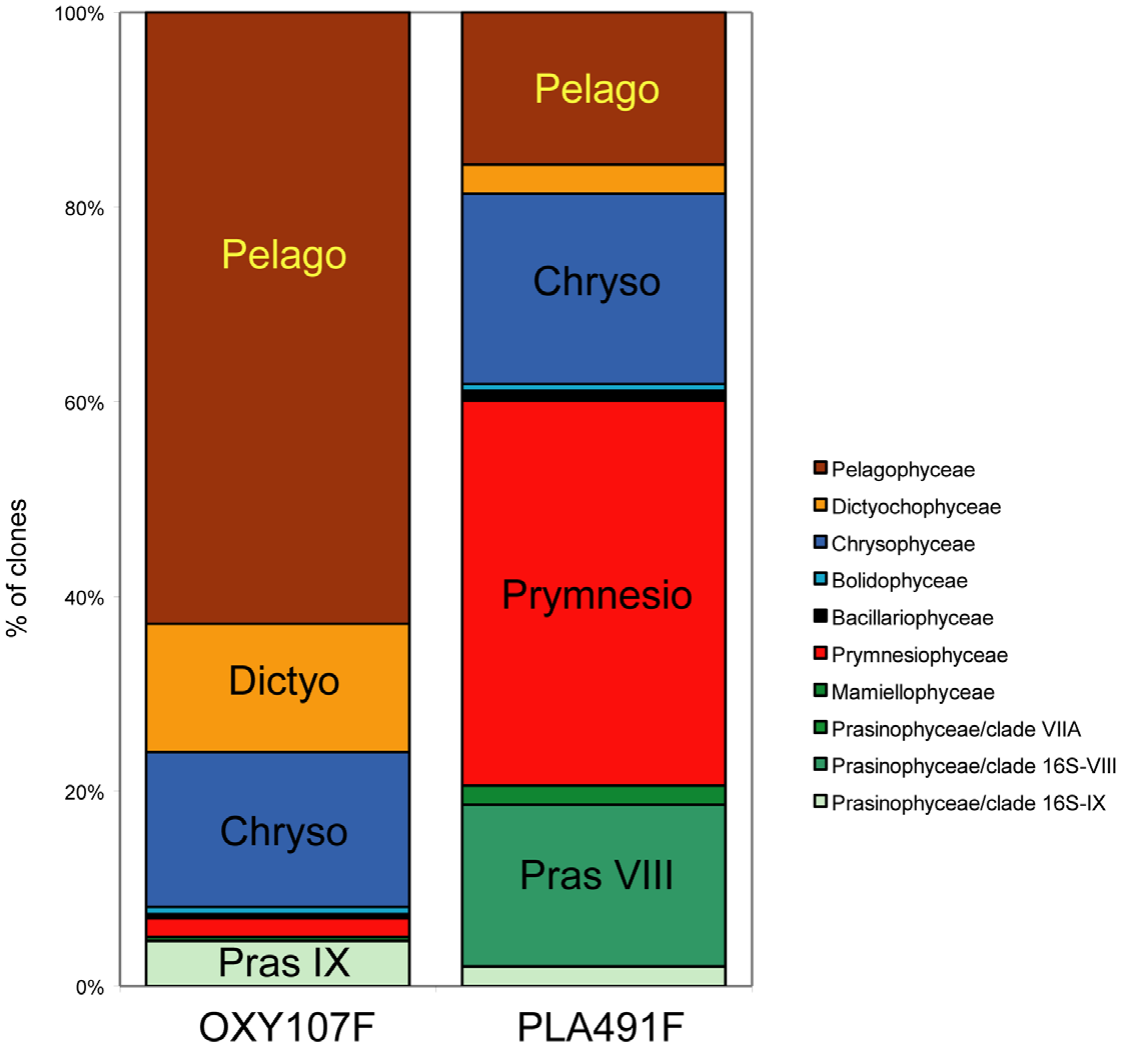
Overall composition of plastid 16S rRNA gene clone libraries constructed with two primer sets. Primer sets: OXY107F/OXY1313R and PLA491F/OXY1313R. Composition is expressed as percent of clones. Only samples for which both primers were used are included.

These results showed that there is a difference in the estimation of the composition of the picoplankton community depending on the primer set used. For example, Prymnesiophyceae sequences were very abundant when using the PLA491F/OXY1313R primer pair, accounting for 40% of sequences, but almost completely absent when using the OXY107F/OXY1313R primer set (Figures 3 and 5). Another discrepancy is that sequences of Prasinophyceae clade 16S-VIII and Mamiellophyceae were only retrieved by using primer PLA491F, whereas sequences of Prasinophyceae clade VIIA were only obtained by using primer OXY107F (Figures 4 and 5). Some taxa appeared in clone libraries constructed with both primer combinations, but their contribution was significantly different. For instance, Pelagophyceae represented 60% of the sequences in the clone libraries constructed with the OXY107F primer, but only contributed 17% of sequences in clone libraries constructed with primer PLA491F (Figure 5). Similarly, the percentage of Dictyochophyceae and Prasinophyceae clade 16S-IX in the photosynthetic picoeukaryote community was higher in the clone libraries constructed with primer OXY107F than with primer PLA491F (Figure 5). Chrysophyceae was the only taxonomic group present with almost equal frequency in the clone libraries constructed with either primer set (Figure 5).

### Distribution of photosynthetic picoeukaryote populations along the BIOSOPE transect

**Figure 6:**
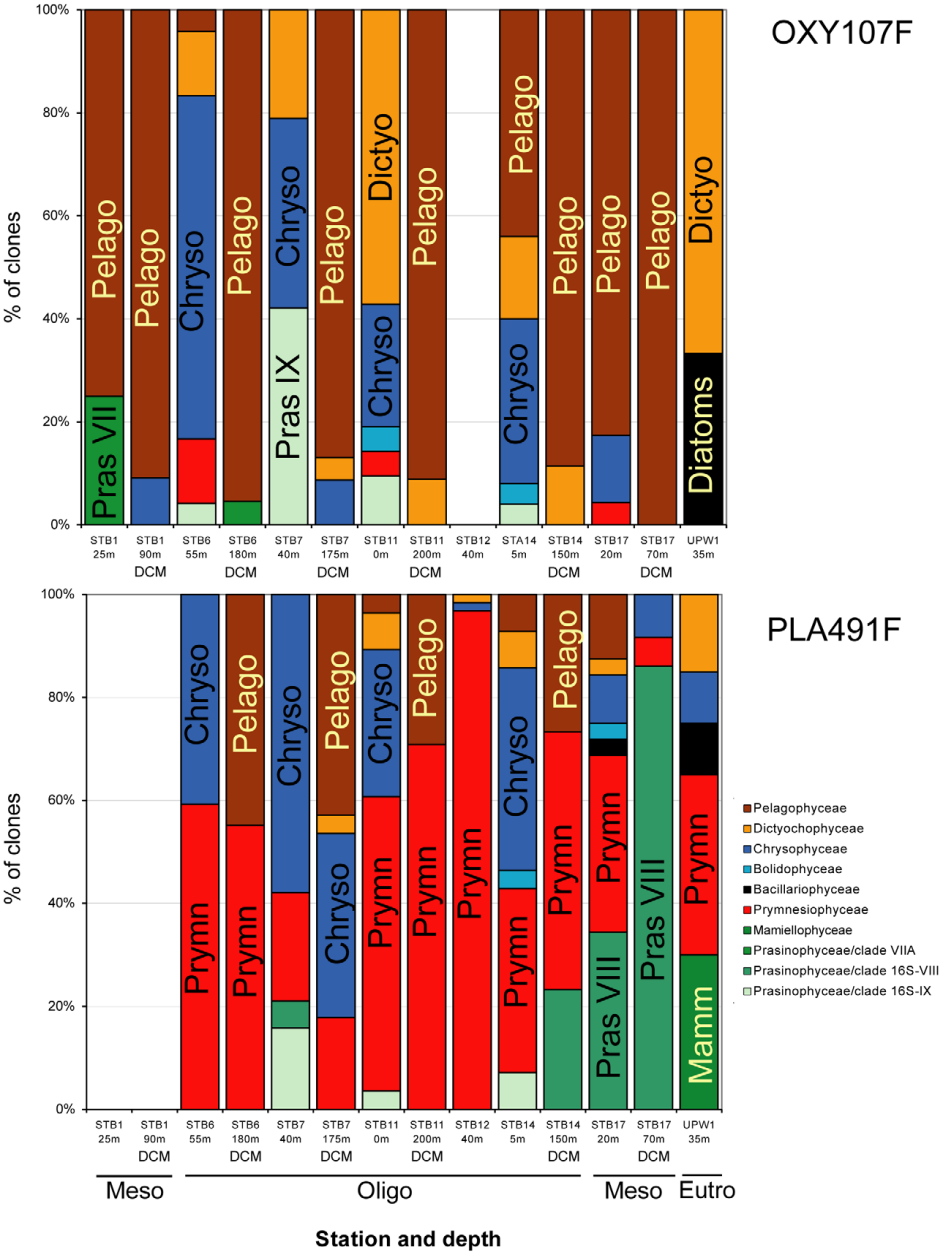
Detailed composition of plastid 16S rRNA gene clone libraries constructed with two primer sets. Primer sets: OXY107F/OXY1313R and PLA491F/OXY1313R. Composition is expressed as percent of clones. Oligo, Meso and Eutro correspond to the three major regions considered: oligotrophic, mesotrophic, and eutrophic.

Despite differences in the composition of the clone libraries constructed with the two different primer sets, there was some consensus in the distribution of photosynthetic picoeukaryote members along the BIOSOPE cruise transect. In oligotrophic regions (Stations 6 to 14, Table 1), Pelagophyceae, Chrysophyceae, Dictyophyceae and Prasinophyceae clade 16S-IX were widely present. Vertically, Pelagophyceae were mostly restricted to the DCM, while the latter 3 groups were prevalent in surface waters (Figure 6). The upwelling zone (UPW1) seemed to be characterised by Dictyochophyceae and Bacillariophyceae. Other features were only revealed by one primer set. The use of the PLA491F/OXY1313R primers revealed that Prymnesiophyceae broadly occurred both in surface waters and at the DCM. The novel Prasinophyceae clade 16S-VIII was abundant in the mesotrophic region (STB17). In addition, Mamiellophyceae sequences were only found in the upwelling region (UPW1, Figure 6). When the primer set OXY107F/OXY1313R was used, members of Prasinophyceae clade VIIA were observed in the mesotrophic region as well as at the boundary of mesotrophic and oligotrophic waters (STB1 and STB6, Figure 6).

## Discussion

### Methodological aspects

#### Comparison of the two primers OXY107F vs. PLA491F

It has been suggested that mismatches close to the 5′ end can result in poor amplification compared to a perfectly matching primer [26]. This is consistent with our results indicating that PLA491F and OXY107F do not equally amplify all phylogenetic groups in natural samples and that mismatches have a high influence on the efficiency of amplification. Primer OXY107F used in our study was originally designed to be specific for members of oxygenic phototrophs, i.e. cyanobacteria and eukaryotic plastids [15], [27]. It was however, reported to have significant mismatches to many algal sequences, especially Prasinophyceae and Chrysophyceae [8]. Based on sequences more recently available, we found that this primer has 3 mismatches with some Prasinophyceae and Mamiellophyceae, as well as 4 mismatches with Prymnesiophyceae (Figure S1). Indeed Prasinophyceae and Prymnesiophyceae were almost completely missing from clone libraries using this primer, probably due to the mismatches. The alternative primer PLA491F was designed and has been tested to amplify all known algal plastids based on sequences from cultures [8], although it has one or more mismatches at the 5′ end with several algal classes (Figure S1). When applied to environmental samples, our data indicate that PLA491F preferentially amplifies Prymnesiophyceae, Chrysophyceae, and Mamiellophyceae, which match perfectly this primer (Figure S1), but did not show any (or very low) amplification for Prasinophyceae clade VIIA and Dictyochophyceae. Cultured Dictyochophyceae sequences have one mismatch to primer PLA491F (Figure S1), but unfortunately no sequences from clade VIIA are available in the primer region. However, sequence comparison alone is not always a reliable indicator for predicting the outcome of PCR [28]. For example, despite a mismatch at the third position (5′ end) with Pelagophyceae (Figure S1), the use of primer PLA491F resulted in a large number of Pelagophyceae sequences, probably because of the large abundance of Pelagophyceae in this region as revealed by Primer OXY107F but also from cultures since *Pelagomonas calceolata* was the most commonly isolated species in this region [29].

#### Comparison of sorted samples vs. filtered samples

BLAST analysis showed that among the 636 plastid sequences that we retrieved from sorted BIOSOPE picoeukaryote samples, 281 have the closest similarity (Table S1) with eukaryotic plastid 16S rRNA gene sequences from BIOSOPE samples filtered through 3 µm [9]. This implies that clone libraries obtained with these two different approaches results in a large overlap of sequences.

We carried out a comparison of clone libraries using primer set PLA491F/OXY1313R, for sorted samples and filtered samples at three BIOSOPE stations including oligotrophic, mesotrophic and eutrophic waters (STB11, STB17 and UPW1, respectively; Table 2) for which both types of samples were analyzed. In oligotrophic waters (STB11), Prymnesiophyceae, Chrysophyceae, and Dictyochophyceae were found with both approaches, while Pelagophyceae and Prasinophyceae clade 16S-IX were only found in sorted samples and Prasinophyceae clade 16S-VIII only in filtered samples. In mesotrophic waters (STB17), the clone library constructed from sorted cells appeared to be more diverse than the one constructed from filtered cells. Prymnesiophyceae and Prasinophyceae clade 16S-VIII were observed in filtered samples, but five other phylotypes were discovered in the clone library from sorted samples. In the upwelling region (UPW1), Prymnesiophyceae, Chrysophyceae, and Bacillariophyceae were retrieved in clone libraries from both sorted and filtered samples. Dictyochophyceae and Mamiellophyceae were only found in the sorted sample, while Cryptophyta, Prasinophyceae clade VI and clade 16S-VIII were present in the filtered sample. The lack of Cryptophyta in the sorted samples is explained by the fact that Cryptophyta, that display both red chlorophyll and orange phycoerythrin fluorescences, were not sorted with the settings used during the cruise. Qualitative and quantitative studies have demonstrated that Mamiellophyceae is a dominant group in coastal areas [12], [30], [31]. However, this class was surprisingly absent from the clone library constructed from the filtered sample from upwelling station UPW1, while it accounted for 46% of the clone library from the sorted sample. This confirms data obtained on 18S rRNA clone libraries [13] demonstrating that sorted samples may provide a better image of the natural community. This could be due to the lower diversity of templates present in sorted compared to filtered samples, since the latter include both prokaryotes and eukaryotes and both autotrophic and heterotrophic populations [13]. Therefore, some rare templates may be amplified in sorted samples and not in filtered samples, because template competition is lower in the former samples.

**Table 2:** Comparison of photosynthetic picoeukaryote community structure revealed from sorted samples (this study) *vs*. filtered samples [9], using primer set PLA491F/OXY1313R.

		STB11# of clones(# of sequences)		STB17# of clones(# of sequences)		UPW1# of clones(# of sequences)	
Division	Class	sorted	filtered	sorted	filtered	sorted	filtered
Haptophyta	Prymnesiophyceae	16	63 (4)	11	102 (14)	7	33 (3)
Stramenopile	Pelagophyceae	1		4			
	Chrysophyceae	8	10 (2)	3		2	1 (1)
	Dictyochophyceae	2	6 (1)	1		3	
	Bolidophyceae			1			
	Bacillariophyceae			1		2	63 (2)
Chlorophyta	Mamiellophyceae					6	
	Prasinophyceae Clade VI						5 (4)
	Prasinophyceae Clade 16S-VIII		1 (1)	11	15 (6)		13 (5)
	Prasinophyceae Clade 16S-IX	1					
Cryptophyta	Cryptophyceae						1

For sorted samples all clones were sequenced while for filtered samples [9], clones were first screened by RFLP (Restriction Fragment Length Polymorphism) and only a few clones were sequenced for each distinct RFLP pattern. Both the number of clones and sequences (between parentheses) are indicated.

#### Comparison of community structure based on plastid 16S vs. nuclear 18S rRNA gene

Two main approaches have been used to analyze photosynthetic eukaryote diversity: analysis of the nuclear 18S rRNA gene [5] and analysis of the plastid 16S rRNA gene [8]. However, to our knowledge, no comparison has been performed on the same sample set. Such a comparison on filtered samples is not very meaningful since 18S rRNA gene clone libraries are always dominated by heterotrophic groups that mask the real extent of diversity of photosynthetic eukaryotes [7]. In contrast this comparison is much more interesting on sorted samples that should contain only photosynthetic cells. In the BIOSOPE sorted samples (this study and [12]), these two markers reveal similar general trends for some taxonomic groups, such as the Chrysophyceae, which are clearly a key component of the community in surface waters of the central South East Pacific gyre, Prymnesiophyceae that widely occurred in oligotrophic regions, and Mamiellophyceae that were dominant in the upwelling zone. Still, some discrepancies are also apparent. Pelagophyceae and Dictyochophyceae were abundant in 16S rRNA gene libraries, but only marginally present in 18S rRNA gene libraries. Similarly, 18S rRNA gene clone libraries pointed out the importance of Chlorophyta along the whole BIOSOPE transect: Prasinophyceae clade IX in oligotrophic waters, Prasinophyceae clade VII in mesotrophic regions, and Mamiellophyceae in the upwelling [12]. While 16S rRNA libraries also suggest the presence of Chlorophyta in the South East Pacific Ocean, with Prasinophyceae clade 16S-IX in the hyper-oligotrophic ocean, Prasinophyceae clade VII and a novel clade 16S-VIII in mesotrophic waters, as well as Mamiellophyceae in upwelled coastal waters, their contribution appeared much more limited than in 18S rRNA gene clone libraries.

Nuclear rDNA PCR-based studies of eukaryotic communities are subject to selective amplification biases due to GC content [30]. Our data suggest that the GC content of the plastid 16S rRNA gene is similar to that of the 18S rRNA gene in the sorted samples, with the exception of Chrysophyceae for which the 18S rRNA gene has a low %GC (Table 3). Therefore, %GC content should not cause any bias in clone library composition between the 16S rRNA and 18S rRNA genes. Gene copy number is another factor that must be taken into account when considering the bias of clone libraries [31]. In general plastid genomes only harbor two copies of the rRNA operon [e.g. 33], but plastids may have many copies of the plastid genome [32] whose number is probably regulated by environmental factors.

**Table 3:** %GC content (Mean and SD) of the partial plastid 16S rRNA gene sequences for the different phylogenetic groups recovered.

Division	Class	Order	N	%GC Mean16S rRNA	%GC SD16S rRNA	%GC Mean18S rRNA
Haptophyta	Prymnesiophyceae		29	48.1	1.0	49.8
Chlorophyta	Prasinophyceae	Clade 16S-IX	4	46.8	0.8	
		Clade 16S-VIII	2	51.7	1.9	
		Clade VIIA	1	48.7		49.1
	Mamiellophyceae	Mamiellales	3	47.5	0.8	47.6
Stramenopiles	Bacillariophyceae		2	47.5	0.9	
	Bolidophyceae		1	46.8		
	Dictyochophyceae		3	47.8	0.1	
	Chrysophyceae		9	46.6	0.8	42.5
	Pelagophyceae		6	47.8	0.9	48.0

For comparison, the 18S rRNA gene %GC values are reprinted from Shi *et al.* [12].

### Photosynthetic picoeukaryote community structure in the South East Pacific

**Figure 7:**
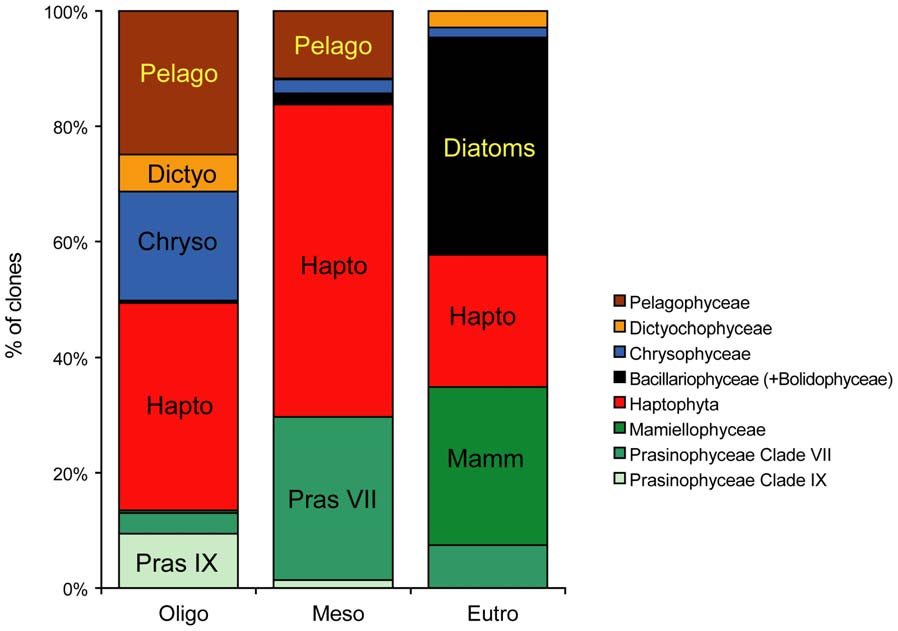
Overall composition of the photosynthetic picoeukaryote community for the three major regions considered. Composition is expressed as percent of clones. This synthetic figure is based on a compilation of the four approaches used: 16S plastid primer sets OXY107F/OXY1313R and PLA491F/OXY1313R on sorted material (this study), 16S plastid primer set PLA491F/OXY1313R on filtered material [9] and 18S nuclear primers on sorted material [12]. Oligo, Meso and Eutro correspond to the three major regions considered: oligotrophic, mesotrophic, and eutrophic. Stations included in the three regions are detailed in Table 1. Station MAR1 (off the Marquesas Islands), which was only analyzed in reference [9] has been included in the mesotrophic pool.

As detailed above, each of the cloning strategies used: plastid 16S rRNA genes amplified from filtered samples [9], nuclear 18S rRNA genes amplified from sorted samples [12], and plastid 16S rRNA genes amplified from sorted samples (this work), provides a different image of the photosynthetic picoeukaryote community in the South East Pacific Ocean, a particularly interesting area which encompasses highly contrasted trophic regimes, including the most oligotrophic water in the world ocean [14]. These different images can be brought together to establish some key features of this region (Figure 7).

Despite the high concentration of their diagnostic pigment 19′hexanoyloxyfucoxanthin in many oceanic waters [30], Prymnesiophyceae are often underestimated in picoeukaryote clone libraries constructed from filtered samples using general nuclear 18S rRNA gene primers [33]. However this bias seems to disappear when starting from sorted populations or when using plastid primers. Both 16S rRNA and 18S rRNA gene sequences showed that Prymnesiophyceae display a great diversity in this region. A small number of OTUs from 16S rRNA genes fell into previously described orders/clades (Isochrysiales, Phaeocystales, clades B1 and B2), but most were quite distant from any known lineages without any clear affiliation. Prymnesiophyceae sequences derived from the 18S rRNA gene corresponded to the same orders/clades (Isochrysiales, Phaeocystales, and clade B2), with just one clade missing (clade B1). However, besides those clades, 18S rRNA gene sequences were also affiliated to other described orders/clades (Braarudosphaeraceae and Coccolithales, clades E and D). A novel class, besides the Prymnesiophyceae and Pavlovophyceae was also discovered for the 18S rRNA gene [12]. Compared with the 18S rRNA gene, the affiliations of the Prymnesiophyceae 16S rRNA gene sequences were less robust, as indicated by the low bootstrap values, likely due to a less comprehensive database for the plastid 16S rRNA gene. The massive diversity of Prymnesiophyceae indicated by 18S rRNA and 16S rRNA gene sequences is consistent with the results of Liu *et al*. [30] based on LSU rRNA gene clone libraries using haptophyte-specific primers. More recently the use of 18S rRNA probes detected by FISH have revealed that small Prymnesiophyceae (2–3 µm in size) are abundant in the sub-tropical and tropical North-East Atlantic [2], [3].

Among stramenopiles, 16S rRNA gene data demonstrate that Chrysophyceae are widely distributed in the SE Pacific Ocean, particularly in surface waters, which is consistent with 18S rRNA gene data in the same region [12] as well as plastid 16S rRNA gene data in the Arabian Sea [8]. However photosynthetic Chrysophyceae are generally regarded as restricted to freshwater environments and very few species have been described from the marine environment (e.g. *Ochromonas*). Most marine Chrysophyceae are heterotrophic (e.g. *Paraphysomonas*) but these lack plastids and the plastid 16S rRNA gene cannot be amplified from them [8].

Pelagophyceae were another abundant stramenopile class in the present study. It was retrieved by both primer sets and was widely distributed at all oceanic stations particularly at the DCM but was absent from the upwelling region. This is consistent with the 18S rRNA gene data [12] and with the isolation of large number of Pelagophyceae cultures [29] from the same region. Almost all cultures had 18S rRNA gene sequences similar to *P. calceolata*, a photosynthetic flagellate which was initially isolated from the North Pacific central gyre [34]. In the present study, 217 plastid 16S rRNA gene sequences grouped into one OTU that corresponds to *P. calceolata* (Figure 2), which confirms that this species is probably the most important pelagophyte in oceanic waters.

In contrast to the paucity of Dictyochophyceae that was found in our previous studies in this region [9], [12], this group turned out to be widely distributed in plastid sequences from sorted samples. One Dictyochophyceae OTU, encompassing a large number of sequences, was found in both oligotrophic and upwelling regions. These sequences do not fall close to those from cultured strains, but the number of sequences from existing dictyochophyte cultures is very low. In fact, the new environmental sequences may correspond to the order Florenciellales, for which a novel species has been isolated during the BIOSOPE cruise [29].

Bacillariophyceae (diatoms) mainly occurred in waters with relative high nutrient concentrations (e.g. station UPX1). This agrees with pigment analyses that found significant concentrations of fucoxanthin, a tracer of diatoms, in the upwelling zone [35] as well as microscopic observations [36].

16S rRNA gene clone libraries revealed fewer sequences of Prasinophyceae than 18S rRNA gene clone libraries. Prasinophyceae clade 16S-VIII, which lacks any cultured representative, was initially found in clone libraries constructed from filtered samples [9] and was speculated to correspond to clade 18S-IX discovered by Viprey *et al*. [11] in the Mediterranean Sea and found in the South East Pacific by Shi *et al.* [12]. However, the present study that relies on more samples than that of Lepère *et al.* [9], reveals that the majority of 16S-VIII sequences are mainly present in mesotrophic waters, with only a few of them occurring close to oligotrophic waters. Moreover the phylogenetic position of clade 16S-VIII (Figure 4) leads us to hypothesize that clade 16S-VIII may correspond to sub-clade 18S-VIIB. Currently, no plastid sequence is available for this sub-clade although a few strains are available, including one isolated during BIOSOPE [29]. In support of this hypothesis, in all sorted samples where we detected 16S-VIII sequences (this work), the 18S-VIIB clade was also present [12]. The new Prasinophyceae clade 16S-IX, also without cultured representatives, is restricted to hyper-oligotrophic waters. This suggests that it may correspond to clade 18S-IX since the latter is characteristic of very oligotrophic waters from the Eastern Mediterranean Sea [11] and South East Pacific Ocean [12]. In fact, in all sorted samples where clade 16S-IX was observed, clade 18S-IX was also present [12]. Prasinophyceae clade VIIA occurred in relatively mesotrophic waters, which is consistent with isolation studies [29] and 18S rRNA gene clone library results [12]. More generally, Prasinophyceae clade VII appears characteristic of mesotrophic and mildly oligotrophic waters since sequences were also retrieved from waters with similar trophic status in the equatorial Pacific Ocean [5] and in the western Mediterranean Sea [11]. Finally, Mamiellophyceae, including *Ostreococcus*, *Bathycoccus* and *Micromonas*, were only detected in upwelling coastal waters. This is consistent with the distribution [35] of characteristic pigment (prasinoxanthin and chlorophyll *b*) and 18S rRNA gene data along the transect [12].

### Conclusion

This study confirms that flow cytometry sorting is an efficient way of separating photosynthetic populations from the rest of the microbial population in order to better characterize their diversity. The use of various approaches (different primers and/or genes) resulted in similarities as well as differences. Therefore, caution should be exercised when interpreting the genetic diversity of a given community based on a single approach. Five main groups emerge as key contributors to small photosynthetic eukaryotes in oceanic waters: Chlorophyta, Chrysophyceae (mostly in surface), Pelagophyceae (mostly at the DCM), Dictyochophyceae and Haptophyta (Figure 7). However the vast majority of the clades containing photosynthetic sequences remain uncultivated emphasizing the need for a strong isolation effort.

## Supporting Information

Figure S1 Specificity of the primers OXY107F and PLA491F, illustrated by alignment of the primers against algal plastid and cyanobacterial 16S rRNA gene sequences. Note that there are more sequences for PLA491F because this region is better covered by publicly available sequences.
(PDF)

Table S1 Plastid 16S rRNA gene sequences obtained from BIOSOPE sorted samples (Bacteria excluded). OTU assignment is based on FastGroup II results with 80% similarity. Taxonomic assignments have been made based on the information from BLAST and from an annotated database.
(PDF)

Table S2 Plastid 16S rRNA gene sequences representative of OTUs obtained from BIOSOPE sorted samples (Bacteria excluded).
(PDF)
