# Supplementary material for: Plastid 16S rRNA Gene Diversity among Eukaryotic Picophytoplankton Sorted by Flow Cytometry from the South Pacific Ocean
Source: PLoS One. 2011 Apr 28;6(4):e18979. doi: 10.1371/journal.pone.0018979 (PMC3084246; doi:10.1371/journal.pone.0018979)
Supplement: Figure S1 — Specificity of the primers OXY107F and PLA491F, illustrated by alignment of the primers against algal plastid and cyanobacterial 16S rRNA gene sequences. Note that there are more sequences for PLA491F because this region is better covered by publicly available sequences. (PDF) [file pone.0018979.s001.pdf]

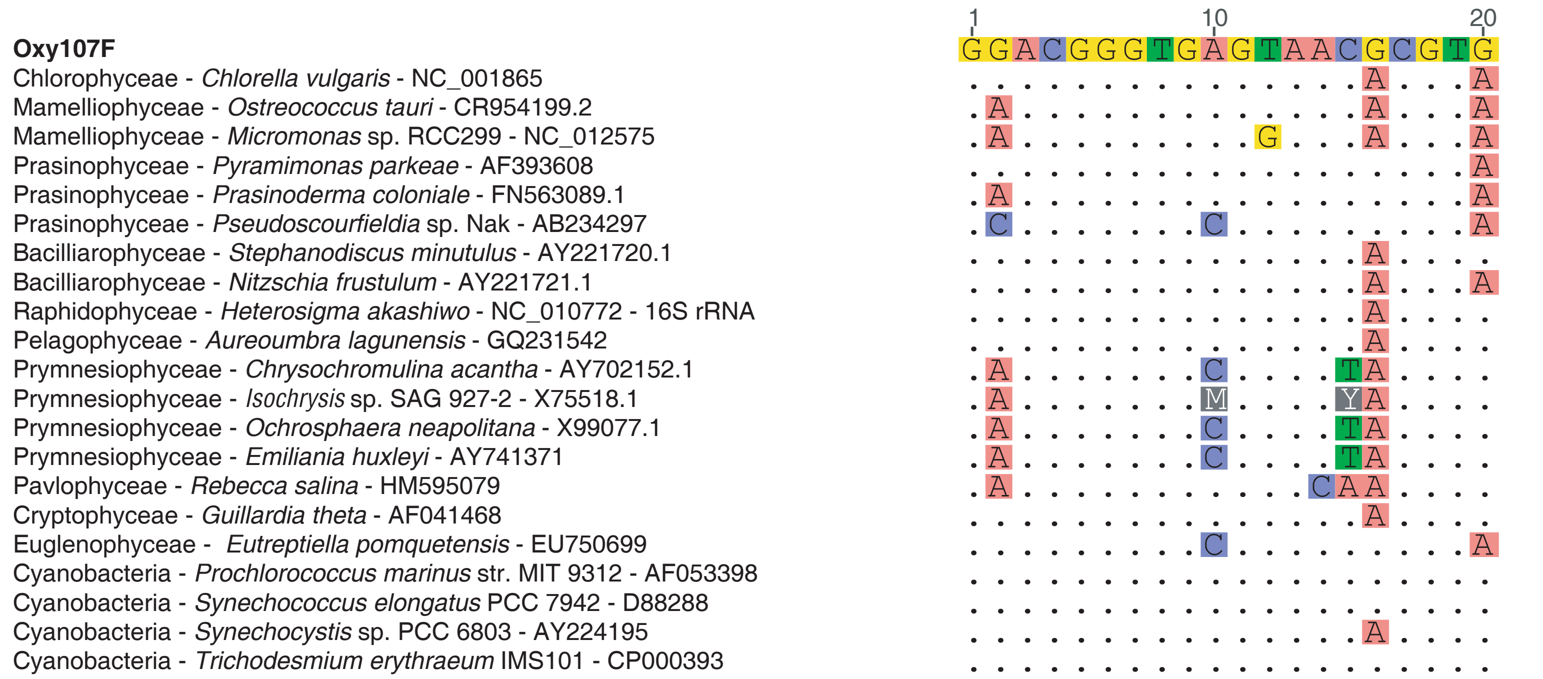
1
10
20
Oxy107F
GGACGGGTGAGTAACGCGTG
Chlorophyceae - Chlorella vulgaris - NC_001865
Mamelliophyceae - Ostreococcus tauri - CR954199.2
Mamelliophyceae - Micromonas sp. RCC299 - NC_012575
Prasinophyceae - Pyramimonas parkeae - AF393608
Prasinophyceae - Prasinoderma coloniale - FN563089.1
Prasinophyceae - Pseudoscourfieldia sp. Nak - AB234297
Bacilliarophyceae - Stephanodiscus minutulus - AY221720.1
Bacilliarophyceae - Nitzschia frustulum - AY221721.1
Raphidophyceae - Heterosigma akashiwo - NC_010772 - 16S rRNA
Pelagophyceae - Aureoumbra lagunensis - GQ231542
Prymnesiophyceae - Chrysochromulina acantha - AY702152.1
Prymnesiophyceae - Isochrysis sp. SAG 927-2 - X75518.1
Prymnesiophyceae - Ochrosphaera neapolitana - X99077.1
Prymnesiophyceae - Emiliania huxleyi - AY741371
Pavlophyceae - Rebecca salina - HM595079
Cryptophyceae - Guillardia theta - AF041468
Euglenophyceae - Eutreptiella pomquetensis - EU750699
Cyanobacteria - Prochlorococcus marinus str. MIT 9312 - AF053398
Cyanobacteria - Synechococcus elongatus PCC 7942 - D88288
Cyanobacteria - Synechocystis sp. PCC 6803 - AY224195
Cyanobacteria - Trichodesmium erythraeum IMS101 - CP000393

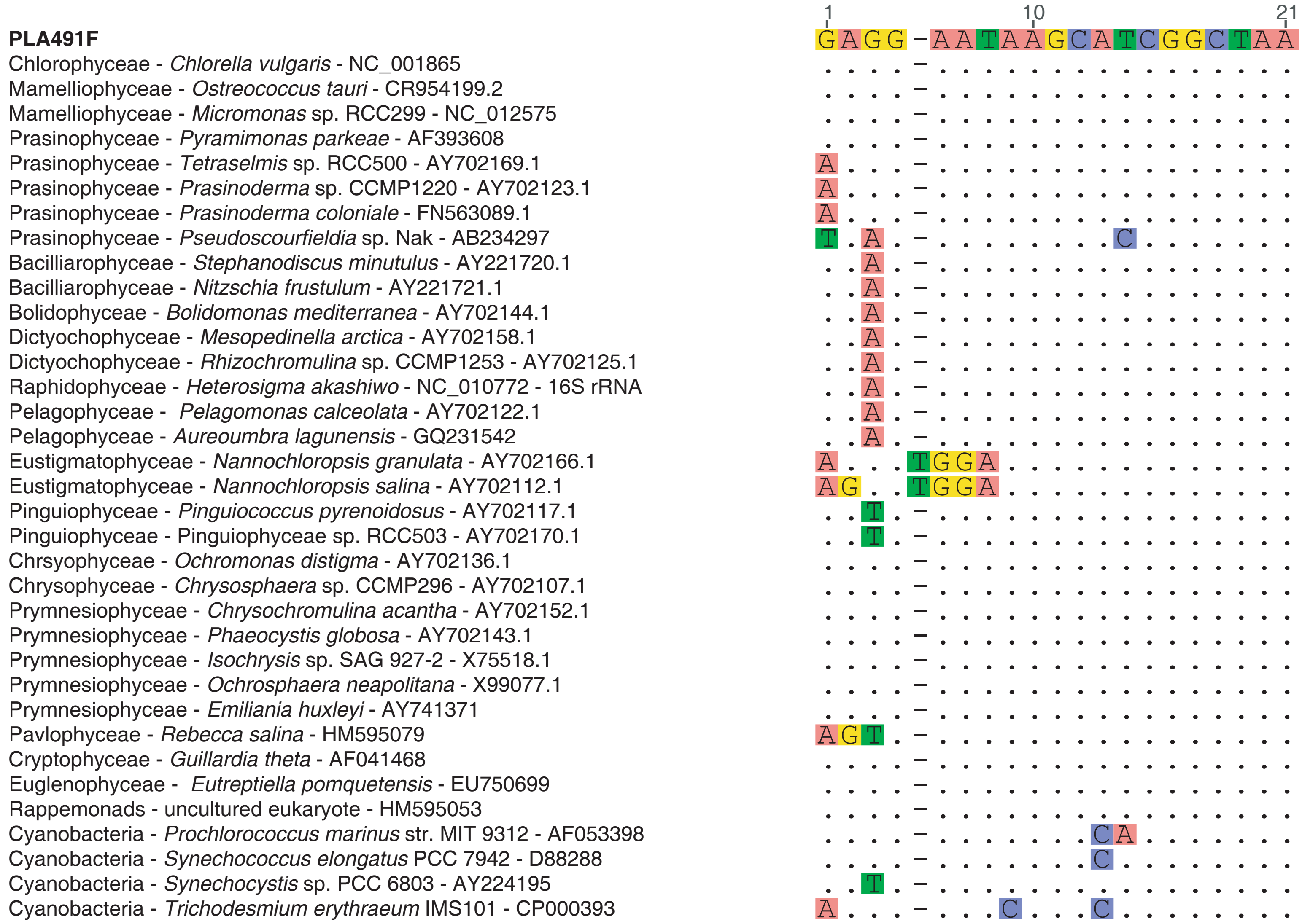
1
10
21
PLA491F
GAGG–AATAAGCATCGGCTAA
Chlorophyceae - Chlorella vulgaris - NC_001865
Mamelliophyceae - Ostreococcus tauri - CR954199.2
Mamelliophyceae - Micromonas sp. RCC299 - NC_012575
Prasinophyceae - Pyramimonas parkeae - AF393608
Prasinophyceae - Tetraselmis sp. RCC500 - AY702169.1
Prasinophyceae - Prasinoderma sp. CCMP1220 - AY702123.1
Prasinophyceae - Prasinoderma coloniale - FN563089.1
Prasinophyceae - Pseudoscourfieldia sp. Nak - AB234297
Bacilliarophyceae - Stephanodiscus minutulus - AY221720.1
Bacilliarophyceae - Nitzschia frustulum - AY221721.1
Bolidophyceae - Bolidomonas mediterranea - AY702144.1
Dictyochophyceae - Mesopedinella arctica - AY702158.1
Dictyochophyceae - Rhizochromulina sp. CCMP1253 - AY702125.1
Raphidophyceae - Heterosigma akashiwo - NC_010772 - 16S rRNA
Pelagophyceae - Pelagomonas calceolata - AY702122.1
Pelagophyceae - Aureoumbra lagunensis - GQ231542
Eustigmatophyceae - Nannochloropsis granulata - AY702166.1
Eustigmatophyceae - Nannochloropsis salina - AY702112.1
Pinguiophyceae - Pinguiococcus pyrenoidosus - AY702117.1
Pinguiophyceae - Pinguiophyceae sp. RCC503 - AY702170.1
Chrsyophyceae - Ochromonas distigma - AY702136.1
Chrysophyceae - Chrysosphaera sp. CCMP296 - AY702107.1
Prymnesiophyceae - Chrysochromulina acantha - AY702152.1
Prymnesiophyceae - Phaeocystis globosa - AY702143.1
Prymnesiophyceae - Isochrysis sp. SAG 927-2 - X75518.1
Prymnesiophyceae - Ochrosphaera neapolitana - X99077.1
Prymnesiophyceae - Emiliania huxleyi - AY741371
Pavlophyceae - Rebecca salina - HM595079
Cryptophyceae - Guillardia theta - AF041468
Euglenophyceae - Eutreptiella pomquetensis - EU750699
Rappemonads - uncultured eukaryote - HM595053
Cyanobacteria - Prochlorococcus marinus str. MIT 9312 - AF053398
Cyanobacteria - Synechococcus elongatus PCC 7942 - D88288
Cyanobacteria - Synechocystis sp. PCC 6803 - AY224195
Cyanobacteria - Trichodesmium erythraeum IMS101 - CP000393
