# Supplementary material for: Plastid 16S rRNA Gene Diversity among Eukaryotic Picophytoplankton Sorted by Flow Cytometry from the South Pacific Ocean
Source: PLoS One. 2011 Apr 28;6(4):e18979. doi: 10.1371/journal.pone.0018979 (PMC3084246; doi:10.1371/journal.pone.0018979)
Supplement: Table S1 — Plastid 16S rRNA gene sequences obtained from BIOSOPE sorted samples (Bacteria excluded). OTU assignment is based on FastGroup II results with 80% similarity. Taxonomic assignments have been made based on the information from BLAST and from an annotated database. (PDF) [file pone.0018979.s002.pdf]

Accession	station	CTD cast	depth	primer_forward	Clone_library	Clone	Division	Class	Order	Genus	OTU	BLAST Accession	BLAST % ID	BLAST Description
HM133077	STB1	53	25	OXY107	Biosope_T19_16S_OXY107	4	Stramenopiles	Pelagophyceae			HM133308	FJ649273	98.27%	Uncultured phototrophic eukaryote clone STB1_25m_D2 16S ribosomal RNA gene,partial sequence; chloroplast.
HM133078	STB1	53	25	OXY107	Biosope_T19_16S_OXY107	11	Stramenopiles	Pelagophyceae			HM133308	DQ513100	99.87%	Uncultured bacterium clone FS274-44B-03 16S ribosomal RNA gene, partialsequence.
HM133079	STB1	53	25	OXY107	Biosope_T19_16S_OXY107	36	Stramenopiles	Pelagophyceae			HM133308	DQ513100	99.74%	Uncultured bacterium clone FS274-44B-03 16S ribosomal RNA gene, partialsequence.
HM133080	STB1	53	25	OXY107	Biosope_T19_16S_OXY107	43	Chlorophyta	Prasinophyceae	clade VII		HM133100	AY702147	99.04%	Coccoid prasinophyte sp. RCC287 16S ribosomal RNA gene, partial sequence;plastid.
HM133066	STB1	53	90	OXY107	Biosope_T17_16S_OXY107	6	Stramenopiles	Pelagophyceae			HM133308	FJ649273	100.00%	Uncultured phototrophic eukaryote clone STB1_25m_D2 16S ribosomal RNA gene,partial sequence; chloroplast.
HM133067	STB1	53	90	OXY107	Biosope_T17_16S_OXY107	13	Stramenopiles	Pelagophyceae			HM133308	DQ513100	100.00%	Uncultured bacterium clone FS274-44B-03 16S ribosomal RNA gene, partialsequence.
HM133068	STB1	53	90	OXY107	Biosope_T17_16S_OXY107	16	Stramenopiles	Pelagophyceae			HM133308	FJ649273	99.74%	Uncultured phototrophic eukaryote clone STB1_25m_D2 16S ribosomal RNA gene,partial sequence; chloroplast.
HM133069	STB1	53	90	OXY107	Biosope_T17_16S_OXY107	17	stramenopiles	Chrysophyceae			HM133069	AY702190	99.47%	Uncultured phototrophic eukaryote clone AS2_50C23 16S ribosomal RNA gene,partial sequence; plastid.
HM133070	STB1	53	90	OXY107	Biosope_T17_16S_OXY107	18	Stramenopiles	Pelagophyceae			HM133308	FJ649273	100.00%	Uncultured phototrophic eukaryote clone STB1_25m_D2 16S ribosomal RNA gene,partial sequence; chloroplast.
HM133071	STB1	53	90	OXY107	Biosope_T17_16S_OXY107	20	Stramenopiles	Pelagophyceae			HM133071	FJ649273	99.82%	Uncultured phototrophic eukaryote clone STB1_25m_D2 16S ribosomal RNA gene,partial sequence; chloroplast.
HM133072	STB1	53	90	OXY107	Biosope_T17_16S_OXY107	36	Stramenopiles	Pelagophyceae			HM133308	FJ649273	99.87%	Uncultured phototrophic eukaryote clone STB1_25m_D2 16S ribosomal RNA gene,partial sequence; chloroplast.
HM133073	STB1	53	90	OXY107	Biosope_T17_16S_OXY107	37	Stramenopiles	Pelagophyceae			HM133308	FJ649273	99.34%	Uncultured phototrophic eukaryote clone STB1_25m_D2 16S ribosomal RNA gene,partial sequence; chloroplast.
HM133074	STB1	53	90	OXY107	Biosope_T17_16S_OXY107	48	Stramenopiles	Pelagophyceae			HM133308	DQ513100	99.87%	Uncultured bacterium clone FS274-44B-03 16S ribosomal RNA gene, partialsequence.
HM133075	STB1	53	90	OXY107	Biosope_T17_16S_OXY107	53	Stramenopiles	Pelagophyceae			HM133308	FJ649273	99.74%	Uncultured phototrophic eukaryote clone STB1_25m_D2 16S ribosomal RNA gene,partial sequence; chloroplast.
HM133076	STB1	53	90	OXY107	Biosope_T17_16S_OXY107	54	Stramenopiles	Pelagophyceae			HM133308	DQ513100	99.48%	Uncultured bacterium clone FS274-44B-03 16S ribosomal RNA gene, partialsequence.
HM133132	STB6	72	55	OXY107	Biosope_T35_16S_OXY107	1	Stramenopiles	Dictyochophyceae			HM133342	FJ649290	98.67%	Uncultured phototrophic eukaryote clone STB1_25m_F8 16S ribosomal RNA gene,partial sequence; chloroplast.
HM133133	STB6	72	55	OXY107	Biosope_T35_16S_OXY107	3	Haptophyta	Prymnesiophyceae			HM133133	EF052217	96.72%	Uncultured haptophyte clone MC622-56 16S ribosomal RNA gene, partialsequence; plastid.
HM133134	STB6	72	55	OXY107	Biosope_T35_16S_OXY107	10	stramenopiles	Chrysophyceae			HM133470	EF574962	100.00%	Uncultured bacterium clone S25_1306 16S ribosomal RNA gene, partialsequence.
HM133135	STB6	72	55	OXY107	Biosope_T35_16S_OXY107	11	stramenopiles	Chrysophyceae			HM133149	EF052157	99.86%	Uncultured chrysophyte clone MC615-90 16S ribosomal RNA gene, partialsequence; plastid.
HM133136	STB6	72	55	OXY107	Biosope_T35_16S_OXY107	12	Haptophyta	Prymnesiophyceae			HM133136	EF574358	99.74%	Uncultured bacterium clone S25_702 16S ribosomal RNA gene, partialsequence.
HM133137	STB6	72	55	OXY107	Biosope_T35_16S_OXY107	13	stramenopiles	Chrysophyceae			HM133470	EF574962	99.87%	Uncultured bacterium clone S25_1306 16S ribosomal RNA gene, partialsequence.
HM133138	STB6	72	55	OXY107	Biosope_T35_16S_OXY107	14	stramenopiles	Chrysophyceae			HM133478	EF052159	99.01%	Uncultured chrysophyte clone MC615-92 16S ribosomal RNA gene, partialsequence; plastid.
HM133139	STB6	72	55	OXY107	Biosope_T35_16S_OXY107	15	Haptophyta	Prymnesiophyceae			HM133136	EF574358	99.74%	Uncultured bacterium clone S25_702 16S ribosomal RNA gene, partialsequence.
HM133140	STB6	72	55	OXY107	Biosope_T35_16S_OXY107	16	stramenopiles	Chrysophyceae			HM133253	EF052086	99.87%	Uncultured chrysophyte clone MC615-9 16S ribosomal RNA gene, partialsequence; plastid.
HM133141	STB6	72	55	OXY107	Biosope_T35_16S_OXY107	17	stramenopiles	Chrysophyceae			HM133470	EF052073	98.42%	Uncultured chrysophyte clone 250304-89 16S ribosomal RNA gene, partialsequence; plastid.
HM133142	STB6	72	55	OXY107	Biosope_T35_16S_OXY107	18	stramenopiles	Chrysophyceae			HM133470	EF574962	99.87%	Uncultured bacterium clone S25_1306 16S ribosomal RNA gene, partialsequence.
HM133143	STB6	72	55	OXY107	Biosope_T35_16S_OXY107	22	Chlorophyta	Prasinophyceae	clade 16S-IX		HM133248	DQ438491	93.46%	Uncultured bacterium clone ECS-P7-D55 16S ribosomal RNA gene, partialsequence.
HM133144	STB6	72	55	OXY107	Biosope_T35_16S_OXY107	23	stramenopiles	Chrysophyceae			HM133253	EF052086	99.87%	Uncultured chrysophyte clone MC615-9 16S ribosomal RNA gene, partialsequence; plastid.
HM133145	STB6	72	55	OXY107	Biosope_T35_16S_OXY107	24	stramenopiles	Chrysophyceae			HM133470	EF574962	99.34%	Uncultured bacterium clone S25_1306 16S ribosomal RNA gene, partialsequence.
HM133146	STB6	72	55	OXY107	Biosope_T35_16S_OXY107	25	Stramenopiles	Dictyochophyceae			HM133342	FJ649290	98.54%	Uncultured phototrophic eukaryote clone STB1_25m_F8 16S ribosomal RNA gene,partial sequence; chloroplast.
HM133147	STB6	72	55	OXY107	Biosope_T35_16S_OXY107	27	stramenopiles	Chrysophyceae			HM133478	EF052159	99.60%	Uncultured chrysophyte clone MC615-92 16S ribosomal RNA gene, partialsequence; plastid.
HM133148	STB6	72	55	OXY107	Biosope_T35_16S_OXY107	28	stramenopiles	Chrysophyceae			HM133470	EF052073	98.52%	Uncultured chrysophyte clone 250304-89 16S ribosomal RNA gene, partialsequence; plastid.
HM133149	STB6	72	55	OXY107	Biosope_T35_16S_OXY107	29	stramenopiles	Chrysophyceae			HM133149	EF052157	99.87%	Uncultured chrysophyte clone MC615-90 16S ribosomal RNA gene, partialsequence; plastid.
HM133150	STB6	72	55	OXY107	Biosope_T35_16S_OXY107	30	Stramenopiles	Pelagophyceae			HM133308	GQ231541	97.48%	Aureococcus anophagefferens strain CCMP 1984 chloroplast, complete genome.
HM133151	STB6	72	55	OXY107	Biosope_T35_16S_OXY107	32	stramenopiles	Chrysophyceae			HM133470	EF052157	100.00%	Uncultured chrysophyte clone MC615-90 16S ribosomal RNA gene, partialsequence; plastid.
HM133152	STB6	72	55	OXY107	Biosope_T35_16S_OXY107	33	stramenopiles	Chrysophyceae			HM133253	EF052086	99.34%	Uncultured chrysophyte clone MC615-9 16S ribosomal RNA gene, partialsequence; plastid.
HM133153	STB6	72	55	OXY107	Biosope_T35_16S_OXY107	35	stramenopiles	Chrysophyceae			HM133470	EF574962	99.87%	Uncultured bacterium clone S25_1306 16S ribosomal RNA gene, partialsequence.
HM133154	STB6	72	55	OXY107	Biosope_T35_16S_OXY107	37	Stramenopiles	Dictyochophyceae			HM133342	EF052136	99.61%	Uncultured dictyochophyte clone MC615-64 16S ribosomal RNA gene, partialsequence; plastid.
HM133155	STB6	72	55	OXY107	Biosope_T35_16S_OXY107	38	stramenopiles	Chrysophyceae			HM133149	EF052157	99.74%	Uncultured chrysophyte clone MC615-90 16S ribosomal RNA gene, partialsequence; plastid.
HM133156	STB6	72	55	PLA491	Biosope_T35_16S_PLA491	10	Haptophyta	Prymnesiophyceae			HM133549	EF574491	99.47%	Uncultured bacterium clone S25_835 16S ribosomal RNA gene, partialsequence.
HM133157	STB6	72	55	PLA491	Biosope_T35_16S_PLA491	11	Haptophyta	Prymnesiophyceae			HM133549	EF574491	99.61%	Uncultured bacterium clone S25_835 16S ribosomal RNA gene, partialsequence.
HM133158	STB6	72	55	PLA491	Biosope_T35_16S_PLA491	13	stramenopiles	Chrysophyceae			HM133470	EF574962	98.65%	Uncultured bacterium clone S25_1306 16S ribosomal RNA gene, partialsequence.
HM133159	STB6	72	55	PLA491	Biosope_T35_16S_PLA491	14	Haptophyta	Prymnesiophyceae			HM133549	EF574491	99.61%	Uncultured bacterium clone S25_835 16S ribosomal RNA gene, partialsequence.
HM133160	STB6	72	55	PLA491	Biosope_T35_16S_PLA491	15	stramenopiles	Chrysophyceae			HM133470	EF574962	99.73%	Uncultured bacterium clone S25_1306 16S ribosomal RNA gene, partialsequence.
HM133161	STB6	72	55	PLA491	Biosope_T35_16S_PLA491	16	Haptophyta	Prymnesiophyceae			HM133549	EF574491	99.47%	Uncultured bacterium clone S25_835 16S ribosomal RNA gene, partialsequence.
HM133162	STB6	72	55	PLA491	Biosope_T35_16S_PLA491	17	Haptophyta	Prymnesiophyceae			HM133266	FJ649284	99.60%	Uncultured phototrophic eukaryote clone STB1_25m_E10 16S ribosomal RNAgene, partial sequence; chloroplast.
HM133163	STB6	72	55	PLA491	Biosope_T35_16S_PLA491	18	Haptophyta	Prymnesiophyceae			HM133266	EF574386	100.00%	Uncultured bacterium clone S25_730 16S ribosomal RNA gene, partialsequence.
HM133164	STB6	72	55	PLA491	Biosope_T35_16S_PLA491	19	Haptophyta	Prymnesiophyceae			HM133266	EF574945	99.47%	Uncultured bacterium clone S25_1289 16S ribosomal RNA gene, partialsequence.
HM133165	STB6	72	55	PLA491	Biosope_T35_16S_PLA491	20	stramenopiles	Chrysophyceae			HM133253	EU182057	99.73%	Uncultured bacterium clone D15_7_SW_G_30 16S ribosomal RNA gene, partialsequence.
HM133166	STB6	72	55	PLA491	Biosope_T35_16S_PLA491	21	Haptophyta	Prymnesiophyceae			HM133549	EF574491	99.73%	Uncultured bacterium clone S25_835 16S ribosomal RNA gene, partialsequence.
HM133167	STB6	72	55	PLA491	Biosope_T35_16S_PLA491	22	stramenopiles	Chrysophyceae			HM133478	EF052159	98.78%	Uncultured chrysophyte clone MC615-92 16S ribosomal RNA gene, partialsequence; plastid.
HM133168	STB6	72	55	PLA491	Biosope_T35_16S_PLA491	23	Haptophyta	Prymnesiophyceae			HM133503	EF574746	100.00%	Uncultured bacterium clone S25_1090 16S ribosomal RNA gene, partialsequence.
HM133169	STB6	72	55	PLA491	Biosope_T35_16S_PLA491	26	stramenopiles	Chrysophyceae			HM133069	EF052120	98.55%	Uncultured chrysophyte clone MC615-47 16S ribosomal RNA gene, partialsequence; plastid.
HM133170	STB6	72	55	PLA491	Biosope_T35_16S_PLA491	27	stramenopiles	Chrysophyceae			HM133149	EF052157	99.60%	Uncultured chrysophyte clone MC615-90 16S ribosomal RNA gene, partialsequence; plastid.
HM133171	STB6	72	55	PLA491	Biosope_T35_16S_PLA491	28	Haptophyta	Prymnesiophyceae			HM133065	EF574746	99.87%	Uncultured bacterium clone S25_1090 16S ribosomal RNA gene, partialsequence.
HM133172	STB6	72	55	PLA491	Biosope_T35_16S_PLA491	30	Haptophyta	Prymnesiophyceae			HM133065	EF574945	98.95%	Uncultured bacterium clone S25_1289 16S ribosomal RNA gene, partialsequence.
HM133173	STB6	72	55	PLA491	Biosope_T35_16S_PLA491	31	Haptophyta	Prymnesiophyceae			HM133503	AF001655	98.41%	Environmental clone OCS31 small subunit ribosomal RNA gene, chloroplastgene for chloroplast RNA, complete sequence.
HM133174	STB6	72	55	PLA491	Biosope_T35_16S_PLA491	32	stramenopiles	Chrysophyceae			HM133149	EF052157	100.00%	Uncultured chrysophyte clone MC615-90 16S ribosomal RNA gene, partialsequence; plastid.
HM133175	STB6	72	55	PLA491	Biosope_T35_16S_PLA491	33	Haptophyta	Prymnesiophyceae			HM133065	EF574746	100.00%	Uncultured bacterium clone S25_1090 16S ribosomal RNA gene, partialsequence.
HM133176	STB6	72	55	PLA491	Biosope_T35_16S_PLA491	34	stramenopiles	Chrysophyceae			HM133069	EF052120	99.46%	Uncultured chrysophyte clone MC615-47 16S ribosomal RNA gene, partialsequence; plastid.
HM133177	STB6	72	55	PLA491	Biosope_T35_16S_PLA491	35	stramenopiles	Chrysophyceae			HM133470	EF574962	99.73%	Uncultured bacterium clone S25_1306 16S ribosomal RNA gene, partialsequence.
HM133178	STB6	72	55	PLA491	Biosope_T35_16S_PLA491	36	Haptophyta	Prymnesiophyceae			HM133266	EF573710	100.00%	Uncultured bacterium clone S25_54 16S ribosomal RNA gene, partial sequence.
HM133179	STB6	72	55	PLA491	Biosope_T35_16S_PLA491	37	Haptophyta	Prymnesiophyceae			HM133179	EF052033	99.87%	Uncultured haptophyte clone 250304-35 16S ribosomal RNA gene, partialsequence; plastid.
HM133180	STB6	72	55	PLA491	Biosope_T35_16S_PLA491	38	stramenopiles	Chrysophyceae			HM133149	EF052157	99.73%	Uncultured chrysophyte clone MC615-90 16S ribosomal RNA gene, partialsequence; plastid.
HM133181	STB6	72	55	PLA491	Biosope_T35_16S_PLA491	39	Haptophyta	Prymnesiophyceae			HM133503	EF052127	99.87%	Uncultured haptophyte clone MC615-54 16S ribosomal RNA gene, partialsequence; plastid.
HM133182	STB6	72	55	PLA491	Biosope_T35_16S_PLA491	40	stramenopiles	Chrysophyceae			HM133149	EF052157	99.87%	Uncultured chrysophyte clone MC615-90 16S ribosomal RNA gene, partialsequence; plastid.
HM133081	STB6	72	180	OXY107	Biosope_T33_16S_OXY107	1	Stramenopiles	Pelagophyceae			HM133308	DQ513100	99.71%	Uncultured bacterium clone FS274-44B-03 16S ribosomal RNA gene, partialsequence.

Accession	station	CTD cast	depth	primer_forward	Clone_library	Clone	Division	Class	Order	Genus	OTU	BLAST Accession	BLAST % ID	BLAST Description
HM133082	STB6	72	180	OXY107	Biosope_T33_16S_OXY107	2	Stramenopiles	Pelagophyceae			HM133308	DQ513100	99.42%	Uncultured bacterium clone FS274-44B-03 16S ribosomal RNA gene, partialsequence.
HM133083	STB6	72	180	OXY107	Biosope_T33_16S_OXY107	3	Stramenopiles	Pelagophyceae			HM133308	DQ513100	99.56%	Uncultured bacterium clone FS274-44B-03 16S ribosomal RNA gene, partialsequence.
HM133084	STB6	72	180	OXY107	Biosope_T33_16S_OXY107	4	Stramenopiles	Pelagophyceae			HM133308	FJ649273	99.72%	Uncultured phototrophic eukaryote clone STB1_25m_D2 16S ribosomal RNA gene,partial sequence; chloroplast.
HM133085	STB6	72	180	OXY107	Biosope_T33_16S_OXY107	5	Stramenopiles	Pelagophyceae			HM133085	FJ649273	92.19%	Uncultured phototrophic eukaryote clone STB1_25m_D2 16S ribosomal RNA gene,partial sequence; chloroplast.
HM133086	STB6	72	180	OXY107	Biosope_T33_16S_OXY107	6	Stramenopiles	Pelagophyceae			HM133308	FJ649273	99.59%	Uncultured phototrophic eukaryote clone STB1_25m_D2 16S ribosomal RNA gene,partial sequence; chloroplast.
HM133087	STB6	72	180	OXY107	Biosope_T33_16S_OXY107	7	Stramenopiles	Pelagophyceae			HM133308	FJ649273	99.73%	Uncultured phototrophic eukaryote clone STB1_25m_D2 16S ribosomal RNA gene,partial sequence; chloroplast.
HM133088	STB6	72	180	OXY107	Biosope_T33_16S_OXY107	8	Stramenopiles	Pelagophyceae			HM133308	FJ649273	99.73%	Uncultured phototrophic eukaryote clone STB1_25m_D2 16S ribosomal RNA gene,partial sequence; chloroplast.
HM133089	STB6	72	180	OXY107	Biosope_T33_16S_OXY107	9	Stramenopiles	Pelagophyceae			HM133308	DQ513100	99.14%	Uncultured bacterium clone FS274-44B-03 16S ribosomal RNA gene, partialsequence.
HM133090	STB6	72	180	OXY107	Biosope_T33_16S_OXY107	10	Stramenopiles	Pelagophyceae			HM133308	DQ513100	99.71%	Uncultured bacterium clone FS274-44B-03 16S ribosomal RNA gene, partialsequence.
HM133091	STB6	72	180	OXY107	Biosope_T33_16S_OXY107	11	Stramenopiles	Pelagophyceae			HM133308	DQ513100	99.60%	Uncultured bacterium clone FS274-44B-03 16S ribosomal RNA gene, partialsequence.
HM133092	STB6	72	180	OXY107	Biosope_T33_16S_OXY107	12	Stramenopiles	Pelagophyceae			HM133308	DQ513100	99.43%	Uncultured bacterium clone FS274-44B-03 16S ribosomal RNA gene, partialsequence.
HM133093	STB6	72	180	OXY107	Biosope_T33_16S_OXY107	13	Stramenopiles	Pelagophyceae			HM133308	FJ649273	99.86%	Uncultured phototrophic eukaryote clone STB1_25m_D2 16S ribosomal RNA gene,partial sequence; chloroplast.
HM133094	STB6	72	180	OXY107	Biosope_T33_16S_OXY107	15	Stramenopiles	Pelagophyceae			HM133308	DQ513100	100.00%	Uncultured bacterium clone FS274-44B-03 16S ribosomal RNA gene, partialsequence.
HM133095	STB6	72	180	OXY107	Biosope_T33_16S_OXY107	16	Stramenopiles	Pelagophyceae			HM133308	DQ513100	99.57%	Uncultured bacterium clone FS274-44B-03 16S ribosomal RNA gene, partialsequence.
HM133096	STB6	72	180	OXY107	Biosope_T33_16S_OXY107	17	Stramenopiles	Pelagophyceae			HM133308	DQ513100	99.43%	Uncultured bacterium clone FS274-44B-03 16S ribosomal RNA gene, partialsequence.
HM133097	STB6	72	180	OXY107	Biosope_T33_16S_OXY107	18	Stramenopiles	Pelagophyceae			HM133308	DQ513100	99.70%	Uncultured bacterium clone FS274-44B-03 16S ribosomal RNA gene, partialsequence.
HM133098	STB6	72	180	OXY107	Biosope_T33_16S_OXY107	19	Stramenopiles	Pelagophyceae			HM133308	FJ649273	99.71%	Uncultured phototrophic eukaryote clone STB1_25m_D2 16S ribosomal RNA gene,partial sequence; chloroplast.
HM133099	STB6	72	180	OXY107	Biosope_T33_16S_OXY107	21	Stramenopiles	Pelagophyceae			HM133308	FJ649273	99.58%	Uncultured phototrophic eukaryote clone STB1_25m_D2 16S ribosomal RNA gene,partial sequence; chloroplast.
HM133100	STB6	72	180	OXY107	Biosope_T33_16S_OXY107	22	Chlorophyta	Prasinophyceae	clade VII		HM133100	AY702147	100.00%	Coccoid prasinophyte sp. RCC287 16S ribosomal RNA gene, partial sequence;plastid.
HM133101	STB6	72	180	OXY107	Biosope_T33_16S_OXY107	23	Stramenopiles	Pelagophyceae			HM133308	FJ649273	99.86%	Uncultured phototrophic eukaryote clone STB1_25m_D2 16S ribosomal RNA gene,partial sequence; chloroplast.
HM133102	STB6	72	180	OXY107	Biosope_T33_16S_OXY107	24	Stramenopiles	Pelagophyceae			HM133308	FJ649273	99.60%	Uncultured phototrophic eukaryote clone STB1_25m_D2 16S ribosomal RNA gene,partial sequence; chloroplast.
HM133103	STB6	72	180	PLA491	Biosope_T33_16S_PLA491	1	Haptophyta	Prymnesiophyceae			HM133546	EF574745	99.33%	Uncultured bacterium clone S25_1089 16S ribosomal RNA gene, partialsequence.
HM133104	STB6	72	180	PLA491	Biosope_T33_16S_PLA491	2	Haptophyta	Prymnesiophyceae			HM133317	X65101	97.23%	Ochrosphaera sp. 181 plastid RNA for 16S like rRNA small subunit
HM133105	STB6	72	180	PLA491	Biosope_T33_16S_PLA491	4	Haptophyta	Prymnesiophyceae			HM133317	X65101	97.41%	Ochrosphaera sp. 181 plastid RNA for 16S like rRNA small subunit
HM133106	STB6	72	180	PLA491	Biosope_T33_16S_PLA491	5	Stramenopiles	Pelagophyceae			HM133106	FJ649273	94.25%	Uncultured phototrophic eukaryote clone STB1_25m_D2 16S ribosomal RNA gene,partial sequence; chloroplast.
HM133107	STB6	72	180	PLA491	Biosope_T33_16S_PLA491	6	Haptophyta	Prymnesiophyceae			HM133317	X65101	97.39%	Ochrosphaera sp. 181 plastid RNA for 16S like rRNA small subunit
HM133108	STB6	72	180	PLA491	Biosope_T33_16S_PLA491	7	Stramenopiles	Pelagophyceae			HM133308	FJ649273	99.73%	Uncultured phototrophic eukaryote clone STB1_25m_D2 16S ribosomal RNA gene,partial sequence; chloroplast.
HM133109	STB6	72	180	PLA491	Biosope_T33_16S_PLA491	8	Haptophyta	Prymnesiophyceae			HM133546	X65101	94.68%	Ochrosphaera sp. 181 plastid RNA for 16S like rRNA small subunit
HM133110	STB6	72	180	PLA491	Biosope_T33_16S_PLA491	9	Haptophyta	Prymnesiophyceae			HM133546	X65101	94.72%	Ochrosphaera sp. 181 plastid RNA for 16S like rRNA small subunit
HM133111	STB6	72	180	PLA491	Biosope_T33_16S_PLA491	10	Haptophyta	Prymnesiophyceae			HM133317	X65101	97.16%	Ochrosphaera sp. 181 plastid RNA for 16S like rRNA small subunit
HM133112	STB6	72	180	PLA491	Biosope_T33_16S_PLA491	11	Stramenopiles	Pelagophyceae			HM133308	DQ513100	99.73%	Uncultured bacterium clone FS274-44B-03 16S ribosomal RNA gene, partialsequence.
HM133113	STB6	72	180	PLA491	Biosope_T33_16S_PLA491	12	Haptophyta	Prymnesiophyceae			HM133339	DQ395642	99.34%	Uncultured organism clone ctg_CGOF234 16S ribosomal RNA gene, partialsequence.
HM133114	STB6	72	180	PLA491	Biosope_T33_16S_PLA491	13	Stramenopiles	Pelagophyceae			HM133308	FJ649273	99.60%	Uncultured phototrophic eukaryote clone STB1_25m_D2 16S ribosomal RNA gene,partial sequence; chloroplast.
HM133115	STB6	72	180	PLA491	Biosope_T33_16S_PLA491	15	Stramenopiles	Pelagophyceae			HM133308	FJ649273	99.73%	Uncultured phototrophic eukaryote clone STB1_25m_D2 16S ribosomal RNA gene,partial sequence; chloroplast.
HM133116	STB6	72	180	PLA491	Biosope_T33_16S_PLA491	16	Stramenopiles	Pelagophyceae			HM133308	FJ649273	99.73%	Uncultured phototrophic eukaryote clone STB1_25m_D2 16S ribosomal RNA gene,partial sequence; chloroplast.
HM133117	STB6	72	180	PLA491	Biosope_T33_16S_PLA491	17	Stramenopiles	Pelagophyceae			HM133308	DQ513100	99.47%	Uncultured bacterium clone FS274-44B-03 16S ribosomal RNA gene, partialsequence.
HM133118	STB6	72	180	PLA491	Biosope_T33_16S_PLA491	18	Haptophyta	Prymnesiophyceae			HM133065	EF052003	99.47%	Uncultured haptophyte clone 250304-3 16S ribosomal RNA gene, partialsequence; plastid.
HM133119	STB6	72	180	PLA491	Biosope_T33_16S_PLA491	19	Stramenopiles	Pelagophyceae			HM133308	FJ649273	99.60%	Uncultured phototrophic eukaryote clone STB1_25m_D2 16S ribosomal RNA gene,partial sequence; chloroplast.
HM133120	STB6	72	180	PLA491	Biosope_T33_16S_PLA491	20	Haptophyta	Prymnesiophyceae			HM133317	X65101	97.50%	Ochrosphaera sp. 181 plastid RNA for 16S like rRNA small subunit
HM133121	STB6	72	180	PLA491	Biosope_T33_16S_PLA491	21	Stramenopiles	Pelagophyceae			HM133308	FJ649273	98.91%	Uncultured phototrophic eukaryote clone STB1_25m_D2 16S ribosomal RNA gene,partial sequence; chloroplast.
HM133122	STB6	72	180	PLA491	Biosope_T33_16S_PLA491	22	Haptophyta	Prymnesiophyceae			HM133317	FJ826362	95.36%	Uncultured marine bacterium clone A6-3-54 16S ribosomal RNA gene, partialsequence.
HM133123	STB6	72	180	PLA491	Biosope_T33_16S_PLA491	23	Stramenopiles	Pelagophyceae			HM133308	FJ649273	99.60%	Uncultured phototrophic eukaryote clone STB1_25m_D2 16S ribosomal RNA gene,partial sequence; chloroplast.
HM133124	STB6	72	180	PLA491	Biosope_T33_16S_PLA491	24	Stramenopiles	Pelagophyceae			HM133308	FJ649273	99.60%	Uncultured phototrophic eukaryote clone STB1_25m_D2 16S ribosomal RNA gene,partial sequence; chloroplast.
HM133125	STB6	72	180	PLA491	Biosope_T33_16S_PLA491	25	Stramenopiles	Pelagophyceae			HM133308	FJ649273	99.60%	Uncultured phototrophic eukaryote clone STB1_25m_D2 16S ribosomal RNA gene,partial sequence; chloroplast.
HM133126	STB6	72	180	PLA491	Biosope_T33_16S_PLA491	26	Haptophyta	Prymnesiophyceae			HM133317	X65101	97.09%	Ochrosphaera sp. 181 plastid RNA for 16S like rRNA small subunit
HM133127	STB6	72	180	PLA491	Biosope_T33_16S_PLA491	27	Haptophyta	Prymnesiophyceae			HM133317	X65101	97.36%	Ochrosphaera sp. 181 plastid RNA for 16S like rRNA small subunit
HM133128	STB6	72	180	PLA491	Biosope_T33_16S_PLA491	28	Haptophyta	Prymnesiophyceae			HM133317	X65101	96.97%	Ochrosphaera sp. 181 plastid RNA for 16S like rRNA small subunit
HM133129	STB6	72	180	PLA491	Biosope_T33_16S_PLA491	29	Haptophyta	Prymnesiophyceae			HM133339	DQ395642	99.47%	Uncultured organism clone ctg_CGOF234 16S ribosomal RNA gene, partialsequence.
HM133130	STB6	72	180	PLA491	Biosope_T33_16S_PLA491	30	Stramenopiles	Pelagophyceae			HM133308	FJ649273	99.60%	Uncultured phototrophic eukaryote clone STB1_25m_D2 16S ribosomal RNA gene,partial sequence; chloroplast.
HM133131	STB6	72	180	PLA491	Biosope_T33_16S_PLA491	32	Haptophyta	Prymnesiophyceae			HM133546	EF574745	99.87%	Uncultured bacterium clone S25_1089 16S ribosomal RNA gene, partialsequence.
HM133234	STB7	76	40	OXY107	Biosope_T41_16S_OXY107	2	Chlorophyta	Prasinophyceae	clade 16S-IX		HM133248	DQ438491	93.41%	Uncultured bacterium clone ECS-P7-D55 16S ribosomal RNA gene, partialsequence.
HM133235	STB7	76	40	OXY107	Biosope_T41_16S_OXY107	4	stramenopiles	Chrysophyceae			HM133470	EF574962	99.56%	Uncultured bacterium clone S25_1306 16S ribosomal RNA gene, partialsequence.
HM133236	STB7	76	40	OXY107	Biosope_T41_16S_OXY107	5	stramenopiles	Chrysophyceae			HM133470	EF574962	99.56%	Uncultured bacterium clone S25_1306 16S ribosomal RNA gene, partialsequence.
HM133237	STB7	76	40	OXY107	Biosope_T41_16S_OXY107	7	Chlorophyta	Prasinophyceae	clade 16S-IX		HM133248	DQ438491	93.38%	Uncultured bacterium clone ECS-P7-D55 16S ribosomal RNA gene, partialsequence.
HM133238	STB7	76	40	OXY107	Biosope_T41_16S_OXY107	8	stramenopiles	Chrysophyceae			HM133470	FJ649291	98.76%	Uncultured phototrophic eukaryote clone STB1_25m_F10 16S ribosomal RNAgene, partial sequence; chloroplast.
HM133239	STB7	76	40	OXY107	Biosope_T41_16S_OXY107	10	Chlorophyta	Prasinophyceae	clade 16S-IX		HM133267	FJ649270	98.60%	Uncultured phototrophic eukaryote clone STB1_25m_C10 16S ribosomal RNAgene, partial sequence; chloroplast.
HM133240	STB7	76	40	OXY107	Biosope_T41_16S_OXY107	11	Chlorophyta	Prasinophyceae	clade 16S-IX		HM133471	DQ438491	99.32%	Uncultured bacterium clone ECS-P7-D55 16S ribosomal RNA gene, partialsequence.
HM133241	STB7	76	40	OXY107	Biosope_T41_16S_OXY107	12	stramenopiles	Chrysophyceae			HM133478	EF052159	99.31%	Uncultured chrysophyte clone MC615-92 16S ribosomal RNA gene, partialsequence; plastid.
HM133242	STB7	76	40	OXY107	Biosope_T41_16S_OXY107	13	Stramenopiles	Dictyochophyceae			HM133300	FJ826020	99.72%	Uncultured phototrophic eukaryote clone BM1-8-32 16S ribosomal RNA gene,partial sequence; plastid.
HM133243	STB7	76	40	OXY107	Biosope_T41_16S_OXY107	14	stramenopiles	Dictyochophyceae			HM133342	FJ649290	99.87%	Uncultured phototrophic eukaryote clone STB1_25m_F8 16S ribosomal RNA gene,partial sequence; chloroplast.
HM133244	STB7	76	40	OXY107	Biosope_T41_16S_OXY107	15	Chlorophyta	Prasinophyceae	clade 16S-IX		HM133244	DQ438491	93.50%	Uncultured bacterium clone ECS-P7-D55 16S ribosomal RNA gene, partialsequence.
HM133245	STB7	76	40	OXY107	Biosope_T41_16S_OXY107	16	stramenopiles	Chrysophyceae			HM133478	EF052159	99.73%	Uncultured chrysophyte clone MC615-92 16S ribosomal RNA gene, partialsequence; plastid.
HM133246	STB7	76	40	OXY107	Biosope_T41_16S_OXY107	17	stramenopiles	Dictyochophyceae			HM133342	EF052136	99.06%	Uncultured dictyochophyte clone MC615-64 16S ribosomal RNA gene, partialsequence; plastid.
HM133247	STB7	76	40	OXY107	Biosope_T41_16S_OXY107	18	stramenopiles	Chrysophyceae			HM133149	EF052157	99.72%	Uncultured chrysophyte clone MC615-90 16S ribosomal RNA gene, partialsequence; plastid.
HM133248	STB7	76	40	OXY107	Biosope_T41_16S_OXY107	27	Chlorophyta	Prasinophyceae	clade 16S-IX		HM133248	DQ438491	92.85%	Uncultured bacterium clone ECS-P7-D55 16S ribosomal RNA gene, partialsequence.
HM133249	STB7	76	40	OXY107	Biosope_T41_16S_OXY107	28	stramenopiles	Chrysophyceae			HM133249	EF574962	88.34%	Uncultured bacterium clone S25_1306 16S ribosomal RNA gene, partialsequence.
HM133250	STB7	76	40	OXY107	Biosope_T41_16S_OXY107	30	stramenopiles	Dictyochophyceae			HM133342	EF052136	99.47%	Uncultured dictyochophyte clone MC615-64 16S ribosomal RNA gene, partialsequence; plastid.

Accession	station	CTD cast	depth	primer_forward	Clone_library	Clone	Division	Class	Order	Genus	OTU	BLAST Accession	BLAST % ID	BLAST Description
HM133251	STB7	76	40	OXY107	Biosope_T41_16S_OXY107	31	Chlorophyta	Prasinophyceae	clade 16S-IX		HM133471	DQ438491	99.31%	Uncultured bacterium clone ECS-P7-D55 16S ribosomal RNA gene, partialsequence.
HM133252	STB7	76	40	OXY107	Biosope_T41_16S_OXY107	32	Chlorophyta	Prasinophyceae	clade 16S-IX		HM133248	DQ438491	93.19%	Uncultured bacterium clone ECS-P7-D55 16S ribosomal RNA gene, partialsequence.
HM133253	STB7	76	40	PLA491	Biosope_T41_16S_PLA491	4	stramenopiles	Chrysophyceae			HM133253	EF052086	99.61%	Uncultured chrysophyte clone MC615-9 16S ribosomal RNA gene, partialsequence; plastid.
HM133254	STB7	76	40	PLA491	Biosope_T41_16S_PLA491	10	stramenopiles	Chrysophyceae			HM133470	EF574962	99.87%	Uncultured bacterium clone S25_1306 16S ribosomal RNA gene, partialsequence.
HM133255	STB7	76	40	PLA491	Biosope_T41_16S_PLA491	13	stramenopiles	Chrysophyceae			HM133149	EF052157	99.73%	Uncultured chrysophyte clone MC615-90 16S ribosomal RNA gene, partialsequence; plastid.
HM133256	STB7	76	40	PLA491	Biosope_T41_16S_PLA491	18	stramenopiles	Chrysophyceae			HM133253	EU182057	98.54%	Uncultured bacterium clone D15_7_SW_G_30 16S ribosomal RNA gene, partialsequence.
HM133257	STB7	76	40	PLA491	Biosope_T41_16S_PLA491	28	stramenopiles	Chrysophyceae			HM133149	EF052157	100.00%	Uncultured chrysophyte clone MC615-90 16S ribosomal RNA gene, partialsequence; plastid.
HM133258	STB7	76	40	PLA491	Biosope_T41_16S_PLA491	29	stramenopiles	Chrysophyceae			HM133258	FJ649289	96.66%	Uncultured phototrophic eukaryote clone STB1_25m_F6 16S ribosomal RNA gene,partial sequence; chloroplast.
HM133259	STB7	76	40	PLA491	Biosope_T41_16S_PLA491	33	Stramenopiles	Chrysophyceae			HM133259	FJ745195	97.73%	Uncultured bacterium clone SHWN_night2_16S_759 16S ribosomal RNA gene,partial sequence.
HM133260	STB7	76	40	PLA491	Biosope_T41_16S_PLA491	38	stramenopiles	Chrysophyceae			HM133478	EF052159	97.18%	Uncultured chrysophyte clone MC615-92 16S ribosomal RNA gene, partialsequence; plastid.
HM133261	STB7	76	40	PLA491	Biosope_T41_16S_PLA491	62	Haptophyta	Prymnesiophyceae			HM133486	EF052047	99.87%	Uncultured haptophyte clone 250304-50 16S ribosomal RNA gene, partialsequence; plastid.
HM133262	STB7	76	40	PLA491	Biosope_T41_16S_PLA491	63	Chlorophyta	Prasinophyceae	clade 16S-IX		HM133248	DQ438491	92.89%	Uncultured bacterium clone ECS-P7-D55 16S ribosomal RNA gene, partialsequence.
HM133263	STB7	76	40	PLA491	Biosope_T41_16S_PLA491	64	stramenopiles	Chrysophyceae			HM133478	EF052159	99.47%	Uncultured chrysophyte clone MC615-92 16S ribosomal RNA gene, partialsequence; plastid.
HM133264	STB7	76	40	PLA491	Biosope_T41_16S_PLA491	79	Chlorophyta	Prasinophyceae	clade 16S-IX		HM133244	DQ438491	93.79%	Uncultured bacterium clone ECS-P7-D55 16S ribosomal RNA gene, partialsequence.
HM133265	STB7	76	40	PLA491	Biosope_T41_16S_PLA491	92	stramenopiles	Chrysophyceae			HM133478	EF052159	99.29%	Uncultured chrysophyte clone MC615-92 16S ribosomal RNA gene, partialsequence; plastid.
HM133266	STB7	76	40	PLA491	Biosope_T41_16S_PLA491	106	Haptophyta	Prymnesiophyceae			HM133266	EF052162	98.83%	Uncultured haptophyte clone MC615-95 16S ribosomal RNA gene, partialsequence; plastid.
HM133267	STB7	76	40	PLA491	Biosope_T41_16S_PLA491	109	Chlorophyta	Prasinophyceae	clade 16S-IX		HM133267	FJ649270	95.47%	Uncultured phototrophic eukaryote clone STB1_25m_C10 16S ribosomal RNAgene, partial sequence; chloroplast.
HM133268	STB7	76	40	PLA491	Biosope_T41_16S_PLA491	130	Haptophyta	Prymnesiophyceae			HM133266	EF574441	99.87%	Uncultured bacterium clone S25_785 16S ribosomal RNA gene, partialsequence.
HM133269	STB7	76	40	PLA491	Biosope_T41_16S_PLA491	150	stramenopiles	Chrysophyceae			HM133478	EF052159	95.98%	Uncultured chrysophyte clone MC615-92 16S ribosomal RNA gene, partialsequence; plastid.
HM133270	STB7	76	40	PLA491	Biosope_T41_16S_PLA491	153	Chlorophyta	Prasinophyceae	clade 16S-VIII		HM133270	FJ649288	99.19%	Uncultured phototrophic eukaryote clone STB1_25m_F4 16S ribosomal RNA gene,partial sequence; chloroplast.
HM133271	STB7	76	40	PLA491	Biosope_T41_16S_PLA491	155	Haptophyta	Prymnesiophyceae			HM133266	EF573710	100.00%	Uncultured bacterium clone S25_54 16S ribosomal RNA gene, partial sequence.
HM133183	STB7	76	175	OXY107	Biosope_T39_16S_OXY107	1	Stramenopiles	Pelagophyceae			HM133308	FJ649273	99.47%	Uncultured phototrophic eukaryote clone STB1_25m_D2 16S ribosomal RNA gene,partial sequence; chloroplast.
HM133184	STB7	76	175	OXY107	Biosope_T39_16S_OXY107	2	Stramenopiles	Pelagophyceae			HM133308	FJ649273	99.48%	Uncultured phototrophic eukaryote clone STB1_25m_D2 16S ribosomal RNA gene,partial sequence; chloroplast.
HM133185	STB7	76	175	OXY107	Biosope_T39_16S_OXY107	3	Stramenopiles	Pelagophyceae			HM133308	FJ649273	99.74%	Uncultured phototrophic eukaryote clone STB1_25m_D2 16S ribosomal RNA gene,partial sequence; chloroplast.
HM133186	STB7	76	175	OXY107	Biosope_T39_16S_OXY107	5	Stramenopiles	Dictyochophyceae			HM133300	FJ826020	98.95%	Uncultured phototrophic eukaryote clone BM1-8-32 16S ribosomal RNA gene,partial sequence; plastid.
HM133187	STB7	76	175	OXY107	Biosope_T39_16S_OXY107	7	Stramenopiles	Pelagophyceae			HM133308	FJ649273	99.87%	Uncultured phototrophic eukaryote clone STB1_25m_D2 16S ribosomal RNA gene,partial sequence; chloroplast.
HM133188	STB7	76	175	OXY107	Biosope_T39_16S_OXY107	8	Stramenopiles	Pelagophyceae			HM133308	FJ649273	99.47%	Uncultured phototrophic eukaryote clone STB1_25m_D2 16S ribosomal RNA gene,partial sequence; chloroplast.
HM133189	STB7	76	175	OXY107	Biosope_T39_16S_OXY107	9	Stramenopiles	Pelagophyceae			HM133308	FJ649273	99.21%	Uncultured phototrophic eukaryote clone STB1_25m_D2 16S ribosomal RNA gene,partial sequence; chloroplast.
HM133190	STB7	76	175	OXY107	Biosope_T39_16S_OXY107	10	Stramenopiles	Pelagophyceae			HM133308	FJ649273	99.47%	Uncultured phototrophic eukaryote clone STB1_25m_D2 16S ribosomal RNA gene,partial sequence; chloroplast.
HM133191	STB7	76	175	OXY107	Biosope_T39_16S_OXY107	11	Stramenopiles	Pelagophyceae			HM133308	GQ231541	97.63%	Aureococcus anophagefferens strain CCMP 1984 chloroplast, complete genome.
HM133192	STB7	76	175	OXY107	Biosope_T39_16S_OXY107	12	Stramenopiles	Pelagophyceae			HM133308	FJ649273	99.08%	Uncultured phototrophic eukaryote clone STB1_25m_D2 16S ribosomal RNA gene,partial sequence; chloroplast.
HM133193	STB7	76	175	OXY107	Biosope_T39_16S_OXY107	13	Stramenopiles	Pelagophyceae			HM133308	FJ649273	99.74%	Uncultured phototrophic eukaryote clone STB1_25m_D2 16S ribosomal RNA gene,partial sequence; chloroplast.
HM133194	STB7	76	175	OXY107	Biosope_T39_16S_OXY107	15	Stramenopiles	Pelagophyceae			HM133308	FJ649273	99.60%	Uncultured phototrophic eukaryote clone STB1_25m_D2 16S ribosomal RNA gene,partial sequence; chloroplast.
HM133195	STB7	76	175	OXY107	Biosope_T39_16S_OXY107	17	stramenopiles	Chrysophyceae			HM133470	EF574962	98.67%	Uncultured bacterium clone S25_1306 16S ribosomal RNA gene, partialsequence.
HM133196	STB7	76	175	OXY107	Biosope_T39_16S_OXY107	18	Stramenopiles	Pelagophyceae			HM133308	FJ649273	99.74%	Uncultured phototrophic eukaryote clone STB1_25m_D2 16S ribosomal RNA gene,partial sequence; chloroplast.
HM133197	STB7	76	175	OXY107	Biosope_T39_16S_OXY107	19	Stramenopiles	Pelagophyceae			HM133308	FJ649273	99.34%	Uncultured phototrophic eukaryote clone STB1_25m_D2 16S ribosomal RNA gene,partial sequence; chloroplast.
HM133198	STB7	76	175	OXY107	Biosope_T39_16S_OXY107	21	Stramenopiles	Pelagophyceae			HM133308	FJ649273	100.00%	Uncultured phototrophic eukaryote clone STB1_25m_D2 16S ribosomal RNA gene,partial sequence; chloroplast.
HM133199	STB7	76	175	OXY107	Biosope_T39_16S_OXY107	22	Stramenopiles	Pelagophyceae			HM133308	FJ649273	99.34%	Uncultured phototrophic eukaryote clone STB1_25m_D2 16S ribosomal RNA gene,partial sequence; chloroplast.
HM133200	STB7	76	175	OXY107	Biosope_T39_16S_OXY107	24	Stramenopiles	Pelagophyceae			HM133308	FJ649273	100.00%	Uncultured phototrophic eukaryote clone STB1_25m_D2 16S ribosomal RNA gene,partial sequence; chloroplast.
HM133201	STB7	76	175	OXY107	Biosope_T39_16S_OXY107	25	Stramenopiles	Pelagophyceae			HM133308	FJ649273	99.60%	Uncultured phototrophic eukaryote clone STB1_25m_D2 16S ribosomal RNA gene,partial sequence; chloroplast.
HM133202	STB7	76	175	OXY107	Biosope_T39_16S_OXY107	27	Stramenopiles	Pelagophyceae			HM133308	FJ649273	99.61%	Uncultured phototrophic eukaryote clone STB1_25m_D2 16S ribosomal RNA gene,partial sequence; chloroplast.
HM133203	STB7	76	175	OXY107	Biosope_T39_16S_OXY107	29	Stramenopiles	Pelagophyceae			HM133308	FJ649273	99.34%	Uncultured phototrophic eukaryote clone STB1_25m_D2 16S ribosomal RNA gene,partial sequence; chloroplast.
HM133204	STB7	76	175	OXY107	Biosope_T39_16S_OXY107	31	stramenopiles	Chrysophyceae			HM133149	EF573915	100.00%	Uncultured bacterium clone S25_259 16S ribosomal RNA gene, partialsequence.
HM133205	STB7	76	175	OXY107	Biosope_T39_16S_OXY107	32	Stramenopiles	Pelagophyceae			HM133308	FJ649273	99.85%	Uncultured phototrophic eukaryote clone STB1_25m_D2 16S ribosomal RNA gene,partial sequence; chloroplast.
HM133206	STB7	76	175	PLA491	Biosope_T39_16S_PLA491	1	Stramenopiles	Pelagophyceae			HM133308	FJ649273	99.73%	Uncultured phototrophic eukaryote clone STB1_25m_D2 16S ribosomal RNA gene,partial sequence; chloroplast.
HM133207	STB7	76	175	PLA491	Biosope_T39_16S_PLA491	2	stramenopiles	Chrysophyceae			HM133470	EF574962	98.26%	Uncultured bacterium clone S25_1306 16S ribosomal RNA gene, partialsequence.
HM133208	STB7	76	175	PLA491	Biosope_T39_16S_PLA491	3	Haptophyta	Prymnesiophyceae			HM133546	EF574745	100.00%	Uncultured bacterium clone S25_1089 16S ribosomal RNA gene, partialsequence.
HM133209	STB7	76	175	PLA491	Biosope_T39_16S_PLA491	5	Stramenopiles	Pelagophyceae			HM133308	FJ649273	99.60%	Uncultured phototrophic eukaryote clone STB1_25m_D2 16S ribosomal RNA gene,partial sequence; chloroplast.
HM133210	STB7	76	175	PLA491	Biosope_T39_16S_PLA491	6	stramenopiles	Chrysophyceae			HM133478	EF052159	98.51%	Uncultured chrysophyte clone MC615-92 16S ribosomal RNA gene, partialsequence; plastid.
HM133211	STB7	76	175	PLA491	Biosope_T39_16S_PLA491	7	stramenopiles	Chrysophyceae			HM133478	EF052159	98.79%	Uncultured chrysophyte clone MC615-92 16S ribosomal RNA gene, partialsequence; plastid.
HM133212	STB7	76	175	PLA491	Biosope_T39_16S_PLA491	8	Stramenopiles	Pelagophyceae			HM133308	FJ649273	99.60%	Uncultured phototrophic eukaryote clone STB1_25m_D2 16S ribosomal RNA gene,partial sequence; chloroplast.
HM133213	STB7	76	175	PLA491	Biosope_T39_16S_PLA491	9	stramenopiles	Chrysophyceae			HM133470	EF574962	98.53%	Uncultured bacterium clone S25_1306 16S ribosomal RNA gene, partialsequence.
HM133214	STB7	76	175	PLA491	Biosope_T39_16S_PLA491	10	stramenopiles	Chrysophyceae			HM133069	AY702190	99.46%	Uncultured phototrophic eukaryote clone AS2_50C23 16S ribosomal RNA gene,partial sequence; plastid.
HM133215	STB7	76	175	PLA491	Biosope_T39_16S_PLA491	11	Haptophyta	Prymnesiophyceae			HM133339	DQ395642	99.73%	Uncultured organism clone ctg_CGOF234 16S ribosomal RNA gene, partialsequence.
HM133216	STB7	76	175	PLA491	Biosope_T39_16S_PLA491	13	stramenopiles	Chrysophyceae			HM133470	EF574962	98.65%	Uncultured bacterium clone S25_1306 16S ribosomal RNA gene, partialsequence.
HM133217	STB7	76	175	PLA491	Biosope_T39_16S_PLA491	14	Haptophyta	Prymnesiophyceae			HM133337	EF052069	99.74%	Uncultured haptophyte clone 250304-84 16S ribosomal RNA gene, partialsequence; plastid.
HM133218	STB7	76	175	PLA491	Biosope_T39_16S_PLA491	15	Stramenopiles	Pelagophyceae			HM133308	FJ649273	99.60%	Uncultured phototrophic eukaryote clone STB1_25m_D2 16S ribosomal RNA gene,partial sequence; chloroplast.
HM133219	STB7	76	175	PLA491	Biosope_T39_16S_PLA491	16	Stramenopiles	Pelagophyceae			HM133308	FJ649273	99.34%	Uncultured phototrophic eukaryote clone STB1_25m_D2 16S ribosomal RNA gene,partial sequence; chloroplast.
HM133220	STB7	76	175	PLA491	Biosope_T39_16S_PLA491	17	stramenopiles	Chrysophyceae			HM133478	EF052159	98.54%	Uncultured chrysophyte clone MC615-92 16S ribosomal RNA gene, partialsequence; plastid.
HM133221	STB7	76	175	PLA491	Biosope_T39_16S_PLA491	18	Stramenopiles	Pelagophyceae			HM133308	FJ649273	99.60%	Uncultured phototrophic eukaryote clone STB1_25m_D2 16S ribosomal RNA gene,partial sequence; chloroplast.
HM133222	STB7	76	175	PLA491	Biosope_T39_16S_PLA491	19	Haptophyta	Prymnesiophyceae			HM133339	DQ395642	99.47%	Uncultured organism clone ctg_CGOF234 16S ribosomal RNA gene, partialsequence.
HM133223	STB7	76	175	PLA491	Biosope_T39_16S_PLA491	20	Stramenopiles	Pelagophyceae			HM133308	FJ649273	99.47%	Uncultured phototrophic eukaryote clone STB1_25m_D2 16S ribosomal RNA gene,partial sequence; chloroplast.
HM133224	STB7	76	175	PLA491	Biosope_T39_16S_PLA491	21	Stramenopiles	Pelagophyceae			HM133308	FJ649273	99.60%	Uncultured phototrophic eukaryote clone STB1_25m_D2 16S ribosomal RNA gene,partial sequence; chloroplast.
HM133225	STB7	76	175	PLA491	Biosope_T39_16S_PLA491	22	stramenopiles	Chrysophyceae			HM133478	EF052159	98.79%	Uncultured chrysophyte clone MC615-92 16S ribosomal RNA gene, partialsequence; plastid.
HM133226	STB7	76	175	PLA491	Biosope_T39_16S_PLA491	23	Stramenopiles	Pelagophyceae			HM133308	FJ649273	99.60%	Uncultured phototrophic eukaryote clone STB1_25m_D2 16S ribosomal RNA gene,partial sequence; chloroplast.
HM133227	STB7	76	175	PLA491	Biosope_T39_16S_PLA491	24	Stramenopiles	Pelagophyceae			HM133308	FJ649273	99.60%	Uncultured phototrophic eukaryote clone STB1_25m_D2 16S ribosomal RNA gene,partial sequence; chloroplast.
HM133228	STB7	76	175	PLA491	Biosope_T39_16S_PLA491	25	stramenopiles	Chrysophyceae			HM133069	AY702190	98.92%	Uncultured phototrophic eukaryote clone AS2_50C23 16S ribosomal RNA gene,partial sequence; plastid.

Accession	station	CTD cast	depth	primer_forward	Clone_library	Clone	Division	Class	Order	Genus	OTU	BLAST Accession	BLAST % ID	BLAST Description
HM133229	STB7	76	175	PLA491	Biosope_T39_16S_PLA491	26	stramenopiles	Chrysophyceae			HM133478	EF052159	99.06%	Uncultured chrysophyte clone MC615-92 16S ribosomal RNA gene, partialsequence; plastid.
HM133230	STB7	76	175	PLA491	Biosope_T39_16S_PLA491	28	Haptophyta	Prymnesiophyceae			HM133339	DQ395642	98.80%	Uncultured organism clone ctg_CGOF234 16S ribosomal RNA gene, partialsequence.
HM133231	STB7	76	175	PLA491	Biosope_T39_16S_PLA491	30	Stramenopiles	Pelagophyceae			HM133308	FJ649273	99.46%	Uncultured phototrophic eukaryote clone STB1_25m_D2 16S ribosomal RNA gene,partial sequence; chloroplast.
HM133232	STB7	76	175	PLA491	Biosope_T39_16S_PLA491	31	Stramenopiles	Dictyochophyceae			HM133342	FJ649290	99.73%	Uncultured phototrophic eukaryote clone STB1_25m_F8 16S ribosomal RNA gene,partial sequence; chloroplast.
HM133233	STB7	76	175	PLA491	Biosope_T39_16S_PLA491	32	Stramenopiles	Pelagophyceae			HM133308	FJ649273	99.47%	Uncultured phototrophic eukaryote clone STB1_25m_D2 16S ribosomal RNA gene,partial sequence; chloroplast.
HM133341	STB11	121	0	OXY107	Biosope_T60_16S_OXY107	1	stramenopiles	Dictyochophyceae			HM133342	EF052136	99.34%	Uncultured dictyochophyte clone MC615-64 16S ribosomal RNA gene, partialsequence; plastid.
HM133342	STB11	121	0	OXY107	Biosope_T60_16S_OXY107	2	Stramenopiles	Dictyochophyceae			HM133342	FJ649290	99.87%	Uncultured phototrophic eukaryote clone STB1_25m_F8 16S ribosomal RNA gene,partial sequence; chloroplast.
HM133343	STB11	121	0	OXY107	Biosope_T60_16S_OXY107	4	Haptophyta	Prymnesiophyceae			HM133343	FJ649286	97.38%	Uncultured phototrophic eukaryote clone STB1_25m_E12 16S ribosomal RNAgene, partial sequence; chloroplast.
HM133344	STB11	121	0	OXY107	Biosope_T60_16S_OXY107	5	stramenopiles	Chrysophyceae			HM133478	EF052159	99.87%	Uncultured chrysophyte clone MC615-92 16S ribosomal RNA gene, partialsequence; plastid.
HM133345	STB11	121	0	OXY107	Biosope_T60_16S_OXY107	10	Stramenopiles	Dictyochophyceae			HM133342	FJ649290	99.74%	Uncultured phototrophic eukaryote clone STB1_25m_F8 16S ribosomal RNA gene,partial sequence; chloroplast.
HM133346	STB11	121	0	OXY107	Biosope_T60_16S_OXY107	11	Stramenopiles	Dictyochophyceae			HM133342	FJ649290	99.60%	Uncultured phototrophic eukaryote clone STB1_25m_F8 16S ribosomal RNA gene,partial sequence; chloroplast.
HM133347	STB11	121	0	OXY107	Biosope_T60_16S_OXY107	13	stramenopiles	Dictyochophyceae			HM133342	EF052136	99.61%	Uncultured dictyochophyte clone MC615-64 16S ribosomal RNA gene, partialsequence; plastid.
HM133348	STB11	121	0	OXY107	Biosope_T60_16S_OXY107	14	Stramenopiles	Dictyochophyceae			HM133342	FJ649290	99.74%	Uncultured phototrophic eukaryote clone STB1_25m_F8 16S ribosomal RNA gene,partial sequence; chloroplast.
HM133349	STB11	121	0	OXY107	Biosope_T60_16S_OXY107	15	Stramenopiles	Dictyochophyceae			HM133342	EF052136	99.47%	Uncultured dictyochophyte clone MC615-64 16S ribosomal RNA gene, partialsequence; plastid.
HM133350	STB11	121	0	OXY107	Biosope_T60_16S_OXY107	17	stramenopiles	Chrysophyceae			HM133478	EF052159	99.87%	Uncultured chrysophyte clone MC615-92 16S ribosomal RNA gene, partialsequence; plastid.
HM133351	STB11	121	0	OXY107	Biosope_T60_16S_OXY107	19	Stramenopiles	Dictyochophyceae			HM133342	EF052136	99.35%	Uncultured dictyochophyte clone MC615-64 16S ribosomal RNA gene, partialsequence; plastid.
HM133352	STB11	121	0	OXY107	Biosope_T60_16S_OXY107	20	Chlorophyta	Prasinophyceae	clade 16S-IX		HM133248	DQ438491	93.37%	Uncultured bacterium clone ECS-P7-D55 16S ribosomal RNA gene, partialsequence.
HM133353	STB11	121	0	OXY107	Biosope_T60_16S_OXY107	21	stramenopiles	Dictyochophyceae			HM133342	FJ649290	99.47%	Uncultured phototrophic eukaryote clone STB1_25m_F8 16S ribosomal RNA gene,partial sequence; chloroplast.
HM133354	STB11	121	0	OXY107	Biosope_T60_16S_OXY107	22	Stramenopiles	Dictyochophyceae			HM133300	FJ826020	99.87%	Uncultured phototrophic eukaryote clone BM1-8-32 16S ribosomal RNA gene,partial sequence; plastid.
HM133355	STB11	121	0	OXY107	Biosope_T60_16S_OXY107	23	stramenopiles	Bolidophyceae			HM133355	FJ456835	99.35%	Uncultured bacterium clone A072_NCI 16S ribosomal RNA gene, completesequence.
HM133356	STB11	121	0	OXY107	Biosope_T60_16S_OXY107	26	Chlorophyta	Prasinophyceae	clade 16S-IX		HM133267	FJ649270	95.57%	Uncultured phototrophic eukaryote clone STB1_25m_C10 16S ribosomal RNAgene, partial sequence; chloroplast.
HM133357	STB11	121	0	OXY107	Biosope_T60_16S_OXY107	27	stramenopiles	Chrysophyceae			HM133149	EF052157	99.60%	Uncultured chrysophyte clone MC615-90 16S ribosomal RNA gene, partialsequence; plastid.
HM133358	STB11	121	0	OXY107	Biosope_T60_16S_OXY107	28	Stramenopiles	Dictyochophyceae			HM133342	FJ649290	98.67%	Uncultured phototrophic eukaryote clone STB1_25m_F8 16S ribosomal RNA gene,partial sequence; chloroplast.
HM133359	STB11	121	0	OXY107	Biosope_T60_16S_OXY107	30	stramenopiles	Chrysophyceae			HM133478	EF052159	99.60%	Uncultured chrysophyte clone MC615-92 16S ribosomal RNA gene, partialsequence; plastid.
HM133360	STB11	121	0	OXY107	Biosope_T60_16S_OXY107	31	stramenopiles	Chrysophyceae			HM133149	EF052157	99.87%	Uncultured chrysophyte clone MC615-90 16S ribosomal RNA gene, partialsequence; plastid.
HM133361	STB11	121	0	OXY107	Biosope_T60_16S_OXY107	33	Stramenopiles	Dictyochophyceae			HM133342	EF052136	99.60%	Uncultured dictyochophyte clone MC615-64 16S ribosomal RNA gene, partialsequence; plastid.
HM133362	STB11	121	0	PLA491	Biosope_T60_16S_PLA491	1	stramenopiles	Chrysophyceae			HM133478	EF052159	99.60%	Uncultured chrysophyte clone MC615-92 16S ribosomal RNA gene, partialsequence; plastid.
HM133363	STB11	121	0	PLA491	Biosope_T60_16S_PLA491	2	Stramenopiles	Pelagophyceae			HM133308	GQ231541	97.88%	Aureococcus anophagefferens strain CCMP 1984 chloroplast, complete genome.
HM133364	STB11	121	0	PLA491	Biosope_T60_16S_PLA491	3	Haptophyta	Prymnesiophyceae			HM133266	EF574443	99.47%	Uncultured bacterium clone S25_787 16S ribosomal RNA gene, partialsequence.
HM133365	STB11	121	0	PLA491	Biosope_T60_16S_PLA491	4	Haptophyta	Prymnesiophyceae			HM133343	FJ649286	97.76%	Uncultured phototrophic eukaryote clone STB1_25m_E12 16S ribosomal RNAgene, partial sequence; chloroplast.
HM133366	STB11	121	0	PLA491	Biosope_T60_16S_PLA491	5	Haptophyta	Prymnesiophyceae			HM133549	EF574491	99.47%	Uncultured bacterium clone S25_835 16S ribosomal RNA gene, partialsequence.
HM133367	STB11	121	0	PLA491	Biosope_T60_16S_PLA491	6	Haptophyta	Prymnesiophyceae			HM133343	FJ649286	97.75%	Uncultured phototrophic eukaryote clone STB1_25m_E12 16S ribosomal RNAgene, partial sequence; chloroplast.
HM133368	STB11	121	0	PLA491	Biosope_T60_16S_PLA491	8	Haptophyta	Prymnesiophyceae			HM133343	FJ649286	97.62%	Uncultured phototrophic eukaryote clone STB1_25m_E12 16S ribosomal RNAgene, partial sequence; chloroplast.
HM133369	STB11	121	0	PLA491	Biosope_T60_16S_PLA491	10	Haptophyta	Prymnesiophyceae			HM133266	EF574441	100.00%	Uncultured bacterium clone S25_785 16S ribosomal RNA gene, partialsequence.
HM133370	STB11	121	0	PLA491	Biosope_T60_16S_PLA491	11	Haptophyta	Prymnesiophyceae			HM133343	FJ649286	97.49%	Uncultured phototrophic eukaryote clone STB1_25m_E12 16S ribosomal RNAgene, partial sequence; chloroplast.
HM133371	STB11	121	0	PLA491	Biosope_T60_16S_PLA491	12	Haptophyta	Prymnesiophyceae			HM133266	EF573710	99.87%	Uncultured bacterium clone S25_54 16S ribosomal RNA gene, partial sequence.
HM133372	STB11	121	0	PLA491	Biosope_T60_16S_PLA491	13	Haptophyta	Prymnesiophyceae			HM133343	FJ649286	97.75%	Uncultured phototrophic eukaryote clone STB1_25m_E12 16S ribosomal RNAgene, partial sequence; chloroplast.
HM133373	STB11	121	0	PLA491	Biosope_T60_16S_PLA491	14	Haptophyta	Prymnesiophyceae			HM133486	EF052047	99.87%	Uncultured haptophyte clone 250304-50 16S ribosomal RNA gene, partialsequence; plastid.
HM133374	STB11	121	0	PLA491	Biosope_T60_16S_PLA491	15	Haptophyta	Prymnesiophyceae			HM133343	FJ649286	97.62%	Uncultured phototrophic eukaryote clone STB1_25m_E12 16S ribosomal RNAgene, partial sequence; chloroplast.
HM133375	STB11	121	0	PLA491	Biosope_T60_16S_PLA491	16	stramenopiles	Chrysophyceae			HM133149	EF052157	99.73%	Uncultured chrysophyte clone MC615-90 16S ribosomal RNA gene, partialsequence; plastid.
HM133376	STB11	121	0	PLA491	Biosope_T60_16S_PLA491	17	Haptophyta	Prymnesiophyceae			HM133343	FJ649286	97.62%	Uncultured phototrophic eukaryote clone STB1_25m_E12 16S ribosomal RNAgene, partial sequence; chloroplast.
HM133377	STB11	121	0	PLA491	Biosope_T60_16S_PLA491	18	Haptophyta	Prymnesiophyceae			HM133343	FJ649286	97.88%	Uncultured phototrophic eukaryote clone STB1_25m_E12 16S ribosomal RNAgene, partial sequence; chloroplast.
HM133378	STB11	121	0	PLA491	Biosope_T60_16S_PLA491	19	Chlorophyta	Prasinophyceae	clade 16S-IX		HM133244	DQ438491	92.80%	Uncultured bacterium clone ECS-P7-D55 16S ribosomal RNA gene, partialsequence.
HM133379	STB11	121	0	PLA491	Biosope_T60_16S_PLA491	20	stramenopiles	Dictyochophyceae			HM133342	EF052136	99.34%	Uncultured dictyochophyte clone MC615-64 16S ribosomal RNA gene, partialsequence; plastid.
HM133380	STB11	121	0	PLA491	Biosope_T60_16S_PLA491	21	Haptophyta	Prymnesiophyceae			HM133343	FJ649286	97.77%	Uncultured phototrophic eukaryote clone STB1_25m_E12 16S ribosomal RNAgene, partial sequence; chloroplast.
HM133381	STB11	121	0	PLA491	Biosope_T60_16S_PLA491	22	stramenopiles	Chrysophyceae			HM133470	EF574962	98.93%	Uncultured bacterium clone S25_1306 16S ribosomal RNA gene, partialsequence.
HM133382	STB11	121	0	PLA491	Biosope_T60_16S_PLA491	23	Haptophyta	Prymnesiophyceae			HM133549	EF052237	99.61%	Uncultured haptophyte clone MC622-77 16S ribosomal RNA gene, partialsequence; plastid.
HM133383	STB11	121	0	PLA491	Biosope_T60_16S_PLA491	24	stramenopiles	Chrysophyceae			HM133149	EF052157	99.73%	Uncultured chrysophyte clone MC615-90 16S ribosomal RNA gene, partialsequence; plastid.
HM133384	STB11	121	0	PLA491	Biosope_T60_16S_PLA491	25	Haptophyta	Prymnesiophyceae			HM133343	FJ649286	97.77%	Uncultured phototrophic eukaryote clone STB1_25m_E12 16S ribosomal RNAgene, partial sequence; chloroplast.
HM133385	STB11	121	0	PLA491	Biosope_T60_16S_PLA491	26	Stramenopiles	Dictyochophyceae			HM133342	FJ649290	99.46%	Uncultured phototrophic eukaryote clone STB1_25m_F8 16S ribosomal RNA gene,partial sequence; chloroplast.
HM133386	STB11	121	0	PLA491	Biosope_T60_16S_PLA491	27	stramenopiles	Chrysophyceae			HM133478	EF052159	98.65%	Uncultured chrysophyte clone MC615-92 16S ribosomal RNA gene, partialsequence; plastid.
HM133387	STB11	121	0	PLA491	Biosope_T60_16S_PLA491	28	stramenopiles	Chrysophyceae			HM133478	EF052159	99.47%	Uncultured chrysophyte clone MC615-92 16S ribosomal RNA gene, partialsequence; plastid.
HM133388	STB11	121	0	PLA491	Biosope_T60_16S_PLA491	30	stramenopiles	Chrysophyceae			HM133149	EF052157	99.87%	Uncultured chrysophyte clone MC615-90 16S ribosomal RNA gene, partialsequence; plastid.
HM133389	STB11	121	0	PLA491	Biosope_T60_16S_PLA491	31	stramenopiles	Chrysophyceae			HM133478	EF052159	99.59%	Uncultured chrysophyte clone MC615-92 16S ribosomal RNA gene, partialsequence; plastid.
HM133272	STB11	121	200	OXY107	Biosope_T58_16S_OXY107	25	Stramenopiles	Pelagophyceae			HM133308	FJ649273	99.61%	Uncultured phototrophic eukaryote clone STB1_25m_D2 16S ribosomal RNA gene,partial sequence; chloroplast.
HM133273	STB11	121	200	OXY107	Biosope_T58_16S_OXY107	26	Stramenopiles	Pelagophyceae			HM133308	FJ649273	99.87%	Uncultured phototrophic eukaryote clone STB1_25m_D2 16S ribosomal RNA gene,partial sequence; chloroplast.
HM133274	STB11	121	200	OXY107	Biosope_T58_16S_OXY107	27	Stramenopiles	Pelagophyceae			HM133308	FJ649273	99.35%	Uncultured phototrophic eukaryote clone STB1_25m_D2 16S ribosomal RNA gene,partial sequence; chloroplast.
HM133275	STB11	121	200	OXY107	Biosope_T58_16S_OXY107	28	Stramenopiles	Pelagophyceae			HM133308	FJ649273	99.47%	Uncultured phototrophic eukaryote clone STB1_25m_D2 16S ribosomal RNA gene,partial sequence; chloroplast.
HM133276	STB11	121	200	OXY107	Biosope_T58_16S_OXY107	30	Stramenopiles	Pelagophyceae			HM133308	FJ649273	99.34%	Uncultured phototrophic eukaryote clone STB1_25m_D2 16S ribosomal RNA gene,partial sequence; chloroplast.
HM133277	STB11	121	200	OXY107	Biosope_T58_16S_OXY107	31	Stramenopiles	Pelagophyceae			HM133308	DQ513100	99.61%	Uncultured bacterium clone FS274-44B-03 16S ribosomal RNA gene, partialsequence.
HM133278	STB11	121	200	OXY107	Biosope_T58_16S_OXY107	32	Stramenopiles	Pelagophyceae			HM133308	FJ649273	99.47%	Uncultured phototrophic eukaryote clone STB1_25m_D2 16S ribosomal RNA gene,partial sequence; chloroplast.
HM133279	STB11	121	200	OXY107	Biosope_T58_16S_OXY107	33	Stramenopiles	Pelagophyceae			HM133308	FJ649273	99.74%	Uncultured phototrophic eukaryote clone STB1_25m_D2 16S ribosomal RNA gene,partial sequence; chloroplast.
HM133280	STB11	121	200	OXY107	Biosope_T58_16S_OXY107	34	Stramenopiles	Pelagophyceae			HM133308	FJ649273	100.00%	Uncultured phototrophic eukaryote clone STB1_25m_D2 16S ribosomal RNA gene,partial sequence; chloroplast.
HM133281	STB11	121	200	OXY107	Biosope_T58_16S_OXY107	35	Stramenopiles	Pelagophyceae			HM133308	FJ649273	99.61%	Uncultured phototrophic eukaryote clone STB1_25m_D2 16S ribosomal RNA gene,partial sequence; chloroplast.
HM133282	STB11	121	200	OXY107	Biosope_T58_16S_OXY107	36	Stramenopiles	Pelagophyceae			HM133308	FJ649273	99.87%	Uncultured phototrophic eukaryote clone STB1_25m_D2 16S ribosomal RNA gene,partial sequence; chloroplast.
HM133283	STB11	121	200	OXY107	Biosope_T58_16S_OXY107	37	Stramenopiles	Pelagophyceae			HM133308	FJ649273	99.61%	Uncultured phototrophic eukaryote clone STB1_25m_D2 16S ribosomal RNA gene,partial sequence; chloroplast.
HM133284	STB11	121	200	OXY107	Biosope_T58_16S_OXY107	39	Stramenopiles	Pelagophyceae			HM133308	FJ649273	99.74%	Uncultured phototrophic eukaryote clone STB1_25m_D2 16S ribosomal RNA gene,partial sequence; chloroplast.

Accession	station	CTD cast	depth	primer_forward	Clone_library	Clone	Division	Class	Order	Genus	OTU	BLAST Accession	BLAST % ID	BLAST Description
HM133285	STB11	121	200	OXY107	Biosope_T58_16S_OXY107	40	stramenopiles	Dictyochophyceae			HM133285	EF051939	96.99%	Uncultured dictyochophyte clone MAMA64-21 16S ribosomal RNA gene, partialsequence; plastid.
HM133286	STB11	121	200	OXY107	Biosope_T58_16S_OXY107	41	Stramenopiles	Pelagophyceae			HM133308	FJ649273	99.61%	Uncultured phototrophic eukaryote clone STB1_25m_D2 16S ribosomal RNA gene,partial sequence; chloroplast.
HM133287	STB11	121	200	OXY107	Biosope_T58_16S_OXY107	42	Stramenopiles	Pelagophyceae			HM133308	FJ649273	99.61%	Uncultured phototrophic eukaryote clone STB1_25m_D2 16S ribosomal RNA gene,partial sequence; chloroplast.
HM133288	STB11	121	200	OXY107	Biosope_T58_16S_OXY107	43	Stramenopiles	Pelagophyceae			HM133308	FJ649273	99.74%	Uncultured phototrophic eukaryote clone STB1_25m_D2 16S ribosomal RNA gene,partial sequence; chloroplast.
HM133289	STB11	121	200	OXY107	Biosope_T58_16S_OXY107	44	Stramenopiles	Pelagophyceae			HM133308	FJ649273	99.87%	Uncultured phototrophic eukaryote clone STB1_25m_D2 16S ribosomal RNA gene,partial sequence; chloroplast.
HM133290	STB11	121	200	OXY107	Biosope_T58_16S_OXY107	46	Stramenopiles	Pelagophyceae			HM133308	FJ649273	99.74%	Uncultured phototrophic eukaryote clone STB1_25m_D2 16S ribosomal RNA gene,partial sequence; chloroplast.
HM133291	STB11	121	200	OXY107	Biosope_T58_16S_OXY107	47	Stramenopiles	Pelagophyceae			HM133308	FJ649273	99.61%	Uncultured phototrophic eukaryote clone STB1_25m_D2 16S ribosomal RNA gene,partial sequence; chloroplast.
HM133292	STB11	121	200	OXY107	Biosope_T58_16S_OXY107	48	Stramenopiles	Pelagophyceae			HM133308	FJ649273	99.61%	Uncultured phototrophic eukaryote clone STB1_25m_D2 16S ribosomal RNA gene,partial sequence; chloroplast.
HM133293	STB11	121	200	OXY107	Biosope_T58_16S_OXY107	49	Stramenopiles	Pelagophyceae			HM133308	FJ649273	99.74%	Uncultured phototrophic eukaryote clone STB1_25m_D2 16S ribosomal RNA gene,partial sequence; chloroplast.
HM133294	STB11	121	200	OXY107	Biosope_T58_16S_OXY107	65	Stramenopiles	Pelagophyceae			HM133308	FJ649273	100.00%	Uncultured phototrophic eukaryote clone STB1_25m_D2 16S ribosomal RNA gene,partial sequence; chloroplast.
HM133295	STB11	121	200	OXY107	Biosope_T58_16S_OXY107	66	Stramenopiles	Pelagophyceae			HM133308	FJ649273	99.74%	Uncultured phototrophic eukaryote clone STB1_25m_D2 16S ribosomal RNA gene,partial sequence; chloroplast.
HM133296	STB11	121	200	OXY107	Biosope_T58_16S_OXY107	67	Stramenopiles	Pelagophyceae			HM133308	FJ649273	99.47%	Uncultured phototrophic eukaryote clone STB1_25m_D2 16S ribosomal RNA gene,partial sequence; chloroplast.
HM133297	STB11	121	200	OXY107	Biosope_T58_16S_OXY107	68	Stramenopiles	Pelagophyceae			HM133308	FJ649273	98.95%	Uncultured phototrophic eukaryote clone STB1_25m_D2 16S ribosomal RNA gene,partial sequence; chloroplast.
HM133298	STB11	121	200	OXY107	Biosope_T58_16S_OXY107	69	stramenopiles	Dictyochophyceae			HM133298	EF051939	93.67%	Uncultured dictyochophyte clone MAMA64-21 16S ribosomal RNA gene, partialsequence; plastid.
HM133299	STB11	121	200	OXY107	Biosope_T58_16S_OXY107	70	Stramenopiles	Pelagophyceae			HM133308	FJ649273	99.87%	Uncultured phototrophic eukaryote clone STB1_25m_D2 16S ribosomal RNA gene,partial sequence; chloroplast.
HM133300	STB11	121	200	OXY107	Biosope_T58_16S_OXY107	71	Stramenopiles	Dictyochophyceae			HM133300	FJ826020	99.74%	Uncultured phototrophic eukaryote clone BM1-8-32 16S ribosomal RNA gene,partial sequence; plastid.
HM133301	STB11	121	200	OXY107	Biosope_T58_16S_OXY107	72	Stramenopiles	Pelagophyceae			HM133308	FJ649273	99.74%	Uncultured phototrophic eukaryote clone STB1_25m_D2 16S ribosomal RNA gene,partial sequence; chloroplast.
HM133302	STB11	121	200	OXY107	Biosope_T58_16S_OXY107	73	Stramenopiles	Pelagophyceae			HM133308	FJ649273	99.74%	Uncultured phototrophic eukaryote clone STB1_25m_D2 16S ribosomal RNA gene,partial sequence; chloroplast.
HM133303	STB11	121	200	OXY107	Biosope_T58_16S_OXY107	74	Stramenopiles	Pelagophyceae			HM133308	DQ513100	99.74%	Uncultured bacterium clone FS274-44B-03 16S ribosomal RNA gene, partialsequence.
HM133304	STB11	121	200	OXY107	Biosope_T58_16S_OXY107	75	Stramenopiles	Pelagophyceae			HM133308	FJ649273	99.61%	Uncultured phototrophic eukaryote clone STB1_25m_D2 16S ribosomal RNA gene,partial sequence; chloroplast.
HM133305	STB11	121	200	OXY107	Biosope_T58_16S_OXY107	76	Stramenopiles	Pelagophyceae			HM133308	FJ649273	99.61%	Uncultured phototrophic eukaryote clone STB1_25m_D2 16S ribosomal RNA gene,partial sequence; chloroplast.
HM133306	STB11	121	200	OXY107	Biosope_T58_16S_OXY107	77	Stramenopiles	Pelagophyceae			HM133308	DQ513100	99.61%	Uncultured bacterium clone FS274-44B-03 16S ribosomal RNA gene, partialsequence.
HM133307	STB11	121	200	OXY107	Biosope_T58_16S_OXY107	78	Stramenopiles	Pelagophyceae			HM133308	DQ513100	99.87%	Uncultured bacterium clone FS274-44B-03 16S ribosomal RNA gene, partialsequence.
HM133308	STB11	121	200	OXY107	Biosope_T58_16S_OXY107	79	Stramenopiles	Pelagophyceae			HM133308	DQ513100	99.74%	Uncultured bacterium clone FS274-44B-03 16S ribosomal RNA gene, partialsequence.
HM133309	STB11	121	200	OXY107	Biosope_T58_16S_OXY107	80	Stramenopiles	Pelagophyceae			HM133308	DQ513100	99.74%	Uncultured bacterium clone FS274-44B-03 16S ribosomal RNA gene, partialsequence.
HM133310	STB11	121	200	OXY107	Biosope_T58_16S_OXY107	81	Stramenopiles	Pelagophyceae			HM133308	DQ513100	99.74%	Uncultured bacterium clone FS274-44B-03 16S ribosomal RNA gene, partialsequence.
HM133311	STB11	121	200	OXY107	Biosope_T58_16S_OXY107	82	Stramenopiles	Dictyochophyceae			HM133300	FJ826020	98.03%	Uncultured phototrophic eukaryote clone BM1-8-32 16S ribosomal RNA gene,partial sequence; plastid.
HM133312	STB11	121	200	OXY107	Biosope_T58_16S_OXY107	83	Stramenopiles	Pelagophyceae			HM133308	FJ649273	99.34%	Uncultured phototrophic eukaryote clone STB1_25m_D2 16S ribosomal RNA gene,partial sequence; chloroplast.
HM133313	STB11	121	200	OXY107	Biosope_T58_16S_OXY107	85	Stramenopiles	Pelagophyceae			HM133308	FJ649273	99.60%	Uncultured phototrophic eukaryote clone STB1_25m_D2 16S ribosomal RNA gene,partial sequence; chloroplast.
HM133314	STB11	121	200	OXY107	Biosope_T58_16S_OXY107	86	Stramenopiles	Pelagophyceae			HM133308	DQ513100	99.87%	Uncultured bacterium clone FS274-44B-03 16S ribosomal RNA gene, partialsequence.
HM133315	STB11	121	200	OXY107	Biosope_T58_16S_OXY107	87	Stramenopiles	Pelagophyceae			HM133308	DQ513100	99.61%	Uncultured bacterium clone FS274-44B-03 16S ribosomal RNA gene, partialsequence.
HM133316	STB11	121	200	OXY107	Biosope_T58_16S_OXY107	88	Stramenopiles	Pelagophyceae			HM133308	GQ231541	97.91%	Aureococcus anophagefferens strain CCMP 1984 chloroplast, complete genome.
HM133317	STB11	121	200	PLA491	Biosope_T58_16S_PLA491	1	Haptophyta	Prymnesiophyceae			HM133317	X65101	97.52%	Ochrosphaera sp. 181 plastid RNA for 16S like rRNA small subunit
HM133318	STB11	121	200	PLA491	Biosope_T58_16S_PLA491	2	Stramenopiles	Pelagophyceae			HM133308	FJ649273	99.28%	Uncultured phototrophic eukaryote clone STB1_25m_D2 16S ribosomal RNA gene,partial sequence; chloroplast.
HM133319	STB11	121	200	PLA491	Biosope_T58_16S_PLA491	3	Haptophyta	Prymnesiophyceae			HM133266	EF574945	99.87%	Uncultured bacterium clone S25_1289 16S ribosomal RNA gene, partialsequence.
HM133320	STB11	121	200	PLA491	Biosope_T58_16S_PLA491	4	Haptophyta	Prymnesiophyceae			HM133266	EF052003	100.00%	Uncultured haptophyte clone 250304-3 16S ribosomal RNA gene, partialsequence; plastid.
HM133321	STB11	121	200	PLA491	Biosope_T58_16S_PLA491	5	Haptophyta	Prymnesiophyceae			HM133266	EF574945	100.00%	Uncultured bacterium clone S25_1289 16S ribosomal RNA gene, partialsequence.
HM133322	STB11	121	200	PLA491	Biosope_T58_16S_PLA491	6	Stramenopiles	Pelagophyceae			HM133308	FJ649273	99.60%	Uncultured phototrophic eukaryote clone STB1_25m_D2 16S ribosomal RNA gene,partial sequence; chloroplast.
HM133323	STB11	121	200	PLA491	Biosope_T58_16S_PLA491	9	Haptophyta	Prymnesiophyceae			HM133266	EF052003	99.74%	Uncultured haptophyte clone 250304-3 16S ribosomal RNA gene, partialsequence; plastid.
HM133324	STB11	121	200	PLA491	Biosope_T58_16S_PLA491	10	Haptophyta	Prymnesiophyceae			HM133266	EF052003	99.74%	Uncultured haptophyte clone 250304-3 16S ribosomal RNA gene, partialsequence; plastid.
HM133325	STB11	121	200	PLA491	Biosope_T58_16S_PLA491	11	Stramenopiles	Pelagophyceae			HM133308	FJ649273	99.74%	Uncultured phototrophic eukaryote clone STB1_25m_D2 16S ribosomal RNA gene,partial sequence; chloroplast.
HM133326	STB11	121	200	PLA491	Biosope_T58_16S_PLA491	12	Haptophyta	Prymnesiophyceae			HM133339	DQ395642	99.08%	Uncultured organism clone ctg_CGOF234 16S ribosomal RNA gene, partialsequence.
HM133327	STB11	121	200	PLA491	Biosope_T58_16S_PLA491	13	Stramenopiles	Pelagophyceae			HM133308	GQ231541	97.88%	Aureococcus anophagefferens strain CCMP 1984 chloroplast, complete genome.
HM133328	STB11	121	200	PLA491	Biosope_T58_16S_PLA491	14	Haptophyta	Prymnesiophyceae			HM133546	EF574745	99.61%	Uncultured bacterium clone S25_1089 16S ribosomal RNA gene, partialsequence.
HM133329	STB11	121	200	PLA491	Biosope_T58_16S_PLA491	15	Stramenopiles	Pelagophyceae			HM133308	FJ649273	99.53%	Uncultured phototrophic eukaryote clone STB1_25m_D2 16S ribosomal RNA gene,partial sequence; chloroplast.
HM133330	STB11	121	200	PLA491	Biosope_T58_16S_PLA491	17	Haptophyta	Prymnesiophyceae			HM133339	DQ395642	99.74%	Uncultured organism clone ctg_CGOF234 16S ribosomal RNA gene, partialsequence.
HM133331	STB11	121	200	PLA491	Biosope_T58_16S_PLA491	19	Haptophyta	Prymnesiophyceae			HM133331	FJ649283	93.23%	Uncultured phototrophic eukaryote clone STB1_25m_E9 16S ribosomal RNA gene,partial sequence; chloroplast.
HM133332	STB11	121	200	PLA491	Biosope_T58_16S_PLA491	21	Haptophyta	Prymnesiophyceae			HM133503	EU182078	98.82%	Uncultured bacterium clone D15_7_SW_G_83 16S ribosomal RNA gene, partialsequence.
HM133333	STB11	121	200	PLA491	Biosope_T58_16S_PLA491	23	Stramenopiles	Pelagophyceae			HM133308	FJ649273	99.08%	Uncultured phototrophic eukaryote clone STB1_25m_D2 16S ribosomal RNA gene,partial sequence; chloroplast.
HM133334	STB11	121	200	PLA491	Biosope_T58_16S_PLA491	33	Haptophyta	Prymnesiophyceae			HM133339	DQ395642	99.21%	Uncultured organism clone ctg_CGOF234 16S ribosomal RNA gene, partialsequence.
HM133335	STB11	121	200	PLA491	Biosope_T58_16S_PLA491	34	Haptophyta	Prymnesiophyceae			HM133546	EF574745	99.61%	Uncultured bacterium clone S25_1089 16S ribosomal RNA gene, partialsequence.
HM133336	STB11	121	200	PLA491	Biosope_T58_16S_PLA491	35	Haptophyta	Prymnesiophyceae			HM133339	DQ395642	99.47%	Uncultured organism clone ctg_CGOF234 16S ribosomal RNA gene, partialsequence.
HM133337	STB11	121	200	PLA491	Biosope_T58_16S_PLA491	36	Haptophyta	Prymnesiophyceae			HM133337	EF052069	99.61%	Uncultured haptophyte clone 250304-84 16S ribosomal RNA gene, partialsequence; plastid.
HM133338	STB11	121	200	PLA491	Biosope_T58_16S_PLA491	37	Stramenopiles	Pelagophyceae			HM133308	FJ649273	99.55%	Uncultured phototrophic eukaryote clone STB1_25m_D2 16S ribosomal RNA gene,partial sequence; chloroplast.
HM133339	STB11	121	200	PLA491	Biosope_T58_16S_PLA491	38	Haptophyta	Prymnesiophyceae			HM133339	DQ395642	99.87%	Uncultured organism clone ctg_CGOF234 16S ribosomal RNA gene, partialsequence.
HM133340	STB11	121	200	PLA491	Biosope_T58_16S_PLA491	40	Haptophyta	Prymnesiophyceae			HM133339	DQ395642	99.34%	Uncultured organism clone ctg_CGOF234 16S ribosomal RNA gene, partialsequence.
HM133390	STB12	125	40	PLA491	Biosope_T65_16S	10a	Haptophyta	Prymnesiophyceae			HM133343	FJ649286	97.93%	Uncultured phototrophic eukaryote clone STB1_25m_E12 16S ribosomal RNAgene, partial sequence; chloroplast.
HM133391	STB12	125	40	PLA491	Biosope_T65_16S	11a	Haptophyta	Prymnesiophyceae			HM133343	FJ649286	99.84%	Uncultured phototrophic eukaryote clone STB1_25m_E12 16S ribosomal RNAgene, partial sequence; chloroplast.
HM133392	STB12	125	40	PLA491	Biosope_T65_16S	11b	Haptophyta	Prymnesiophyceae			HM133343	FJ649286	99.06%	Uncultured phototrophic eukaryote clone STB1_25m_E12 16S ribosomal RNAgene, partial sequence; chloroplast.
HM133393	STB12	125	40	PLA491	Biosope_T65_16S	12a	Haptophyta	Prymnesiophyceae			HM133393	EF052216	95.44%	Uncultured haptophyte clone MC622-55 16S ribosomal RNA gene, partialsequence; plastid.
HM133394	STB12	125	40	PLA491	Biosope_T65_16S	12b	Haptophyta	Prymnesiophyceae			HM133486	EF052047	98.80%	Uncultured haptophyte clone 250304-50 16S ribosomal RNA gene, partialsequence; plastid.
HM133395	STB12	125	40	PLA491	Biosope_T65_16S	13a	Haptophyta	Prymnesiophyceae			HM133343	FJ649286	99.46%	Uncultured phototrophic eukaryote clone STB1_25m_E12 16S ribosomal RNAgene, partial sequence; chloroplast.
HM133396	STB12	125	40	PLA491	Biosope_T65_16S	17a	Haptophyta	Prymnesiophyceae			HM133343	FJ649287	97.55%	Uncultured phototrophic eukaryote clone STB1_25m_F1 16S ribosomal RNA gene,partial sequence; chloroplast.
HM133397	STB12	125	40	PLA491	Biosope_T65_16S	18a	Haptophyta	Prymnesiophyceae			HM133065	EU805127	99.73%	Uncultured bacterium clone 6C233116 16S ribosomal RNA gene, partialsequence.
HM133398	STB12	125	40	PLA491	Biosope_T65_16S	1b	Haptophyta	Prymnesiophyceae			HM133179	EF052033	96.87%	Uncultured haptophyte clone 250304-35 16S ribosomal RNA gene, partialsequence; plastid.
HM133399	STB12	125	40	PLA491	Biosope_T65_16S	22a	Haptophyta	Prymnesiophyceae			HM133343	FJ649286	98.23%	Uncultured phototrophic eukaryote clone STB1_25m_E12 16S ribosomal RNAgene, partial sequence; chloroplast.
HM133400	STB12	125	40	PLA491	Biosope_T65_16S	23a	Haptophyta	Prymnesiophyceae			HM133343	AB199887	94.05%	Dinophysis mitra chloroplast gene for 16S rRNA, partial sequence,clone:DM5.

Accession	station	CTD cast	depth	primer_forward	Clone_library	Clone	Division	Class	Order	Genus	OTU	BLAST Accession	BLAST % ID	BLAST Description
HM133401	STB12	125	40	PLA491	Biosope_T65_16S	25a	Haptophyta	Prymnesiophyceae			HM133503	AF001655	98.26%	Environmental clone OCS31 small subunit ribosomal RNA gene, chloroplastgene for chloroplast RNA, complete sequence.
HM133402	STB12	125	40	PLA491	Biosope_T65_16S	25b	Haptophyta	Prymnesiophyceae			HM133402	EF052229	92.72%	Uncultured haptophyte clone MC622-69 16S ribosomal RNA gene, partialsequence; plastid.
HM133403	STB12	125	40	PLA491	Biosope_T65_16S	26a	Haptophyta	Prymnesiophyceae			HM133403	EF051979	96.81%	Uncultured haptophyte clone MAMA64-117 16S ribosomal RNA gene, partialsequence; plastid.
HM133404	STB12	125	40	PLA491	Biosope_T65_16S	27a	Haptophyta	Prymnesiophyceae			HM133266	EF052028	95.91%	Uncultured haptophyte clone 250304-29 16S ribosomal RNA gene, partialsequence; plastid.
HM133405	STB12	125	40	PLA491	Biosope_T65_16S	27b	Haptophyta	Prymnesiophyceae			HM133343	FJ649286	97.49%	Uncultured phototrophic eukaryote clone STB1_25m_E12 16S ribosomal RNAgene, partial sequence; chloroplast.
HM133406	STB12	125	40	PLA491	Biosope_T65_16S	28a	Haptophyta	Prymnesiophyceae			HM133343	FJ649286	97.91%	Uncultured phototrophic eukaryote clone STB1_25m_E12 16S ribosomal RNAgene, partial sequence; chloroplast.
HM133407	STB12	125	40	PLA491	Biosope_T65_16S	2a	Haptophyta	Prymnesiophyceae			HM133407	EU188193	92.61%	Uncultured marine microorganism clone 4041AA86 16S ribosomal RNA gene,partial sequence.
HM133408	STB12	125	40	PLA491	Biosope_T65_16S	2b	Haptophyta	Prymnesiophyceae			HM133343	FJ649286	99.53%	Uncultured phototrophic eukaryote clone STB1_25m_E12 16S ribosomal RNAgene, partial sequence; chloroplast.
HM133409	STB12	125	40	PLA491	Biosope_T65_16S	30a	Haptophyta	Prymnesiophyceae			HM133343	EF574253	97.92%	Uncultured bacterium clone S25_597 16S ribosomal RNA gene, partialsequence.
HM133410	STB12	125	40	PLA491	Biosope_T65_16S	30b	Haptophyta	Prymnesiophyceae			HM133343	FJ649286	97.00%	Uncultured phototrophic eukaryote clone STB1_25m_E12 16S ribosomal RNAgene, partial sequence; chloroplast.
HM133411	STB12	125	40	PLA491	Biosope_T65_16S	31a	Haptophyta	Prymnesiophyceae			HM133343	FJ649286	98.77%	Uncultured phototrophic eukaryote clone STB1_25m_E12 16S ribosomal RNAgene, partial sequence; chloroplast.
HM133412	STB12	125	40	PLA491	Biosope_T65_16S	31b	Haptophyta	Prymnesiophyceae			HM133412	EF574319	97.32%	Uncultured bacterium clone S25_663 16S ribosomal RNA gene, partialsequence.
HM133413	STB12	125	40	PLA491	Biosope_T65_16S	32a	Haptophyta	Prymnesiophyceae			HM133343	FJ649286	97.85%	Uncultured phototrophic eukaryote clone STB1_25m_E12 16S ribosomal RNAgene, partial sequence; chloroplast.
HM133414	STB12	125	40	PLA491	Biosope_T65_16S	32b	Haptophyta	Prymnesiophyceae			HM133343	FJ649286	98.65%	Uncultured phototrophic eukaryote clone STB1_25m_E12 16S ribosomal RNAgene, partial sequence; chloroplast.
HM133415	STB12	125	40	PLA491	Biosope_T65_16S	33a	Haptophyta	Prymnesiophyceae			HM133439	FJ649274	97.38%	Uncultured phototrophic eukaryote clone STB1_25m_D3 16S ribosomal RNA gene,partial sequence; chloroplast.
HM133416	STB12	125	40	PLA491	Biosope_T65_16S	33b	Stramenopiles	Dictyochophyceae			HM133416	FJ649290	93.64%	Uncultured phototrophic eukaryote clone STB1_25m_F8 16S ribosomal RNA gene,partial sequence; chloroplast.
HM133417	STB12	125	40	PLA491	Biosope_T65_16S	35b	Haptophyta	Prymnesiophyceae			HM133266	EF052236	99.32%	Uncultured haptophyte clone MC622-76 16S ribosomal RNA gene, partialsequence; plastid.
HM133418	STB12	125	40	PLA491	Biosope_T65_16S	36a	Haptophyta	Prymnesiophyceae			HM133343	FJ649286	98.65%	Uncultured phototrophic eukaryote clone STB1_25m_E12 16S ribosomal RNAgene, partial sequence; chloroplast.
HM133419	STB12	125	40	PLA491	Biosope_T65_16S	37a	Haptophyta	Prymnesiophyceae			HM133419	EF574836	95.32%	Uncultured bacterium clone S25_1180 16S ribosomal RNA gene, partialsequence.
HM133420	STB12	125	40	PLA491	Biosope_T65_16S	38a	Haptophyta	Prymnesiophyceae			HM133343	FJ649286	98.68%	Uncultured phototrophic eukaryote clone STB1_25m_E12 16S ribosomal RNAgene, partial sequence; chloroplast.
HM133421	STB12	125	40	PLA491	Biosope_T65_16S	38b	Haptophyta	Prymnesiophyceae			HM133179	FJ745122	95.85%	Uncultured bacterium clone SHWN_night2_16S_634 16S ribosomal RNA gene,partial sequence.
HM133422	STB12	125	40	PLA491	Biosope_T65_16S	3a	Haptophyta	Prymnesiophyceae			HM133266	EF052022	97.40%	Uncultured haptophyte clone 250304-22 16S ribosomal RNA gene, partialsequence; plastid.
HM133423	STB12	125	40	PLA491	Biosope_T65_16S	3b	Haptophyta	Prymnesiophyceae			HM133549	EF574491	98.62%	Uncultured bacterium clone S25_835 16S ribosomal RNA gene, partialsequence.
HM133424	STB12	125	40	PLA491	Biosope_T65_16S	40a	Haptophyta	Prymnesiophyceae			HM133343	AB199887	95.54%	Dinophysis mitra chloroplast gene for 16S rRNA, partial sequence,clone:DM5.
HM133425	STB12	125	40	PLA491	Biosope_T65_16S	41a	Haptophyta	Prymnesiophyceae			HM133343	FJ649286	98.49%	Uncultured phototrophic eukaryote clone STB1_25m_E12 16S ribosomal RNAgene, partial sequence; chloroplast.
HM133426	STB12	125	40	PLA491	Biosope_T65_16S	41b	Haptophyta	Prymnesiophyceae			HM133426	EF574408	94.20%	Uncultured bacterium clone S25_752 16S ribosomal RNA gene, partialsequence.
HM133427	STB12	125	40	PLA491	Biosope_T65_16S	42a	stramenopiles	Chrysophyceae			HM133478	EF052159	95.94%	Uncultured chrysophyte clone MC615-92 16S ribosomal RNA gene, partialsequence; plastid.
HM133428	STB12	125	40	PLA491	Biosope_T65_16S	42b	Haptophyta	Prymnesiophyceae			HM133343	FJ649286	96.97%	Uncultured phototrophic eukaryote clone STB1_25m_E12 16S ribosomal RNAgene, partial sequence; chloroplast.
HM133429	STB12	125	40	PLA491	Biosope_T65_16S	44b	Haptophyta	Prymnesiophyceae			HM133179	EF052028	96.74%	Uncultured haptophyte clone 250304-29 16S ribosomal RNA gene, partialsequence; plastid.
HM133430	STB12	125	40	PLA491	Biosope_T65_16S	45a	Haptophyta	Prymnesiophyceae			HM133343	FJ649286	99.69%	Uncultured phototrophic eukaryote clone STB1_25m_E12 16S ribosomal RNAgene, partial sequence; chloroplast.
HM133431	STB12	125	40	PLA491	Biosope_T65_16S	45b	Haptophyta	Prymnesiophyceae			HM133431	FJ649286	96.86%	Uncultured phototrophic eukaryote clone STB1_25m_E12 16S ribosomal RNAgene, partial sequence; chloroplast.
HM133432	STB12	125	40	PLA491	Biosope_T65_16S	46a	Haptophyta	Prymnesiophyceae			HM133343	FJ649286	99.43%	Uncultured phototrophic eukaryote clone STB1_25m_E12 16S ribosomal RNAgene, partial sequence; chloroplast.
HM133433	STB12	125	40	PLA491	Biosope_T65_16S	46b	Haptophyta	Prymnesiophyceae			HM133419	EF574945	96.79%	Uncultured bacterium clone S25_1289 16S ribosomal RNA gene, partialsequence.
HM133434	STB12	125	40	PLA491	Biosope_T65_16S	48a	Haptophyta	Prymnesiophyceae			HM133343	FJ649286	99.04%	Uncultured phototrophic eukaryote clone STB1_25m_E12 16S ribosomal RNAgene, partial sequence; chloroplast.
HM133435	STB12	125	40	PLA491	Biosope_T65_16S	4b	Haptophyta	Prymnesiophyceae			HM133343	FJ649286	98.85%	Uncultured phototrophic eukaryote clone STB1_25m_E12 16S ribosomal RNAgene, partial sequence; chloroplast.
HM133436	STB12	125	40	PLA491	Biosope_T65_16S	50a	Haptophyta	Prymnesiophyceae			HM133266	EF574253	97.65%	Uncultured bacterium clone S25_597 16S ribosomal RNA gene, partialsequence.
HM133437	STB12	125	40	PLA491	Biosope_T65_16S	50b	Haptophyta	Prymnesiophyceae			HM133343	FJ649286	97.17%	Uncultured phototrophic eukaryote clone STB1_25m_E12 16S ribosomal RNAgene, partial sequence; chloroplast.
HM133438	STB12	125	40	PLA491	Biosope_T65_16S	51a	Haptophyta	Prymnesiophyceae			HM133266	EF574441	98.14%	Uncultured bacterium clone S25_785 16S ribosomal RNA gene, partialsequence.
HM133439	STB12	125	40	PLA491	Biosope_T65_16S	51b	Haptophyta	Prymnesiophyceae			HM133439	FJ649287	96.90%	Uncultured phototrophic eukaryote clone STB1_25m_F1 16S ribosomal RNA gene,partial sequence; chloroplast.
HM133440	STB12	125	40	PLA491	Biosope_T65_16S	52a	Haptophyta	Prymnesiophyceae			HM133343	FJ649286	98.76%	Uncultured phototrophic eukaryote clone STB1_25m_E12 16S ribosomal RNAgene, partial sequence; chloroplast.
HM133441	STB12	125	40	PLA491	Biosope_T65_16S	53a	Haptophyta	Prymnesiophyceae			HM133441	FJ649286	94.65%	Uncultured phototrophic eukaryote clone STB1_25m_E12 16S ribosomal RNAgene, partial sequence; chloroplast.
HM133442	STB12	125	40	PLA491	Biosope_T65_16S	54a	Haptophyta	Prymnesiophyceae			HM133343	FJ649286	97.75%	Uncultured phototrophic eukaryote clone STB1_25m_E12 16S ribosomal RNAgene, partial sequence; chloroplast.
HM133443	STB12	125	40	PLA491	Biosope_T65_16S	56a	Haptophyta	Prymnesiophyceae			HM133443	EF052249	92.40%	Uncultured haptophyte clone MC622-91 16S ribosomal RNA gene, partialsequence; plastid.
HM133444	STB12	125	40	PLA491	Biosope_T65_16S	57a	Haptophyta	Prymnesiophyceae			HM133179	FJ826213	96.20%	Uncultured marine bacterium clone A6-1-5 16S ribosomal RNA gene, partialsequence.
HM133445	STB12	125	40	PLA491	Biosope_T65_16S	58a	Haptophyta	Prymnesiophyceae			HM133445	EF052237	95.78%	Uncultured haptophyte clone MC622-77 16S ribosomal RNA gene, partialsequence; plastid.
HM133446	STB12	125	40	PLA491	Biosope_T65_16S	59a	Haptophyta	Prymnesiophyceae			HM133343	FJ649286	97.32%	Uncultured phototrophic eukaryote clone STB1_25m_E12 16S ribosomal RNAgene, partial sequence; chloroplast.
HM133447	STB12	125	40	PLA491	Biosope_T65_16S	5b	Haptophyta	Prymnesiophyceae			HM133266	EF052022	96.55%	Uncultured haptophyte clone 250304-22 16S ribosomal RNA gene, partialsequence; plastid.
HM133448	STB12	125	40	PLA491	Biosope_T65_16S	6b	Haptophyta	Prymnesiophyceae			HM133343	FJ649286	97.56%	Uncultured phototrophic eukaryote clone STB1_25m_E12 16S ribosomal RNAgene, partial sequence; chloroplast.
HM133449	STB12	125	40	PLA491	Biosope_T65_16S	7b	Haptophyta	Prymnesiophyceae			HM133449	EF051931	89.99%	Uncultured cryptophyte clone MC601-141 16S ribosomal RNA gene, partialsequence; plastid.
HM133450	STB12	125	40	PLA491	Biosope_T65_16S	8b	Haptophyta	Prymnesiophyceae			HM133266	EF052162	97.67%	Uncultured haptophyte clone MC615-95 16S ribosomal RNA gene, partialsequence; plastid.
HM133451	STB12	125	40	PLA491	Biosope_T65_16S	9a	Haptophyta	Prymnesiophyceae			HM133503	EF051979	98.71%	Uncultured haptophyte clone MAMA64-117 16S ribosomal RNA gene, partialsequence; plastid.
HM133452	STA14	132	5	OXY107	Biosope_T84_16S_OXY107	2	stramenopiles	Dictyochophyceae			HM133342	EF052136	99.34%	Uncultured dictyochophyte clone MC615-64 16S ribosomal RNA gene, partialsequence; plastid.
HM133453	STA14	132	5	OXY107	Biosope_T84_16S_OXY107	4	stramenopiles	Chrysophyceae			HM133470	EF574962	98.68%	Uncultured bacterium clone S25_1306 16S ribosomal RNA gene, partialsequence.
HM133454	STA14	132	5	OXY107	Biosope_T84_16S_OXY107	5	stramenopiles	Dictyochophyceae			HM133342	EF052136	99.61%	Uncultured dictyochophyte clone MC615-64 16S ribosomal RNA gene, partialsequence; plastid.
HM133455	STA14	132	5	OXY107	Biosope_T84_16S_OXY107	7	stramenopiles	Chrysophyceae			HM133253	EU182057	98.28%	Uncultured bacterium clone D15_7_SW_G_30 16S ribosomal RNA gene, partialsequence.
HM133456	STA14	132	5	OXY107	Biosope_T84_16S_OXY107	10	stramenopiles	Chrysophyceae			HM133470	EF574962	98.94%	Uncultured bacterium clone S25_1306 16S ribosomal RNA gene, partialsequence.
HM133457	STA14	132	5	OXY107	Biosope_T84_16S_OXY107	13	stramenopiles	Chrysophyceae			HM133470	EF574962	98.80%	Uncultured bacterium clone S25_1306 16S ribosomal RNA gene, partialsequence.
HM133458	STA14	132	5	OXY107	Biosope_T84_16S_OXY107	14	Stramenopiles	Pelagophyceae			HM133308	DQ513100	99.74%	Uncultured bacterium clone FS274-44B-03 16S ribosomal RNA gene, partialsequence.
HM133459	STA14	132	5	OXY107	Biosope_T84_16S_OXY107	43	stramenopiles	Chrysophyceae			HM133470	EF574962	98.94%	Uncultured bacterium clone S25_1306 16S ribosomal RNA gene, partialsequence.
HM133460	STA14	132	5	OXY107	Biosope_T84_16S_OXY107	44	Stramenopiles	Pelagophyceae			HM133308	DQ513100	99.48%	Uncultured bacterium clone FS274-44B-03 16S ribosomal RNA gene, partialsequence.
HM133461	STA14	132	5	OXY107	Biosope_T84_16S_OXY107	45	Stramenopiles	Pelagophyceae			HM133308	FJ649273	99.74%	Uncultured phototrophic eukaryote clone STB1_25m_D2 16S ribosomal RNA gene,partial sequence; chloroplast.
HM133462	STA14	132	5	OXY107	Biosope_T84_16S_OXY107	47	Stramenopiles	Dictyochophyceae			HM133342	FJ649290	98.43%	Uncultured phototrophic eukaryote clone STB1_25m_F8 16S ribosomal RNA gene,partial sequence; chloroplast.
HM133463	STA14	132	5	OXY107	Biosope_T84_16S_OXY107	59	Stramenopiles	Pelagophyceae			HM133308	FJ649273	99.87%	Uncultured phototrophic eukaryote clone STB1_25m_D2 16S ribosomal RNA gene,partial sequence; chloroplast.
HM133464	STA14	132	5	OXY107	Biosope_T84_16S_OXY107	61	Stramenopiles	Pelagophyceae			HM133308	FJ649273	99.34%	Uncultured phototrophic eukaryote clone STB1_25m_D2 16S ribosomal RNA gene,partial sequence; chloroplast.
HM133465	STA14	132	5	OXY107	Biosope_T84_16S_OXY107	63	Stramenopiles	Pelagophyceae			HM133308	FJ649273	99.61%	Uncultured phototrophic eukaryote clone STB1_25m_D2 16S ribosomal RNA gene,partial sequence; chloroplast.
HM133466	STA14	132	5	OXY107	Biosope_T84_16S_OXY107	64	Stramenopiles	Pelagophyceae			HM133308	FJ649273	99.85%	Uncultured phototrophic eukaryote clone STB1_25m_D2 16S ribosomal RNA gene,partial sequence; chloroplast.
HM133467	STA14	132	5	OXY107	Biosope_T84_16S_OXY107	66	stramenopiles	Chrysophyceae			HM133470	EF574962	98.68%	Uncultured bacterium clone S25_1306 16S ribosomal RNA gene, partialsequence.

Accession	station	CTD cast	depth	primer_forward	Clone_library	Clone	Division	Class	Order	Genus	OTU	BLAST Accession	BLAST % ID	BLAST Description
HM133468	STA14	132	5	OXY107	Biosope_T84_16S_OXY107	69	stramenopiles	Bolidophyceae		Bolidomonas	HM133355	AY702144	98.68%	Bolidomonas mediterranea strain RCC238 16S ribosomal RNA gene, partialsequence; plastid.
HM133469	STA14	132	5	OXY107	Biosope_T84_16S_OXY107	72	Stramenopiles	Pelagophyceae			HM133308	FJ649273	99.74%	Uncultured phototrophic eukaryote clone STB1_25m_D2 16S ribosomal RNA gene,partial sequence; chloroplast.
HM133470	STA14	132	5	OXY107	Biosope_T84_16S_OXY107	73	Stramenopiles	Chrysophyceae			HM133470	FJ649291	98.82%	Uncultured phototrophic eukaryote clone STB1_25m_F10 16S ribosomal RNAgene, partial sequence; chloroplast.
HM133471	STA14	132	5	OXY107	Biosope_T84_16S_OXY107	74	Chlorophyta	Prasinophyceae	clade 16S-IX		HM133471	FJ649270	99.61%	Uncultured phototrophic eukaryote clone STB1_25m_C10 16S ribosomal RNAgene, partial sequence; chloroplast.
HM133472	STA14	132	5	OXY107	Biosope_T84_16S_OXY107	75	Stramenopiles	Pelagophyceae			HM133308	FJ649273	99.87%	Uncultured phototrophic eukaryote clone STB1_25m_D2 16S ribosomal RNA gene,partial sequence; chloroplast.
HM133473	STA14	132	5	OXY107	Biosope_T84_16S_OXY107	77	Stramenopiles	Dictyochophyceae			HM133342	FJ649290	98.42%	Uncultured phototrophic eukaryote clone STB1_25m_F8 16S ribosomal RNA gene,partial sequence; chloroplast.
HM133474	STA14	132	5	OXY107	Biosope_T84_16S_OXY107	78	Stramenopiles	Pelagophyceae			HM133308	FJ649273	99.60%	Uncultured phototrophic eukaryote clone STB1_25m_D2 16S ribosomal RNA gene,partial sequence; chloroplast.
HM133475	STA14	132	5	OXY107	Biosope_T84_16S_OXY107	79	Stramenopiles	Pelagophyceae			HM133308	DQ513100	99.74%	Uncultured bacterium clone FS274-44B-03 16S ribosomal RNA gene, partialsequence.
HM133476	STA14	132	5	OXY107	Biosope_T84_16S_OXY107	80	stramenopiles	Chrysophyceae			HM133253	EU182057	98.54%	Uncultured bacterium clone D15_7_SW_G_30 16S ribosomal RNA gene, partialsequence.
HM133477	STB14	132	5	PLA491	Biosope_T84_16S_PLA491	2	Stramenopiles	Pelagophyceae			HM133477	FJ649273	94.49%	Uncultured phototrophic eukaryote clone STB1_25m_D2 16S ribosomal RNA gene,partial sequence; chloroplast.
HM133478	STB14	132	5	PLA491	Biosope_T84_16S_PLA491	3	stramenopiles	Chrysophyceae			HM133478	EF052159	98.67%	Uncultured chrysophyte clone MC615-92 16S ribosomal RNA gene, partialsequence; plastid.
HM133479	STB14	132	5	PLA491	Biosope_T84_16S_PLA491	4	Haptophyta	Prymnesiophyceae			HM133266	EF051942	99.87%	Uncultured haptophyte clone MAMA64-36 16S ribosomal RNA gene, partialsequence; plastid.
HM133480	STB14	132	5	PLA491	Biosope_T84_16S_PLA491	5	Haptophyta	Prymnesiophyceae			HM133480	AB199884	92.81%	Dinophysis mitra chloroplast gene for 16S rRNA, partial sequence,clone:DM2.
HM133481	STB14	132	5	PLA491	Biosope_T84_16S_PLA491	6	stramenopiles	Chrysophyceae			HM133470	EF574962	98.93%	Uncultured bacterium clone S25_1306 16S ribosomal RNA gene, partialsequence.
HM133482	STB14	132	5	PLA491	Biosope_T84_16S_PLA491	7	Stramenopiles	Dictyochophyceae			HM133342	FJ649290	99.87%	Uncultured phototrophic eukaryote clone STB1_25m_F8 16S ribosomal RNA gene,partial sequence; chloroplast.
HM133483	STB14	132	5	PLA491	Biosope_T84_16S_PLA491	8	stramenopiles	Chrysophyceae			HM133470	EF574962	98.67%	Uncultured bacterium clone S25_1306 16S ribosomal RNA gene, partialsequence.
HM133484	STB14	132	5	PLA491	Biosope_T84_16S_PLA491	9	stramenopiles	Bolidophyceae		Bolidomonas	HM133355	AY702144	98.53%	Bolidomonas mediterranea strain RCC238 16S ribosomal RNA gene, partialsequence; plastid.
HM133485	STB14	132	5	PLA491	Biosope_T84_16S_PLA491	10	stramenopiles	Chrysophyceae			HM133478	EF052159	99.07%	Uncultured chrysophyte clone MC615-92 16S ribosomal RNA gene, partialsequence; plastid.
HM133486	STB14	132	5	PLA491	Biosope_T84_16S_PLA491	11	Haptophyta	Prymnesiophyceae			HM133486	EF052047	99.48%	Uncultured haptophyte clone 250304-50 16S ribosomal RNA gene, partialsequence; plastid.
HM133487	STB14	132	5	PLA491	Biosope_T84_16S_PLA491	12	stramenopiles	Chrysophyceae			HM133470	EU182057	96.95%	Uncultured bacterium clone D15_7_SW_G_30 16S ribosomal RNA gene, partialsequence.
HM133488	STB14	132	5	PLA491	Biosope_T84_16S_PLA491	14	stramenopiles	Chrysophyceae			HM133478	EF052159	98.80%	Uncultured chrysophyte clone MC615-92 16S ribosomal RNA gene, partialsequence; plastid.
HM133489	STB14	132	5	PLA491	Biosope_T84_16S_PLA491	16	Stramenopiles	Dictyochophyceae			HM133342	FJ649290	98.28%	Uncultured phototrophic eukaryote clone STB1_25m_F8 16S ribosomal RNA gene,partial sequence; chloroplast.
HM133490	STB14	132	5	PLA491	Biosope_T84_16S_PLA491	17	Stramenopiles	Pelagophyceae			HM133477	FJ649273	94.79%	Uncultured phototrophic eukaryote clone STB1_25m_D2 16S ribosomal RNA gene,partial sequence; chloroplast.
HM133491	STB14	132	5	PLA491	Biosope_T84_16S_PLA491	18	stramenopiles	Chrysophyceae			HM133253	EU182057	98.41%	Uncultured bacterium clone D15_7_SW_G_30 16S ribosomal RNA gene, partialsequence.
HM133492	STB14	132	5	PLA491	Biosope_T84_16S_PLA491	19	stramenopiles	Chrysophyceae			HM133470	EF574962	98.54%	Uncultured bacterium clone S25_1306 16S ribosomal RNA gene, partialsequence.
HM133493	STB14	132	5	PLA491	Biosope_T84_16S_PLA491	20	Haptophyta	Prymnesiophyceae			HM133266	EF052162	98.83%	Uncultured haptophyte clone MC615-95 16S ribosomal RNA gene, partialsequence; plastid.
HM133494	STB14	132	5	PLA491	Biosope_T84_16S_PLA491	21	Haptophyta	Prymnesiophyceae			HM133494	FJ649305	98.16%	Uncultured phototrophic eukaryote clone STB17_30m_H1 16S ribosomal RNAgene, partial sequence; chloroplast.
HM133495	STB14	132	5	PLA491	Biosope_T84_16S_PLA491	22	Haptophyta	Prymnesiophyceae			HM133549	EF051984	99.21%	Uncultured haptophyte clone MAMA64-122 16S ribosomal RNA gene, partialsequence; plastid.
HM133496	STB14	132	5	PLA491	Biosope_T84_16S_PLA491	23	stramenopiles	Chrysophyceae			HM133470	EF574962	98.55%	Uncultured bacterium clone S25_1306 16S ribosomal RNA gene, partialsequence.
HM133497	STB14	132	5	PLA491	Biosope_T84_16S_PLA491	24	stramenopiles	Chrysophyceae			HM133470	EF574962	98.41%	Uncultured bacterium clone S25_1306 16S ribosomal RNA gene, partialsequence.
HM133498	STB14	132	5	PLA491	Biosope_T84_16S_PLA491	25	Chlorophyta	Prasinophyceae	clade 16S-IX		HM133267	FJ649270	99.61%	Uncultured phototrophic eukaryote clone STB1_25m_C10 16S ribosomal RNAgene, partial sequence; chloroplast.
HM133499	STB14	132	5	PLA491	Biosope_T84_16S_PLA491	26	Haptophyta	Prymnesiophyceae			HM133266	EF574441	99.87%	Uncultured bacterium clone S25_785 16S ribosomal RNA gene, partialsequence.
HM133500	STB14	132	5	PLA491	Biosope_T84_16S_PLA491	27	stramenopiles	Chrysophyceae			HM133470	EF574962	98.81%	Uncultured bacterium clone S25_1306 16S ribosomal RNA gene, partialsequence.
HM133501	STB14	132	5	PLA491	Biosope_T84_16S_PLA491	28	Haptophyta	Prymnesiophyceae			HM133339	DQ395642	99.87%	Uncultured organism clone ctg_CGOF234 16S ribosomal RNA gene, partialsequence.
HM133502	STB14	132	5	PLA491	Biosope_T84_16S_PLA491	29	Chlorophyta	Prasinophyceae	clade 16S-IX		HM133267	FJ649270	99.74%	Uncultured phototrophic eukaryote clone STB1_25m_C10 16S ribosomal RNAgene, partial sequence; chloroplast.
HM133503	STB14	132	5	PLA491	Biosope_T84_16S_PLA491	30	Haptophyta	Prymnesiophyceae			HM133503	EF052003	98.83%	Uncultured haptophyte clone 250304-3 16S ribosomal RNA gene, partialsequence; plastid.
HM133504	STB14	132	5	PLA491	Biosope_T84_16S_PLA491	31	Haptophyta	Prymnesiophyceae			HM133546	EF574745	99.61%	Uncultured bacterium clone S25_1089 16S ribosomal RNA gene, partialsequence.
HM133505	STB14	133	150	OXY107	Biosope_T88_16S_OXY107	1	Stramenopiles	Pelagophyceae			HM133308	DQ513100	99.61%	Uncultured bacterium clone FS274-44B-03 16S ribosomal RNA gene, partialsequence.
HM133506	STB14	133	150	OXY107	Biosope_T88_16S_OXY107	2	Stramenopiles	Pelagophyceae			HM133308	FJ649273	99.61%	Uncultured phototrophic eukaryote clone STB1_25m_D2 16S ribosomal RNA gene,partial sequence; chloroplast.
HM133507	STB14	133	150	OXY107	Biosope_T88_16S_OXY107	3	Stramenopiles	Dictyochophyceae			HM133342	EF052136	99.34%	Uncultured dictyochophyte clone MC615-64 16S ribosomal RNA gene, partialsequence; plastid.
HM133508	STB14	133	150	OXY107	Biosope_T88_16S_OXY107	5	Stramenopiles	Pelagophyceae			HM133308	FJ649273	100.00%	Uncultured phototrophic eukaryote clone STB1_25m_D2 16S ribosomal RNA gene,partial sequence; chloroplast.
HM133509	STB14	133	150	OXY107	Biosope_T88_16S_OXY107	6	Stramenopiles	Pelagophyceae			HM133308	FJ649273	100.00%	Uncultured phototrophic eukaryote clone STB1_25m_D2 16S ribosomal RNA gene,partial sequence; chloroplast.
HM133510	STB14	133	150	OXY107	Biosope_T88_16S_OXY107	8	Stramenopiles	Pelagophyceae			HM133308	FJ649273	98.94%	Uncultured phototrophic eukaryote clone STB1_25m_D2 16S ribosomal RNA gene,partial sequence; chloroplast.
HM133511	STB14	133	150	OXY107	Biosope_T88_16S_OXY107	9	Stramenopiles	Pelagophyceae			HM133308	FJ649273	100.00%	Uncultured phototrophic eukaryote clone STB1_25m_D2 16S ribosomal RNA gene,partial sequence; chloroplast.
HM133512	STB14	133	150	OXY107	Biosope_T88_16S_OXY107	10	Stramenopiles	Pelagophyceae			HM133308	FJ649273	99.74%	Uncultured phototrophic eukaryote clone STB1_25m_D2 16S ribosomal RNA gene,partial sequence; chloroplast.
HM133513	STB14	133	150	OXY107	Biosope_T88_16S_OXY107	11	stramenopiles	Dictyochophyceae			HM133342	EF052136	99.34%	Uncultured dictyochophyte clone MC615-64 16S ribosomal RNA gene, partialsequence; plastid.
HM133514	STB14	133	150	OXY107	Biosope_T88_16S_OXY107	12	Stramenopiles	Pelagophyceae			HM133308	DQ513100	100.00%	Uncultured bacterium clone FS274-44B-03 16S ribosomal RNA gene, partialsequence.
HM133515	STB14	133	150	OXY107	Biosope_T88_16S_OXY107	13	Stramenopiles	Pelagophyceae			HM133308	FJ649273	99.48%	Uncultured phototrophic eukaryote clone STB1_25m_D2 16S ribosomal RNA gene,partial sequence; chloroplast.
HM133516	STB14	133	150	OXY107	Biosope_T88_16S_OXY107	14	Stramenopiles	Pelagophyceae			HM133308	DQ513100	100.00%	Uncultured bacterium clone FS274-44B-03 16S ribosomal RNA gene, partialsequence.
HM133517	STB14	133	150	OXY107	Biosope_T88_16S_OXY107	15	Stramenopiles	Pelagophyceae			HM133308	FJ649273	99.47%	Uncultured phototrophic eukaryote clone STB1_25m_D2 16S ribosomal RNA gene,partial sequence; chloroplast.
HM133518	STB14	133	150	OXY107	Biosope_T88_16S_OXY107	16	Stramenopiles	Pelagophyceae			HM133308	DQ513100	99.74%	Uncultured bacterium clone FS274-44B-03 16S ribosomal RNA gene, partialsequence.
HM133519	STB14	133	150	OXY107	Biosope_T88_16S_OXY107	19	Stramenopiles	Pelagophyceae			HM133308	FJ649273	99.74%	Uncultured phototrophic eukaryote clone STB1_25m_D2 16S ribosomal RNA gene,partial sequence; chloroplast.
HM133520	STB14	133	150	OXY107	Biosope_T88_16S_OXY107	20	Stramenopiles	Pelagophyceae			HM133308	FJ649273	99.34%	Uncultured phototrophic eukaryote clone STB1_25m_D2 16S ribosomal RNA gene,partial sequence; chloroplast.
HM133521	STB14	133	150	OXY107	Biosope_T88_16S_OXY107	21	Stramenopiles	Pelagophyceae			HM133308	FJ649273	100.00%	Uncultured phototrophic eukaryote clone STB1_25m_D2 16S ribosomal RNA gene,partial sequence; chloroplast.
HM133522	STB14	133	150	OXY107	Biosope_T88_16S_OXY107	22	Stramenopiles	Dictyochophyceae			HM133522	FJ826020	99.48%	Uncultured phototrophic eukaryote clone BM1-8-32 16S ribosomal RNA gene,partial sequence; plastid.
HM133523	STB14	133	150	OXY107	Biosope_T88_16S_OXY107	23	Stramenopiles	Pelagophyceae			HM133308	DQ513100	99.87%	Uncultured bacterium clone FS274-44B-03 16S ribosomal RNA gene, partialsequence.
HM133524	STB14	133	150	OXY107	Biosope_T88_16S_OXY107	24	Stramenopiles	Pelagophyceae			HM133308	FJ649273	99.08%	Uncultured phototrophic eukaryote clone STB1_25m_D2 16S ribosomal RNA gene,partial sequence; chloroplast.
HM133525	STB14	133	150	OXY107	Biosope_T88_16S_OXY107	25	Stramenopiles	Pelagophyceae			HM133308	FJ649273	99.74%	Uncultured phototrophic eukaryote clone STB1_25m_D2 16S ribosomal RNA gene,partial sequence; chloroplast.
HM133526	STB14	133	150	OXY107	Biosope_T88_16S_OXY107	26	Stramenopiles	Pelagophyceae			HM133308	FJ649273	99.74%	Uncultured phototrophic eukaryote clone STB1_25m_D2 16S ribosomal RNA gene,partial sequence; chloroplast.
HM133527	STB14	133	150	OXY107	Biosope_T88_16S_OXY107	27	Stramenopiles	Pelagophyceae			HM133308	FJ649273	99.47%	Uncultured phototrophic eukaryote clone STB1_25m_D2 16S ribosomal RNA gene,partial sequence; chloroplast.
HM133528	STB14	133	150	OXY107	Biosope_T88_16S_OXY107	28	Stramenopiles	Pelagophyceae			HM133308	FJ649273	99.87%	Uncultured phototrophic eukaryote clone STB1_25m_D2 16S ribosomal RNA gene,partial sequence; chloroplast.
HM133529	STB14	133	150	OXY107	Biosope_T88_16S_OXY107	29	Stramenopiles	Pelagophyceae			HM133308	FJ649273	99.48%	Uncultured phototrophic eukaryote clone STB1_25m_D2 16S ribosomal RNA gene,partial sequence; chloroplast.
HM133530	STB14	133	150	OXY107	Biosope_T88_16S_OXY107	31	Stramenopiles	Pelagophyceae			HM133308	FJ649273	99.47%	Uncultured phototrophic eukaryote clone STB1_25m_D2 16S ribosomal RNA gene,partial sequence; chloroplast.
HM133531	STB14	133	150	OXY107	Biosope_T88_16S_OXY107	32	Stramenopiles	Pelagophyceae			HM133308	DQ513100	99.35%	Uncultured bacterium clone FS274-44B-03 16S ribosomal RNA gene, partialsequence.
HM133532	STB14	133	150	OXY107	Biosope_T88_16S_OXY107	34	Stramenopiles	Pelagophyceae			HM133308	FJ649273	99.60%	Uncultured phototrophic eukaryote clone STB1_25m_D2 16S ribosomal RNA gene,partial sequence; chloroplast.
HM133533	STB14	133	150	OXY107	Biosope_T88_16S_OXY107	35	Stramenopiles	Pelagophyceae			HM133308	FJ649273	99.87%	Uncultured phototrophic eukaryote clone STB1_25m_D2 16S ribosomal RNA gene,partial sequence; chloroplast.
HM133534	STB14	133	150	OXY107	Biosope_T88_16S_OXY107	36	Stramenopiles	Pelagophyceae			HM133308	FJ649273	99.87%	Uncultured phototrophic eukaryote clone STB1_25m_D2 16S ribosomal RNA gene,partial sequence; chloroplast.

Accession	station	CTD cast	depth	primer_forward	Clone_library	Clone	Division	Class	Order	Genus	OTU	BLAST Accession	BLAST % ID	BLAST Description
HM133535	STB14	133	150	OXY107	Biosope_T88_16S_OXY107	40	Stramenopiles	Dictyochophyceae			HM133342	FJ649290	98.55%	Uncultured phototrophic eukaryote clone STB1_25m_F8 16S ribosomal RNA gene,partial sequence; chloroplast.
HM133536	STB14	133	150	OXY107	Biosope_T88_16S_OXY107	43	Stramenopiles	Pelagophyceae			HM133308	FJ649273	100.00%	Uncultured phototrophic eukaryote clone STB1_25m_D2 16S ribosomal RNA gene,partial sequence; chloroplast.
HM133537	STB14	133	150	OXY107	Biosope_T88_16S_OXY107	44	Stramenopiles	Pelagophyceae			HM133308	FJ649273	99.87%	Uncultured phototrophic eukaryote clone STB1_25m_D2 16S ribosomal RNA gene,partial sequence; chloroplast.
HM133538	STB14	133	150	OXY107	Biosope_T88_16S_OXY107	47	Stramenopiles	Pelagophyceae			HM133308	FJ649273	99.87%	Uncultured phototrophic eukaryote clone STB1_25m_D2 16S ribosomal RNA gene,partial sequence; chloroplast.
HM133539	STB14	133	150	OXY107	Biosope_T88_16S_OXY107	48	Stramenopiles	Pelagophyceae			HM133308	FJ649273	99.34%	Uncultured phototrophic eukaryote clone STB1_25m_D2 16S ribosomal RNA gene,partial sequence; chloroplast.
HM133540	STB14	133	150	PLA491	Biosope_T88_16S_PLA491	9	Stramenopiles	Pelagophyceae			HM133308	FJ649273	99.87%	Uncultured phototrophic eukaryote clone STB1_25m_D2 16S ribosomal RNA gene,partial sequence; chloroplast.
HM133541	STB14	133	150	PLA491	Biosope_T88_16S_PLA491	10	Stramenopiles	Pelagophyceae			HM133308	FJ649273	100.00%	Uncultured phototrophic eukaryote clone STB1_25m_D2 16S ribosomal RNA gene,partial sequence; chloroplast.
HM133542	STB14	133	150	PLA491	Biosope_T88_16S_PLA491	13	Haptophyta	Prymnesiophyceae			HM133546	EF574745	99.61%	Uncultured bacterium clone S25_1089 16S ribosomal RNA gene, partialsequence.
HM133543	STB14	133	150	PLA491	Biosope_T88_16S_PLA491	14	Haptophyta	Prymnesiophyceae			HM133546	EF574745	99.74%	Uncultured bacterium clone S25_1089 16S ribosomal RNA gene, partialsequence.
HM133544	STB14	133	150	PLA491	Biosope_T88_16S_PLA491	15	Chlorophyta	Prasinophyceae	clade 16S-VIII		HM133550	FJ649309	99.87%	Uncultured phototrophic eukaryote clone STB17_30m_H5 16S ribosomal RNAgene, partial sequence; chloroplast.
HM133545	STB14	133	150	PLA491	Biosope_T88_16S_PLA491	16	Chlorophyta	Prasinophyceae	clade 16S-VIII		HM133550	FJ649309	99.47%	Uncultured phototrophic eukaryote clone STB17_30m_H5 16S ribosomal RNAgene, partial sequence; chloroplast.
HM133546	STB14	133	150	PLA491	Biosope_T88_16S_PLA491	17	Haptophyta	Prymnesiophyceae			HM133546	EF574745	99.74%	Uncultured bacterium clone S25_1089 16S ribosomal RNA gene, partialsequence.
HM133547	STB14	133	150	PLA491	Biosope_T88_16S_PLA491	18	Stramenopiles	Pelagophyceae			HM133308	FJ649273	100.00%	Uncultured phototrophic eukaryote clone STB1_25m_D2 16S ribosomal RNA gene,partial sequence; chloroplast.
HM133548	STB14	133	150	PLA491	Biosope_T88_16S_PLA491	19	Chlorophyta	Prasinophyceae	clade 16S-VIII		HM133550	FJ649309	99.74%	Uncultured phototrophic eukaryote clone STB17_30m_H5 16S ribosomal RNAgene, partial sequence; chloroplast.
HM133549	STB14	133	150	PLA491	Biosope_T88_16S_PLA491	20	Haptophyta	Prymnesiophyceae			HM133549	EF051984	99.35%	Uncultured haptophyte clone MAMA64-122 16S ribosomal RNA gene, partialsequence; plastid.
HM133550	STB14	133	150	PLA491	Biosope_T88_16S_PLA491	21	Chlorophyta	Prasinophyceae	clade 16S-VIII		HM133550	FJ649309	99.74%	Uncultured phototrophic eukaryote clone STB17_30m_H5 16S ribosomal RNAgene, partial sequence; chloroplast.
HM133551	STB14	133	150	PLA491	Biosope_T88_16S_PLA491	22	Stramenopiles	Pelagophyceae			HM133308	FJ649273	99.87%	Uncultured phototrophic eukaryote clone STB1_25m_D2 16S ribosomal RNA gene,partial sequence; chloroplast.
HM133552	STB14	133	150	PLA491	Biosope_T88_16S_PLA491	23	Stramenopiles	Pelagophyceae			HM133308	FJ649273	99.47%	Uncultured phototrophic eukaryote clone STB1_25m_D2 16S ribosomal RNA gene,partial sequence; chloroplast.
HM133553	STB14	133	150	PLA491	Biosope_T88_16S_PLA491	24	Haptophyta	Prymnesiophyceae			HM133549	EF051984	99.35%	Uncultured haptophyte clone MAMA64-122 16S ribosomal RNA gene, partialsequence; plastid.
HM133554	STB14	133	150	PLA491	Biosope_T88_16S_PLA491	25	Stramenopiles	Pelagophyceae			HM133308	FJ649273	99.60%	Uncultured phototrophic eukaryote clone STB1_25m_D2 16S ribosomal RNA gene,partial sequence; chloroplast.
HM133555	STB14	133	150	PLA491	Biosope_T88_16S_PLA491	26	Haptophyta	Prymnesiophyceae			HM133546	EF574745	99.61%	Uncultured bacterium clone S25_1089 16S ribosomal RNA gene, partialsequence.
HM133556	STB14	133	150	PLA491	Biosope_T88_16S_PLA491	27	Chlorophyta	Prasinophyceae	clade 16S-VIII		HM133550	FJ649309	99.60%	Uncultured phototrophic eukaryote clone STB17_30m_H5 16S ribosomal RNAgene, partial sequence; chloroplast.
HM133557	STB14	133	150	PLA491	Biosope_T88_16S_PLA491	28	Chlorophyta	Prasinophyceae	clade 16S-VIII		HM133550	FJ649309	99.87%	Uncultured phototrophic eukaryote clone STB17_30m_H5 16S ribosomal RNAgene, partial sequence; chloroplast.
HM133558	STB14	133	150	PLA491	Biosope_T88_16S_PLA491	29	Haptophyta	Prymnesiophyceae			HM133065	EF052003	99.35%	Uncultured haptophyte clone 250304-3 16S ribosomal RNA gene, partialsequence; plastid.
HM133559	STB14	133	150	PLA491	Biosope_T88_16S_PLA491	30	Haptophyta	Prymnesiophyceae			HM133266	EF574945	98.42%	Uncultured bacterium clone S25_1289 16S ribosomal RNA gene, partialsequence.
HM133560	STB14	133	150	PLA491	Biosope_T88_16S_PLA491	31	Haptophyta	Prymnesiophyceae			HM133266	EF574945	98.55%	Uncultured bacterium clone S25_1289 16S ribosomal RNA gene, partialsequence.
HM133561	STB14	133	150	PLA491	Biosope_T88_16S_PLA491	32	Haptophyta	Prymnesiophyceae			HM133339	DQ395642	99.74%	Uncultured organism clone ctg_CGOF234 16S ribosomal RNA gene, partialsequence.
HM133562	STB14	133	150	PLA491	Biosope_T88_16S_PLA491	33	Chlorophyta	Prasinophyceae	clade 16S-VIII		HM133550	FJ649309	99.47%	Uncultured phototrophic eukaryote clone STB17_30m_H5 16S ribosomal RNAgene, partial sequence; chloroplast.
HM133563	STB14	133	150	PLA491	Biosope_T88_16S_PLA491	34	Haptophyta	Prymnesiophyceae			HM133339	DQ395642	99.87%	Uncultured organism clone ctg_CGOF234 16S ribosomal RNA gene, partialsequence.
HM133564	STB14	133	150	PLA491	Biosope_T88_16S_PLA491	35	Haptophyta	Prymnesiophyceae			HM133339	DQ395642	100.00%	Uncultured organism clone ctg_CGOF234 16S ribosomal RNA gene, partialsequence.
HM133565	STB14	133	150	PLA491	Biosope_T88_16S_PLA491	36	Haptophyta	Prymnesiophyceae			HM133546	EF574745	99.74%	Uncultured bacterium clone S25_1089 16S ribosomal RNA gene, partialsequence.
HM133566	STB14	133	150	PLA491	Biosope_T88_16S_PLA491	37	Stramenopiles	Pelagophyceae			HM133308	FJ649273	99.87%	Uncultured phototrophic eukaryote clone STB1_25m_D2 16S ribosomal RNA gene,partial sequence; chloroplast.
HM133567	STB14	133	150	PLA491	Biosope_T88_16S_PLA491	38	Stramenopiles	Pelagophyceae			HM133308	FJ649273	99.87%	Uncultured phototrophic eukaryote clone STB1_25m_D2 16S ribosomal RNA gene,partial sequence; chloroplast.
HM133568	STB14	133	150	PLA491	Biosope_T88_16S_PLA491	39	Haptophyta	Prymnesiophyceae			HM133549	EF051984	99.21%	Uncultured haptophyte clone MAMA64-122 16S ribosomal RNA gene, partialsequence; plastid.
HM133569	STB14	133	150	PLA491	Biosope_T88_16S_PLA491	40	Haptophyta	Prymnesiophyceae			HM133503	EF574945	97.13%	Uncultured bacterium clone S25_1289 16S ribosomal RNA gene, partialsequence.
HM132988	STB17	178	20	OXY107	Biosope_T123_16S_OXY107	2	Stramenopiles	Pelagophyceae			HM133308	FJ649273	99.85%	Uncultured phototrophic eukaryote clone STB1_25m_D2 16S ribosomal RNA gene,partial sequence; chloroplast.
HM132989	STB17	178	20	OXY107	Biosope_T123_16S_OXY107	3	Stramenopiles	Pelagophyceae			HM133308	FJ649273	99.70%	Uncultured phototrophic eukaryote clone STB1_25m_D2 16S ribosomal RNA gene,partial sequence; chloroplast.
HM132990	STB17	178	20	OXY107	Biosope_T123_16S_OXY107	4	Stramenopiles	Pelagophyceae			HM133308	FJ649273	98.83%	Uncultured phototrophic eukaryote clone STB1_25m_D2 16S ribosomal RNA gene,partial sequence; chloroplast.
HM132991	STB17	178	20	OXY107	Biosope_T123_16S_OXY107	5	Stramenopiles	Pelagophyceae			HM133308	FJ649273	100.00%	Uncultured phototrophic eukaryote clone STB1_25m_D2 16S ribosomal RNA gene,partial sequence; chloroplast.
HM132992	STB17	178	20	OXY107	Biosope_T123_16S_OXY107	6	Stramenopiles	Pelagophyceae			HM133308	FJ649273	99.71%	Uncultured phototrophic eukaryote clone STB1_25m_D2 16S ribosomal RNA gene,partial sequence; chloroplast.
HM132993	STB17	178	20	OXY107	Biosope_T123_16S_OXY107	7	Stramenopiles	Pelagophyceae			HM133308	DQ513100	99.43%	Uncultured bacterium clone FS274-44B-03 16S ribosomal RNA gene, partialsequence.
HM132994	STB17	178	20	OXY107	Biosope_T123_16S_OXY107	8	Haptophyta	Prymnesiophyceae			HM133136	FJ649298	99.44%	Uncultured phototrophic eukaryote clone STB17_30m_G6 16S ribosomal RNAgene, partial sequence; chloroplast.
HM132995	STB17	178	20	OXY107	Biosope_T123_16S_OXY107	9	Stramenopiles	Pelagophyceae			HM133308	FJ649273	99.72%	Uncultured phototrophic eukaryote clone STB1_25m_D2 16S ribosomal RNA gene,partial sequence; chloroplast.
HM132996	STB17	178	20	OXY107	Biosope_T123_16S_OXY107	10	stramenopiles	Chrysophyceae			HM133470	EF574962	99.06%	Uncultured bacterium clone S25_1306 16S ribosomal RNA gene, partialsequence.
HM132997	STB17	178	20	OXY107	Biosope_T123_16S_OXY107	11	Stramenopiles	Pelagophyceae			HM133308	FJ649273	100.00%	Uncultured phototrophic eukaryote clone STB1_25m_D2 16S ribosomal RNA gene,partial sequence; chloroplast.
HM132998	STB17	178	20	OXY107	Biosope_T123_16S_OXY107	12	Stramenopiles	Pelagophyceae			HM133308	DQ513100	100.00%	Uncultured bacterium clone FS274-44B-03 16S ribosomal RNA gene, partialsequence.
HM132999	STB17	178	20	OXY107	Biosope_T123_16S_OXY107	13	Stramenopiles	Pelagophyceae			HM133308	FJ649273	99.85%	Uncultured phototrophic eukaryote clone STB1_25m_D2 16S ribosomal RNA gene,partial sequence; chloroplast.
HM133000	STB17	178	20	OXY107	Biosope_T123_16S_OXY107	14	Stramenopiles	Pelagophyceae			HM133308	DQ513100	99.19%	Uncultured bacterium clone FS274-44B-03 16S ribosomal RNA gene, partialsequence.
HM133001	STB17	178	20	OXY107	Biosope_T123_16S_OXY107	15	Stramenopiles	Pelagophyceae			HM133308	DQ513100	100.00%	Uncultured bacterium clone FS274-44B-03 16S ribosomal RNA gene, partialsequence.
HM133002	STB17	178	20	OXY107	Biosope_T123_16S_OXY107	16	Stramenopiles	Pelagophyceae			HM133308	DQ513100	100.00%	Uncultured bacterium clone FS274-44B-03 16S ribosomal RNA gene, partialsequence.
HM133003	STB17	178	20	OXY107	Biosope_T123_16S_OXY107	17	Stramenopiles	Pelagophyceae			HM133308	DQ513100	99.85%	Uncultured bacterium clone FS274-44B-03 16S ribosomal RNA gene, partialsequence.
HM133004	STB17	178	20	OXY107	Biosope_T123_16S_OXY107	18	stramenopiles	Chrysophyceae			HM133253	EF052086	98.44%	Uncultured chrysophyte clone MC615-9 16S ribosomal RNA gene, partialsequence; plastid.
HM133005	STB17	178	20	OXY107	Biosope_T123_16S_OXY107	19	stramenopiles	Chrysophyceae			HM133470	FJ649291	98.70%	Uncultured phototrophic eukaryote clone STB1_25m_F10 16S ribosomal RNAgene, partial sequence; chloroplast.
HM133006	STB17	178	20	OXY107	Biosope_T123_16S_OXY107	20	Stramenopiles	Pelagophyceae			HM133308	FJ649273	99.49%	Uncultured phototrophic eukaryote clone STB1_25m_D2 16S ribosomal RNA gene,partial sequence; chloroplast.
HM133007	STB17	178	20	OXY107	Biosope_T123_16S_OXY107	21	Stramenopiles	Pelagophyceae			HM133308	DQ513100	99.51%	Uncultured bacterium clone FS274-44B-03 16S ribosomal RNA gene, partialsequence.
HM133008	STB17	178	20	OXY107	Biosope_T123_16S_OXY107	22	Stramenopiles	Pelagophyceae			HM133308	DQ513100	99.53%	Uncultured bacterium clone FS274-44B-03 16S ribosomal RNA gene, partialsequence.
HM133009	STB17	178	20	OXY107	Biosope_T123_16S_OXY107	23	Stramenopiles	Pelagophyceae			HM133308	FJ649273	99.85%	Uncultured phototrophic eukaryote clone STB1_25m_D2 16S ribosomal RNA gene,partial sequence; chloroplast.
HM133010	STB17	178	20	OXY107	Biosope_T123_16S_OXY107	24	Stramenopiles	Pelagophyceae			HM133308	FJ649273	99.72%	Uncultured phototrophic eukaryote clone STB1_25m_D2 16S ribosomal RNA gene,partial sequence; chloroplast.
HM133011	STB17	178	20	PLA491	Biosope_T123_16S_PLA491	1	Chlorophyta	Prasinophyceae	clade 16S-VIII		HM133550	EU249955	99.73%	Uncultured phototrophic eukaryote clone PmeaH2OG10 16S ribosomal RNA gene,partial sequence; chloroplast.
HM133012	STB17	178	20	PLA491	Biosope_T123_16S_PLA491	2	Chlorophyta	Prasinophyceae	clade 16S-VIII		HM133550	EU249955	99.87%	Uncultured phototrophic eukaryote clone PmeaH2OG10 16S ribosomal RNA gene,partial sequence; chloroplast.
HM133013	STB17	178	20	PLA491	Biosope_T123_16S_PLA491	3	Stramenopiles	Pelagophyceae			HM133308	FJ649273	97.05%	Uncultured phototrophic eukaryote clone STB1_25m_D2 16S ribosomal RNA gene,partial sequence; chloroplast.
HM133014	STB17	178	20	PLA491	Biosope_T123_16S_PLA491	4	Chlorophyta	Prasinophyceae	clade 16S-VIII		HM133550	EU249955	99.73%	Uncultured phototrophic eukaryote clone PmeaH2OG10 16S ribosomal RNA gene,partial sequence; chloroplast.
HM133015	STB17	178	20	PLA491	Biosope_T123_16S_PLA491	5	Haptophyta	Prymnesiophyceae			HM133503	EF052064	99.87%	Uncultured haptophyte clone 250304-77 16S ribosomal RNA gene, partialsequence; plastid.
HM133016	STB17	178	20	PLA491	Biosope_T123_16S_PLA491	6	Haptophyta	Prymnesiophyceae			HM133546	EF574745	99.86%	Uncultured bacterium clone S25_1089 16S ribosomal RNA gene, partialsequence.
HM133017	STB17	178	20	PLA491	Biosope_T123_16S_PLA491	7	Haptophyta	Prymnesiophyceae			HM133266	EF574441	99.72%	Uncultured bacterium clone S25_785 16S ribosomal RNA gene, partialsequence.
HM133018	STB17	178	20	PLA491	Biosope_T123_16S_PLA491	8	stramenopiles	Chrysophyceae			HM133470	EF574962	98.68%	Uncultured bacterium clone S25_1306 16S ribosomal RNA gene, partialsequence.
HM133019	STB17	178	20	PLA491	Biosope_T123_16S_PLA491	9	Haptophyta	Prymnesiophyceae			HM133546	EF574745	99.86%	Uncultured bacterium clone S25_1089 16S ribosomal RNA gene, partialsequence.

Accession	station	CTD cast	depth	primer_forward	Clone_library	Clone	Division	Class	Order	Genus	OTU	BLAST Accession	BLAST % ID	BLAST Description
HM133020	STB17	178	20	PLA491	Biosope_T123_16S_PLA491	10	stramenopiles	Bolidophyceae			HM133355	AY702173	99.56%	Uncultured phototrophic eukaryote clone DYF38 16S ribosomal RNA gene,partial sequence; plastid.
HM133021	STB17	178	20	PLA491	Biosope_T123_16S_PLA491	11	Chlorophyta	Prasinophyceae	clade 16S-VIII		HM133550	EU249955	99.60%	Uncultured phototrophic eukaryote clone PmeaH2OG10 16S ribosomal RNA gene,partial sequence; chloroplast.
HM133022	STB17	178	20	PLA491	Biosope_T123_16S_PLA491	12	Chlorophyta	Prasinophyceae	clade 16S-VIII		HM133550	EU249955	99.86%	Uncultured phototrophic eukaryote clone PmeaH2OG10 16S ribosomal RNA gene,partial sequence; chloroplast.
HM133023	STB17	178	20	PLA491	Biosope_T123_16S_PLA491	13	Stramenopiles	Pelagophyceae			HM133308	DQ513100	99.87%	Uncultured bacterium clone FS274-44B-03 16S ribosomal RNA gene, partialsequence.
HM133024	STB17	178	20	PLA491	Biosope_T123_16S_PLA491	14	Haptophyta	Prymnesiophyceae			HM133503	EF052003	98.81%	Uncultured haptophyte clone 250304-3 16S ribosomal RNA gene, partialsequence; plastid.
HM133025	STB17	178	20	PLA491	Biosope_T123_16S_PLA491	15	Haptophyta	Prymnesiophyceae			HM133339	DQ395642	99.87%	Uncultured organism clone ctg_CGOF234 16S ribosomal RNA gene, partialsequence.
HM133026	STB17	178	20	PLA491	Biosope_T123_16S_PLA491	16	Stramenopiles	Dictyochophyceae			HM133300	FJ826020	99.30%	Uncultured phototrophic eukaryote clone BM1-8-32 16S ribosomal RNA gene,partial sequence; plastid.
HM133027	STB17	178	20	PLA491	Biosope_T123_16S_PLA491	17	Chlorophyta	Prasinophyceae	clade 16S-VIII		HM133550	EU249955	99.73%	Uncultured phototrophic eukaryote clone PmeaH2OG10 16S ribosomal RNA gene,partial sequence; chloroplast.
HM133028	STB17	178	20	PLA491	Biosope_T123_16S_PLA491	18	Haptophyta	Prymnesiophyceae			HM133339	DQ395642	99.86%	Uncultured organism clone ctg_CGOF234 16S ribosomal RNA gene, partialsequence.
HM133029	STB17	178	20	PLA491	Biosope_T123_16S_PLA491	19	Chlorophyta	Prasinophyceae	clade 16S-VIII		HM133550	FJ649309	99.73%	Uncultured phototrophic eukaryote clone STB17_30m_H5 16S ribosomal RNAgene, partial sequence; chloroplast.
HM133030	STB17	178	20	PLA491	Biosope_T123_16S_PLA491	20	Stramenopiles	Pelagophyceae			HM133308	FJ649273	99.47%	Uncultured phototrophic eukaryote clone STB1_25m_D2 16S ribosomal RNA gene,partial sequence; chloroplast.
HM133031	STB17	178	20	PLA491	Biosope_T123_16S_PLA491	21	Haptophyta	Prymnesiophyceae			HM133546	EF574745	99.74%	Uncultured bacterium clone S25_1089 16S ribosomal RNA gene, partialsequence.
HM133032	STB17	178	20	PLA491	Biosope_T123_16S_PLA491	22	stramenopiles	Chrysophyceae			HM133470	EF574962	98.67%	Uncultured bacterium clone S25_1306 16S ribosomal RNA gene, partialsequence.
HM133033	STB17	178	20	PLA491	Biosope_T123_16S_PLA491	23	stramenopiles	Chrysophyceae			HM133478	EF052159	98.75%	Uncultured chrysophyte clone MC615-92 16S ribosomal RNA gene, partialsequence; plastid.
HM133034	STB17	178	20	PLA491	Biosope_T123_16S_PLA491	24	Chlorophyta	Prasinophyceae	clade 16S-VIII		HM133550	EU249955	99.72%	Uncultured phototrophic eukaryote clone PmeaH2OG10 16S ribosomal RNA gene,partial sequence; chloroplast.
HM133035	STB17	178	20	PLA491	Biosope_T123_16S_PLA491	25	Chlorophyta	Prasinophyceae	clade 16S-VIII		HM133550	FJ649309	99.73%	Uncultured phototrophic eukaryote clone STB17_30m_H5 16S ribosomal RNAgene, partial sequence; chloroplast.
HM133036	STB17	178	20	PLA491	Biosope_T123_16S_PLA491	26	Stramenopiles	Bacillariophyceae			HM133036	FJ649245	99.87%	Uncultured phototrophic eukaryote clone MAR1_15m_A5 16S ribosomal RNA gene,partial sequence; chloroplast.
HM133037	STB17	178	20	PLA491	Biosope_T123_16S_PLA491	27	Chlorophyta	Prasinophyceae	clade 16S-VIII		HM133550	EU249955	100.00%	Uncultured phototrophic eukaryote clone PmeaH2OG10 16S ribosomal RNA gene,partial sequence; chloroplast.
HM133038	STB17	178	20	PLA491	Biosope_T123_16S_PLA491	28	Stramenopiles	Pelagophyceae			HM133308	FJ649273	99.85%	Uncultured phototrophic eukaryote clone STB1_25m_D2 16S ribosomal RNA gene,partial sequence; chloroplast.
HM133039	STB17	178	20	PLA491	Biosope_T123_16S_PLA491	29	Haptophyta	Prymnesiophyceae			HM133486	EF052248	98.53%	Uncultured haptophyte clone MC622-90 16S ribosomal RNA gene, partialsequence; plastid.
HM133040	STB17	178	20	PLA491	Biosope_T123_16S_PLA491	30	Chlorophyta	Prasinophyceae	clade 16S-VIII		HM133550	EU249955	100.00%	Uncultured phototrophic eukaryote clone PmeaH2OG10 16S ribosomal RNA gene,partial sequence; chloroplast.
HM133041	STB17	178	20	PLA491	Biosope_T123_16S_PLA491	31	Haptophyta	Prymnesiophyceae			HM133549	EF051984	99.47%	Uncultured haptophyte clone MAMA64-122 16S ribosomal RNA gene, partialsequence; plastid.
HM133042	STB17	178	20	PLA491	Biosope_T123_16S_PLA491	32	Haptophyta	Prymnesiophyceae			HM133065	EF052003	98.72%	Uncultured haptophyte clone 250304-3 16S ribosomal RNA gene, partialsequence; plastid.
HM132934	STB17	178	70	OXY107	Biosope_T120_16S_OXY107	1	Stramenopiles	Pelagophyceae			HM133308	FJ649273	99.74%	Uncultured phototrophic eukaryote clone STB1_25m_D2 16S ribosomal RNA gene,partial sequence; chloroplast.
HM132935	STB17	178	70	OXY107	Biosope_T120_16S_OXY107	6	Stramenopiles	Pelagophyceae			HM133308	DQ513100	100.00%	Uncultured bacterium clone FS274-44B-03 16S ribosomal RNA gene, partialsequence.
HM132936	STB17	178	70	OXY107	Biosope_T120_16S_OXY107	7	Stramenopiles	Pelagophyceae			HM133308	FJ649273	99.87%	Uncultured phototrophic eukaryote clone STB1_25m_D2 16S ribosomal RNA gene,partial sequence; chloroplast.
HM132937	STB17	178	70	OXY107	Biosope_T120_16S_OXY107	8	Stramenopiles	Pelagophyceae			HM133308	FJ649273	99.73%	Uncultured phototrophic eukaryote clone STB1_25m_D2 16S ribosomal RNA gene,partial sequence; chloroplast.
HM132938	STB17	178	70	OXY107	Biosope_T120_16S_OXY107	9	Stramenopiles	Pelagophyceae			HM133308	FJ649273	99.73%	Uncultured phototrophic eukaryote clone STB1_25m_D2 16S ribosomal RNA gene,partial sequence; chloroplast.
HM132939	STB17	178	70	OXY107	Biosope_T120_16S_OXY107	13	Stramenopiles	Pelagophyceae			HM133308	FJ649273	100.00%	Uncultured phototrophic eukaryote clone STB1_25m_D2 16S ribosomal RNA gene,partial sequence; chloroplast.
HM132940	STB17	178	70	OXY107	Biosope_T120_16S_OXY107	14	Stramenopiles	Pelagophyceae			HM133308	DQ513100	99.87%	Uncultured bacterium clone FS274-44B-03 16S ribosomal RNA gene, partialsequence.
HM132941	STB17	178	70	OXY107	Biosope_T120_16S_OXY107	15	Stramenopiles	Pelagophyceae			HM133308	FJ649273	99.87%	Uncultured phototrophic eukaryote clone STB1_25m_D2 16S ribosomal RNA gene,partial sequence; chloroplast.
HM132942	STB17	178	70	OXY107	Biosope_T120_16S_OXY107	16	Stramenopiles	Pelagophyceae			HM133308	FJ649273	99.61%	Uncultured phototrophic eukaryote clone STB1_25m_D2 16S ribosomal RNA gene,partial sequence; chloroplast.
HM132943	STB17	178	70	OXY107	Biosope_T120_16S_OXY107	17	Stramenopiles	Pelagophyceae			HM133308	FJ649273	99.74%	Uncultured phototrophic eukaryote clone STB1_25m_D2 16S ribosomal RNA gene,partial sequence; chloroplast.
HM132944	STB17	178	70	OXY107	Biosope_T120_16S_OXY107	18	Stramenopiles	Pelagophyceae			HM133308	FJ649273	99.74%	Uncultured phototrophic eukaryote clone STB1_25m_D2 16S ribosomal RNA gene,partial sequence; chloroplast.
HM132945	STB17	178	70	OXY107	Biosope_T120_16S_OXY107	20	Stramenopiles	Pelagophyceae			HM133308	FJ649273	99.08%	Uncultured phototrophic eukaryote clone STB1_25m_D2 16S ribosomal RNA gene,partial sequence; chloroplast.
HM132946	STB17	178	70	OXY107	Biosope_T120_16S_OXY107	21	Stramenopiles	Pelagophyceae			HM133308	FJ649273	99.74%	Uncultured phototrophic eukaryote clone STB1_25m_D2 16S ribosomal RNA gene,partial sequence; chloroplast.
HM132947	STB17	178	70	OXY107	Biosope_T120_16S_OXY107	24	Stramenopiles	Pelagophyceae			HM133308	FJ649273	99.74%	Uncultured phototrophic eukaryote clone STB1_25m_D2 16S ribosomal RNA gene,partial sequence; chloroplast.
HM132948	STB17	178	70	OXY107	Biosope_T120_16S_OXY107	25	Stramenopiles	Pelagophyceae			HM133308	FJ649273	99.74%	Uncultured phototrophic eukaryote clone STB1_25m_D2 16S ribosomal RNA gene,partial sequence; chloroplast.
HM132949	STB17	178	70	OXY107	Biosope_T120_16S_OXY107	26	Stramenopiles	Pelagophyceae			HM133308	FJ649273	99.74%	Uncultured phototrophic eukaryote clone STB1_25m_D2 16S ribosomal RNA gene,partial sequence; chloroplast.
HM132950	STB17	178	70	OXY107	Biosope_T120_16S_OXY107	27	Stramenopiles	Pelagophyceae			HM133308	FJ649273	99.74%	Uncultured phototrophic eukaryote clone STB1_25m_D2 16S ribosomal RNA gene,partial sequence; chloroplast.
HM132951	STB17	178	70	OXY107	Biosope_T120_16S_OXY107	30	Stramenopiles	Pelagophyceae			HM133308	FJ649273	99.74%	Uncultured phototrophic eukaryote clone STB1_25m_D2 16S ribosomal RNA gene,partial sequence; chloroplast.
HM132952	STB17	178	70	PLA491	Biosope_T120_16S_PLA491	1	stramenopiles	Chrysophyceae			HM133478	EF052159	98.80%	Uncultured chrysophyte clone MC615-92 16S ribosomal RNA gene, partialsequence; plastid.
HM132953	STB17	178	70	PLA491	Biosope_T120_16S_PLA491	3	Chlorophyta	Prasinophyceae	clade 16S-VIII		HM133550	FJ649309	99.60%	Uncultured phototrophic eukaryote clone STB17_30m_H5 16S ribosomal RNAgene, partial sequence; chloroplast.
HM132954	STB17	178	70	PLA491	Biosope_T120_16S_PLA491	7	Chlorophyta	Prasinophyceae	clade 16S-VIII		HM133550	EU249955	100.00%	Uncultured phototrophic eukaryote clone PmeaH2OG10 16S ribosomal RNA gene,partial sequence; chloroplast.
HM132955	STB17	178	70	PLA491	Biosope_T120_16S_PLA491	8	Chlorophyta	Prasinophyceae	clade 16S-VIII		HM133550	FJ649309	99.73%	Uncultured phototrophic eukaryote clone STB17_30m_H5 16S ribosomal RNAgene, partial sequence; chloroplast.
HM132956	STB17	178	70	PLA491	Biosope_T120_16S_PLA491	9	Chlorophyta	Prasinophyceae	clade 16S-VIII		HM133550	FJ649309	99.60%	Uncultured phototrophic eukaryote clone STB17_30m_H5 16S ribosomal RNAgene, partial sequence; chloroplast.
HM132957	STB17	178	70	PLA491	Biosope_T120_16S_PLA491	10	Chlorophyta	Prasinophyceae	clade 16S-VIII		HM133550	FJ649309	99.73%	Uncultured phototrophic eukaryote clone STB17_30m_H5 16S ribosomal RNAgene, partial sequence; chloroplast.
HM132958	STB17	178	70	PLA491	Biosope_T120_16S_PLA491	11	Chlorophyta	Prasinophyceae	clade 16S-VIII		HM133550	FJ649309	99.60%	Uncultured phototrophic eukaryote clone STB17_30m_H5 16S ribosomal RNAgene, partial sequence; chloroplast.
HM132959	STB17	178	70	PLA491	Biosope_T120_16S_PLA491	12	Chlorophyta	Prasinophyceae	clade 16S-VIII		HM133550	FJ649309	99.87%	Uncultured phototrophic eukaryote clone STB17_30m_H5 16S ribosomal RNAgene, partial sequence; chloroplast.
HM132960	STB17	178	70	PLA491	Biosope_T120_16S_PLA491	13	Chlorophyta	Prasinophyceae	clade 16S-VIII		HM133550	EU249955	99.46%	Uncultured phototrophic eukaryote clone PmeaH2OG10 16S ribosomal RNA gene,partial sequence; chloroplast.
HM132961	STB17	178	70	PLA491	Biosope_T120_16S_PLA491	14	Chlorophyta	Prasinophyceae	clade 16S-VIII		HM133550	FJ649309	99.73%	Uncultured phototrophic eukaryote clone STB17_30m_H5 16S ribosomal RNAgene, partial sequence; chloroplast.
HM132962	STB17	178	70	PLA491	Biosope_T120_16S_PLA491	15	stramenopiles	Chrysophyceae			HM133470	EF574962	98.80%	Uncultured bacterium clone S25_1306 16S ribosomal RNA gene, partialsequence.
HM132963	STB17	178	70	PLA491	Biosope_T120_16S_PLA491	16	stramenopiles	Chrysophyceae			HM133470	EF574962	98.79%	Uncultured bacterium clone S25_1306 16S ribosomal RNA gene, partialsequence.
HM132964	STB17	178	70	PLA491	Biosope_T120_16S_PLA491	17	Chlorophyta	Prasinophyceae	clade 16S-VIII		HM133550	FJ649309	99.87%	Uncultured phototrophic eukaryote clone STB17_30m_H5 16S ribosomal RNAgene, partial sequence; chloroplast.
HM132965	STB17	178	70	PLA491	Biosope_T120_16S_PLA491	18	Chlorophyta	Prasinophyceae	clade 16S-VIII		HM133550	FJ649309	99.73%	Uncultured phototrophic eukaryote clone STB17_30m_H5 16S ribosomal RNAgene, partial sequence; chloroplast.
HM132966	STB17	178	70	PLA491	Biosope_T120_16S_PLA491	19	Chlorophyta	Prasinophyceae	clade 16S-VIII		HM133550	EU249955	99.87%	Uncultured phototrophic eukaryote clone PmeaH2OG10 16S ribosomal RNA gene,partial sequence; chloroplast.
HM132967	STB17	178	70	PLA491	Biosope_T120_16S_PLA491	22	Chlorophyta	Prasinophyceae	clade 16S-VIII		HM133550	FJ649309	99.87%	Uncultured phototrophic eukaryote clone STB17_30m_H5 16S ribosomal RNAgene, partial sequence; chloroplast.
HM132968	STB17	178	70	PLA491	Biosope_T120_16S_PLA491	23	Chlorophyta	Prasinophyceae	clade 16S-VIII		HM133550	EU249955	99.73%	Uncultured phototrophic eukaryote clone PmeaH2OG10 16S ribosomal RNA gene,partial sequence; chloroplast.
HM132969	STB17	178	70	PLA491	Biosope_T120_16S_PLA491	24	Chlorophyta	Prasinophyceae	clade 16S-VIII		HM133550	FJ649309	99.87%	Uncultured phototrophic eukaryote clone STB17_30m_H5 16S ribosomal RNAgene, partial sequence; chloroplast.
HM132970	STB17	178	70	PLA491	Biosope_T120_16S_PLA491	25	Chlorophyta	Prasinophyceae	clade 16S-VIII		HM133550	EU249955	99.87%	Uncultured phototrophic eukaryote clone PmeaH2OG10 16S ribosomal RNA gene,partial sequence; chloroplast.
HM132971	STB17	178	70	PLA491	Biosope_T120_16S_PLA491	27	Chlorophyta	Prasinophyceae	clade 16S-VIII		HM133550	FJ649309	99.06%	Uncultured phototrophic eukaryote clone STB17_30m_H5 16S ribosomal RNAgene, partial sequence; chloroplast.
HM132972	STB17	178	70	PLA491	Biosope_T120_16S_PLA491	28	Chlorophyta	Prasinophyceae	clade 16S-VIII		HM133550	EU249955	99.87%	Uncultured phototrophic eukaryote clone PmeaH2OG10 16S ribosomal RNA gene,partial sequence; chloroplast.
HM132973	STB17	178	70	PLA491	Biosope_T120_16S_PLA491	29	Chlorophyta	Prasinophyceae	clade 16S-VIII		HM133550	FJ649309	99.60%	Uncultured phototrophic eukaryote clone STB17_30m_H5 16S ribosomal RNAgene, partial sequence; chloroplast.
HM132974	STB17	178	70	PLA491	Biosope_T120_16S_PLA491	30	Haptophyta	Prymnesiophyceae			HM133494	FJ649305	98.54%	Uncultured phototrophic eukaryote clone STB17_30m_H1 16S ribosomal RNAgene, partial sequence; chloroplast.
HM132975	STB17	178	70	PLA491	Biosope_T120_16S_PLA491	31	Chlorophyta	Prasinophyceae	clade 16S-VIII		HM133550	EU249955	99.87%	Uncultured phototrophic eukaryote clone PmeaH2OG10 16S ribosomal RNA gene,partial sequence; chloroplast.
HM132976	STB17	178	70	PLA491	Biosope_T120_16S_PLA491	34	Haptophyta	Prymnesiophyceae			HM133339	DQ395642	99.74%	Uncultured organism clone ctg_CGOF234 16S ribosomal RNA gene, partialsequence.
HM132977	STB17	178	70	PLA491	Biosope_T120_16S_PLA491	36	Chlorophyta	Prasinophyceae	clade 16S-VIII		HM133550	FJ649309	99.87%	Uncultured phototrophic eukaryote clone STB17_30m_H5 16S ribosomal RNAgene, partial sequence; chloroplast.

Accession	station	CTD cast	depth	primer_forward	Clone_library	Clone	Division	Class	Order	Genus	OTU	BLAST Accession	BLAST % ID	BLAST Description
HM132978	STB17	178	70	PLA491	Biosope_T120_16S_PLA491	37	Chlorophyta	Prasinophyceae	clade 16S-VIII		HM133550	FJ649309	99.87%	Uncultured phototrophic eukaryote clone STB17_30m_H5 16S ribosomal RNAgene, partial sequence; chloroplast.
HM132979	STB17	178	70	PLA491	Biosope_T120_16S_PLA491	38	Chlorophyta	Prasinophyceae	clade 16S-VIII		HM133550	FJ649309	99.73%	Uncultured phototrophic eukaryote clone STB17_30m_H5 16S ribosomal RNAgene, partial sequence; chloroplast.
HM132980	STB17	178	70	PLA491	Biosope_T120_16S_PLA491	39	Chlorophyta	Prasinophyceae	clade 16S-VIII		HM133550	FJ649309	99.73%	Uncultured phototrophic eukaryote clone STB17_30m_H5 16S ribosomal RNAgene, partial sequence; chloroplast.
HM132981	STB17	178	70	PLA491	Biosope_T120_16S_PLA491	40	Chlorophyta	Prasinophyceae	clade 16S-VIII		HM133550	FJ649309	98.67%	Uncultured phototrophic eukaryote clone STB17_30m_H5 16S ribosomal RNAgene, partial sequence; chloroplast.
HM132982	STB17	178	70	PLA491	Biosope_T120_16S_PLA491	41	Chlorophyta	Prasinophyceae	clade 16S-VIII		HM133550	FJ649309	99.87%	Uncultured phototrophic eukaryote clone STB17_30m_H5 16S ribosomal RNAgene, partial sequence; chloroplast.
HM132983	STB17	178	70	PLA491	Biosope_T120_16S_PLA491	42	Chlorophyta	Prasinophyceae	clade 16S-VIII		HM133550	EU249955	99.73%	Uncultured phototrophic eukaryote clone PmeaH2OG10 16S ribosomal RNA gene,partial sequence; chloroplast.
HM132984	STB17	178	70	PLA491	Biosope_T120_16S_PLA491	43	Chlorophyta	Prasinophyceae	clade 16S-VIII		HM133550	FJ649309	99.73%	Uncultured phototrophic eukaryote clone STB17_30m_H5 16S ribosomal RNAgene, partial sequence; chloroplast.
HM132985	STB17	178	70	PLA491	Biosope_T120_16S_PLA491	45	Chlorophyta	Prasinophyceae	clade 16S-VIII		HM133550	FJ649309	99.73%	Uncultured phototrophic eukaryote clone STB17_30m_H5 16S ribosomal RNAgene, partial sequence; chloroplast.
HM132986	STB17	178	70	PLA491	Biosope_T120_16S_PLA491	47	Chlorophyta	Prasinophyceae	clade 16S-VIII		HM133550	FJ649309	99.46%	Uncultured phototrophic eukaryote clone STB17_30m_H5 16S ribosomal RNAgene, partial sequence; chloroplast.
HM132987	STB17	178	70	PLA491	Biosope_T120_16S_PLA491	49	Chlorophyta	Prasinophyceae	clade 16S-VIII		HM133550	EU249955	100.00%	Uncultured phototrophic eukaryote clone PmeaH2OG10 16S ribosomal RNA gene,partial sequence; chloroplast.
HM133043	UPW1	198	35	OXY107	Biosope_T148_16S_OXY107	6	stramenopiles	Bacillariophyceae		Chaetoceros	HM133064	FJ002204	99.27%	Chaetoceros sp. C134 16S ribosomal RNA gene, partial sequence; chloroplast.
HM133044	UPW1	198	35	OXY107	Biosope_T148_16S_OXY107	16	Stramenopiles	Dictyochophyceae			HM133300	FJ826020	99.52%	Uncultured phototrophic eukaryote clone BM1-8-32 16S ribosomal RNA gene,partial sequence; plastid.
HM133045	UPW1	198	35	OXY107	Biosope_T148_16S_OXY107	36	Stramenopiles	Dictyochophyceae			HM133342	FJ649290	98.50%	Uncultured phototrophic eukaryote clone STB1_25m_F8 16S ribosomal RNA gene,partial sequence; chloroplast.
HM133046	UPW1	198	35	PLA491	Biosope_T148_16S_PLA491	2	Stramenopiles	Dictyochophyceae			HM133342	FJ649290	98.24%	Uncultured phototrophic eukaryote clone STB1_25m_F8 16S ribosomal RNA gene,partial sequence; chloroplast.
HM133047	UPW1	198	35	PLA491	Biosope_T148_16S_PLA491	3	Chlorophyta	Mamelliophyceae	Mamiellales	Micromonas	HM133063	FJ826259	99.86%	Uncultured phototrophic eukaryote clone A6-1-51 16S ribosomal RNA gene,partial sequence; plastid.
HM133048	UPW1	198	35	PLA491	Biosope_T148_16S_PLA491	6	Stramenopiles	Dictyochophyceae			HM133342	FJ649290	98.37%	Uncultured phototrophic eukaryote clone STB1_25m_F8 16S ribosomal RNA gene,partial sequence; chloroplast.
HM133049	UPW1	198	35	PLA491	Biosope_T148_16S_PLA491	7	Chlorophyta	Mamelliophyceae	Mamiellales	Bathycoccus	HM133057	U70715	99.71%	Unidentified prasinophyte OM5 16S ribosomal RNA gene, partialsequence.
HM133050	UPW1	198	35	PLA491	Biosope_T148_16S_PLA491	10	Haptophyta	Prymnesiophyceae			HM133266	EF052049	100.00%	Uncultured haptophyte clone 250304-53 16S ribosomal RNA gene, partialsequence; plastid.
HM133051	UPW1	198	35	PLA491	Biosope_T148_16S_PLA491	11	Chlorophyta	Mamelliophyceae	Mamiellales	Ostreococcus	HM133051	FJ826251	99.58%	Uncultured marine bacterium clone A6-1-43 16S ribosomal RNA gene, partialsequence.
HM133052	UPW1	198	35	PLA491	Biosope_T148_16S_PLA491	12	stramenopiles	Bacillariophyceae			HM133064	FJ355365	99.70%	Uncultured organism clone 051011_S1_W_T_SDP_006 small subunit ribosomal RNAgene, partial sequence; chloroplast.
HM133053	UPW1	198	35	PLA491	Biosope_T148_16S_PLA491	13	Haptophyta	Prymnesiophyceae			HM133266	EF052049	99.73%	Uncultured haptophyte clone 250304-53 16S ribosomal RNA gene, partialsequence; plastid.
HM133054	UPW1	198	35	PLA491	Biosope_T148_16S_PLA491	14	Haptophyta	Prymnesiophyceae			HM133503	EF573851	99.21%	Uncultured bacterium clone S25_195 16S ribosomal RNA gene, partialsequence.
HM133055	UPW1	198	35	PLA491	Biosope_T148_16S_PLA491	16	stramenopiles	Chrysophyceae			HM133058	EF052043	99.27%	Uncultured chrysophyte clone 250304-46 16S ribosomal RNA gene, partialsequence; plastid.
HM133056	UPW1	198	35	PLA491	Biosope_T148_16S_PLA491	20	Haptophyta	Prymnesiophyceae			HM133266	EF573710	99.74%	Uncultured bacterium clone S25_54 16S ribosomal RNA gene, partial sequence.
HM133057	UPW1	198	35	PLA491	Biosope_T148_16S_PLA491	21	Chlorophyta	Mamelliophyceae	Mamiellales	Bathycoccus	HM133057	U70715	99.60%	Unidentified prasinophyte OM5 16S ribosomal RNA gene, partialsequence.
HM133058	UPW1	198	35	PLA491	Biosope_T148_16S_PLA491	22	stramenopiles	Chrysophyceae			HM133058	EF052043	99.21%	Uncultured chrysophyte clone 250304-46 16S ribosomal RNA gene, partialsequence; plastid.
HM133059	UPW1	198	35	PLA491	Biosope_T148_16S_PLA491	23	stramenopiles	Dictyochophyceae			HM133342	FJ649290	98.66%	Uncultured phototrophic eukaryote clone STB1_25m_F8 16S ribosomal RNA gene,partial sequence; chloroplast.
HM133060	UPW1	198	35	PLA491	Biosope_T148_16S_PLA491	24	Chlorophyta	Mamelliophyceae	Mamiellales	Bathycoccus	HM133057	U70715	99.55%	Unidentified prasinophyte OM5 16S ribosomal RNA gene, partialsequence.
HM133061	UPW1	198	35	PLA491	Biosope_T148_16S_PLA491	25	Haptophyta	Prymnesiophyceae			HM133266	EF052229	98.95%	Uncultured haptophyte clone MC622-69 16S ribosomal RNA gene, partialsequence; plastid.
HM133062	UPW1	198	35	PLA491	Biosope_T148_16S_PLA491	26	Haptophyta	Prymnesiophyceae			HM133065	EF052229	98.96%	Uncultured haptophyte clone MC622-69 16S ribosomal RNA gene, partialsequence; plastid.
HM133063	UPW1	198	35	PLA491	Biosope_T148_16S_PLA491	29	Chlorophyta	Mamelliophyceae	Mamiellales	Micromonas	HM133063	FJ826259	99.87%	Uncultured phototrophic eukaryote clone A6-1-51 16S ribosomal RNA gene,partial sequence; plastid.
HM133064	UPW1	198	35	PLA491	Biosope_T148_16S_PLA491	31	stramenopiles	Bacillariophyceae			HM133064	FJ355041	98.05%	Uncultured organism clone Lc2yS22_ML_277 small subunit ribosomal RNA gene,partial sequence; chloroplast.
HM133065	UPW1	198	35	PLA491	Biosope_T148_16S_PLA491	32	Haptophyta	Prymnesiophyceae			HM133065	EF052229	98.95%	Uncultured haptophyte clone MC622-69 16S ribosomal RNA gene, partialsequence; plastid.
